# ASTRO: Automated Spatial-Transcriptome whole RNA Output

**DOI:** 10.1093/bioinformatics/btaf688

**Published:** 2026-01-06

**Authors:** Dingyao Zhang, Zhiyuan Chu, Yiran Huo, Yunzhe Jiang, Yuhang Chen, Zhiliang Bai, Rong Fan, Jun Lu, Mark Gerstein

**Affiliations:** Department of Genetics, Yale School of Medicine, New Haven, CT 06520, United States; Program in Computational Biology and Bioinformatics, Yale University, New Haven, CT 06520, United States; Program in Computational Biology and Bioinformatics, Yale University, New Haven, CT 06520, United States; Department of Biostatistics, Yale School of Public Health, New Haven, CT 06520, United States; Program in Computational Biology and Bioinformatics, Yale University, New Haven, CT 06520, United States; Program in Computational Biology and Bioinformatics, Yale University, New Haven, CT 06520, United States; Department of Biomedical Engineering, Yale University, New Haven, CT 06520, United States; Department of Biomedical Engineering, Yale University, New Haven, CT 06520, United States; Department of Pathology, Yale School of Medicine, New Haven, CT 06520, United States; Human and Translational Immunology, Yale School of Medicine, New Haven, CT 06520, United States; Yale Stem Cell Center, Yale School of Medicine, New Haven, CT 06520, United States; Yale Cancer Center and Yale Center for RNA Science and Medicine, Yale School of Medicine, New Haven, CT 06520, United States; Department of Genetics, Yale School of Medicine, New Haven, CT 06520, United States; Yale Stem Cell Center, Yale School of Medicine, New Haven, CT 06520, United States; Yale Cooperative Center of Excellence in Hematology, Yale School of Medicine, New Haven, CT 06520, United States; Yale Cancer Center and Yale Center for RNA Science and Medicine, Yale School of Medicine, New Haven, CT 06520, United States; Program in Computational Biology and Bioinformatics, Yale University, New Haven, CT 06520, United States; Yale Cancer Center and Yale Center for RNA Science and Medicine, Yale School of Medicine, New Haven, CT 06520, United States; Department of Molecular Biophysics and Biochemistry, Yale University, New Haven, CT 06520, United States

## Abstract

**Motivation:**

Despite significant advances in spatial transcriptomics, the analysis of formalin-fixed paraffin-embedded (FFPE) tissues, which constitute most clinically available samples, remains challenging. Additionally, capturing both coding and non-coding RNAs in a spatial context poses significant challenges. We recently introduced Patho-DBiT, a technology designed to address these unmet needs. However, the marked differences between Patho-DBiT and existing spatial transcriptomics protocols necessitate specialized computational tools for comprehensive whole-transcriptome analysis in FFPE samples.

**Results:**

Here, we present ASTRO, an automated pipeline developed to process spatial transcriptomics data. In addition to supporting standard datasets, ASTRO is optimized for whole-transcriptome analyses of FFPE samples, enabling the detection of various RNA species, including non-coding RNAs such as miRNAs. To compensate for the reduced RNA quality in FFPE tissues, ASTRO incorporates a specialized filtering step and optimizes spatial barcode calling, increasing the mapping rate. These optimizations allow ASTRO to spatially quantify coding and non-coding RNA species in the entire transcriptome and achieve robust performance in FFPE samples.

**Availability and implementation:**

Codes are available at GitHub (https://github.com/gersteinlab/ASTRO) and Zenodo (doi: 10.5281/zenodo.17913760).

## 1 Introduction

With spatial information incorporated, spatial transcriptomics technologies have revolutionized transcriptomic analyses in recent years, opening a new era of genomics research (Baysoy *et al.* 2023, Bressan *et al.* 2023, Deng *et al.* 2023, Chen *et al.* 2024). Although the field has made remarkable strides, most spatial transcriptomics methods still focus on mRNAs and do not capture the entire transcriptome. Yet, extensive evidence shows that various non-coding RNAs play critical biological roles in tissues, underscoring the importance of spatially profiling these molecules (Mattick *et al.* 2023, Chen and Kim 2024). Furthermore, formalin-fixed paraffin-embedded (FFPE) tissues are commonly used in hospital pathology departments, and the extensive collections of FFPE blocks represent an invaluable resource for translational research (Blow 2007). However, sequencing these samples faces significant challenges, including RNA fragmentation, degradation, chemical modifications, and the loss of poly-A tails, especially when stored under suboptimal conditions (Levin *et al.* 2020).

To address this gap, we recently developed **patho**logy-compatible **d**eterministic **b**arcoding **i**n **t**issue (Patho-DBiT) (Bai *et al.* 2024), which leverages *in situ* polyadenylation to enable spatial whole-transcriptome sequencing in clinically archived FFPE tissues. However, the significant differences between traditional mRNA sequencing and whole-transcriptome sequencing, as well as between fresh-frozen samples and FFPE samples, necessitate a specialized computational pipeline to facilitate comprehensive spatial profiling of whole transcriptomics in these clinical-level tissues.

**Figure 1.**
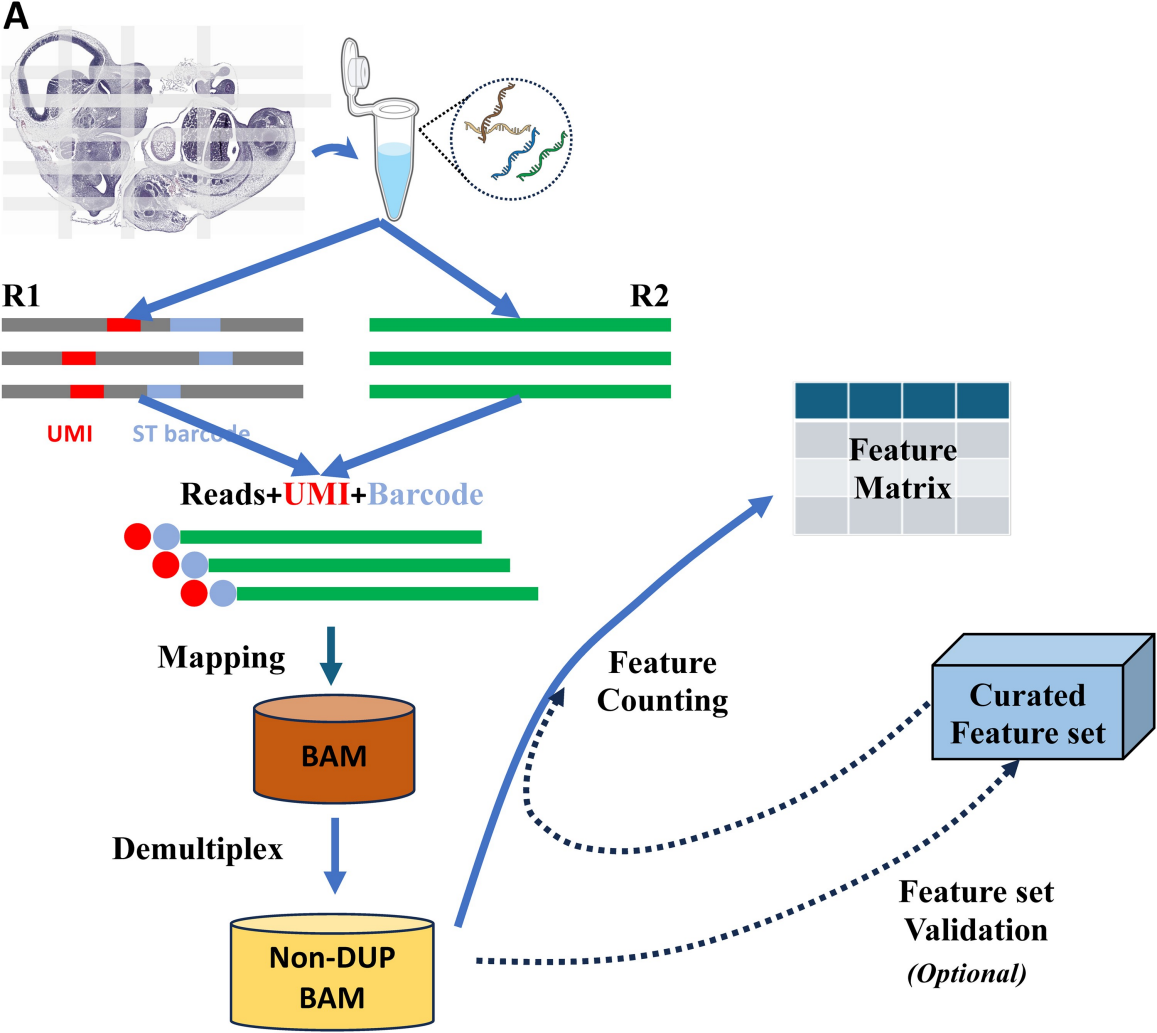
Workflow of ASTRO. (A) A schematic overview of the ASTRO workflow is shown. Spatial transcriptomics data typically comprise paired FASTQ reads: read1 (or R1), containing spatial barcodes and UMIs, and read2 (or R2), containing transcriptome RNA sequences. The pipeline merges R2 information with R1 to create a combined FASTQ file, which is then mapped to the genome. After mapping, demultiplexing is performed on the resulting BAM file to generate a non-duplicate (Non-DUP) BAM file for feature counting. An optional validation step can be applied to produce a curated set containing valid features. The final feature matrix is then generated using either the curated set (if the validation step is applied) or the original feature set. Panel A includes an illustration from NIAID NIH BioArt Source (bioart.niaid.nih.gov/bioart/143).

In this study, we develop and implement ASTRO: (**A**utomated **S**patial-**T**ranscriptome whole **R**NA **O**utput), a spatial transcriptomics mapping pipeline optimized for both coding and non-coding RNAs (ncRNAs) as well as FFPE samples (Fig. 1). The pipeline employs a scoring system to capture ncRNAs at different maturation stages and removes incorrect or non-expressed ncRNA annotations, enabling robust spatial profiling of a spectrum of ncRNAs. Additionally, ASTRO distinguishes intron reads from exon reads, adding further depth to RNA biology analyses. Because FFPE samples often exhibit severe RNA degradation (Levin *et al.* 2020), ASTRO maximizes the information obtained from each sample by tolerating insertions and deletions (indels) and variations in barcode regions during demultiplexing. Subsequently, ASTRO deploys a post-alignment filter to eliminate invalid reads. By integrating these advanced features, we developed a robust pipeline specifically tailored for spatial whole-transcriptome analysis in FFPE tissues.

## 2 Materials and methods

### 2.1 The ASTRO pipeline

An overview of the ASTRO pipeline is presented in Fig. 1. ASTRO takes FASTQ files as input and produces a gene-pixel (feature-location) matrix. The pipeline supports various RNA species, including mRNAs, lncRNAs, tRNAs, and miRNAs. Because of the biological differences between introns and exons, ASTRO separates intron reads and exon reads by default, facilitating downstream analyses such as RNA velocity. In addition to generating the final matrix, ASTRO outputs intermediate files (e.g. BAM files) for further analyses. The ASTRO pipeline is available as a Python package in the GitHub repository: https://github.com/gersteinlab/ASTRO and Zenodo (https://zenodo.org/records/17913760).

### 2.2 Demultiplexing and genomic alignment in ASTRO

During demultiplexing, the pipeline first utilizes the structure of read 1 (R1) to guide processing. It then applies Cutadapt to trim reads based on adapters and linkers, partitioning reads into segments corresponding to unique molecular identifiers (UMIs) and potential spatial barcodes (Martin 2011). Because R1 may contain indels, the pipeline extracts sequences from an expanded region for spatial barcodes. To handle potential sequencing errors, ASTRO leverages Bowtie2 or STAR (Langmead and Salzberg 2012, Dobin *et al.* 2013) to build a reference of valid spatial barcodes and align the spliced R1 spatial barcode sequences to this reference, thereby determining each read’s spatial barcode identity. All spatial barcodes were pre-designed and therefore defined in sequence. During barcode mapping, only the best match is retained, and reads mapping equally well to multiple barcodes are discarded as ambiguous reads.

### 2.3 Feature counting in ASTRO

#### 2.3.1 Establishment of whole RNA reference

For genome mapping in various research projects, the GENCODE database is among the most widely used resources (Mudge *et al.* 2025). However, its non-coding RNA annotation remains incomplete. It omits certain non-coding RNA species and, in other cases, lacks sufficient detail (e.g. mature 5p/3p miRNA isoforms and codon differences among tRNAs). To address these gaps, we created specialized GTF files for our pipeline by compiling genomic data from multiple databases, including GENCODE, miRbase, piRNAdb, GtRNAdb, and RNAcentral (Kozomara and Griffiths-Jones 2011, Chan and Lowe 2016, Wang *et al.* 2019, Sweeney *et al.* 2021, Mudge *et al.* 2025). We then merged similar records into single entries to form comprehensive GTF files. Further details on this procedure are available in the Supplementary Materials (Tables 1 and 2, available as supplementary data at *Bioinformatics* online). We produced these specialized GTF files for two genome assemblies: mouse mm39 and human GRCh38.

#### 2.3.2 Assign reads with overlapping annotations

For RNA fragments mapping to multiple overlapping annotations, an “overlap score” was calculated using the formula (*L_o_*−*L_no_*)/*L_a_*, where *L_o_* denotes the overlapped length between the query RNA fragment and an annotation, *L_no_* represents the non-overlapped length of the query RNA fragment, and L_a_ is the total length of the genomic annotation. The annotation with the highest overlap score was considered the true annotation for the RNA fragment. In our specialized GTF, exon and transcripts are marked as different records, allowing the method to differentiate between exon and intron features. If a read has the highest overlap score with an exon feature, it is classified as exon mapping. Conversely, if a read has the highest overlap score with an intron feature, it is classified as mapped to an intron. If multiple features receive the same overlap score, the read is considered multi-mapping and counted as a multi-mapping feature. This feature is named by concatenating the feature names with identical scores using plus signs (e.g. “gene1+gene2+…+geneN”).

#### 2.3.3 Adjustment of valid features

The previous assignment step depends heavily on high-quality GTF files, as invalid entries can lead to an excessive number of spurious features in the gene expression matrix. However, due to the strong tissue specificity of many non-coding RNAs (Ludwig *et al.* 2016, Statello *et al.* 2021), it is necessary to adjust the GTF files for each dataset. The main principle behind this adjustment is that a genuine RNA structure typically displays a significantly higher read depth than its background regions, whereas a structure formed by randomly fragmented background RNA should not exhibit a substantial change in read depth. To implement this principle, we conduct an examination, including a statistical test, for each feature. Further details on this validation process are provided in the Supplementary Materials, available as supplementary data at *Bioinformatics* online.

### 2.4 Evaluating the performance of ASTRO

#### 2.4.1 Collection of spatial-transcriptome datasets

To assess the performance of ASTRO, we used four publicly available spatial-transcriptome datasets from our previous study (Bai *et al.* 2024). The first dataset is a clinical extranodal marginal zone lymphoma of mucosa-associated lymphoid tissue (MALT) tumor biopsy, featuring 10 000 spots (or pixels) at a 20 µm pixel size. The second dataset is an FFPE biopsy of a healthy donor lymph node. The other two datasets are replicates of embryonic day 13 mouse embryo FFPE sections at a 50 µm pixel size; these two replicates were collected from two adjacent slides.

#### 2.4.2 Comparison between ASTRO and existing methods

To evaluate the impact of ASTRO on downstream analyses, we compared it against existing spatial transcriptomics pipelines. Currently, two widely used pipelines are the ST-pipeline and the Space Ranger (Navarro *et al.* 2017, 10XGenomics 2024); however, Space Ranger is only compatible with 10× Genomics data. Moreover, the 10× FFPE spatial assay uses RNA-assisted probe ligation: two probes targeting the same target RNA are ligated when the target exists, generating PCR-expandable ligated probes for sequencing. This chemistry is not compatible with ASTRO. Consequently, we restricted our benchmarking on spatial FFPE datasets to the ST-pipeline (version 1.8.1) which we installed via pip. For Space Ranger, we performed a benchmark using non-FFPE 10× Genomics spatial datasets; details are provided in Section 3.2. We evaluated pipeline performance on downstream analyses using two approaches. First, we aligned the clustering results with hematoxylin and eosin (H&E) staining to compare tissue structures; a more refined pipeline should capture more detailed structures. Second, we employed three quantitative metrics previously used in single-cell clustering evaluations, the Silhouette score, the Calinski–Harabasz index, and the Davies–Bouldin index, to assess clustering performance (Jiang *et al.* 2018, Leng *et al.* 2022, Yu *et al.* 2022, Møller and Madsen 2023). Note that higher Silhouette and Calinski–Harabasz scores indicate superior clustering performance, while lower Davies–Bouldin index scores indicate better performance. This assessment was conducted on both the full dataset and a 50% subsampled dataset. Further details on the downsampling process are provided in the Supplementary Materials, available as supplementary data at *Bioinformatics* online.

### 2.5 Implementation of ASTRO

ASTRO is implemented in Python (≥3.8.16) and uses only the standard library. Although no extra Python modules are required, ASTRO does depend on external command-line tools, including BEDTools (≥2.31.1), Cutadapt (≥4.0), STAR (≥2.7.9a), and SAMtools (≥1.20) (Li *et al.* 2009, Martin 2011, Quinlan 2014). ASTRO supports parallel execution via Python built-in multiprocessing module. With this parallelism, ASTRO is able to complete analysis in a reasonable time with modest CPU and memory usage. Further details on memory requirements and runtime are provided in Table 3, available as supplementary data at *Bioinformatics* online.

## 3 Results

### 3.1 Performance of ASTRO in FFPE spatial datasets

**Figure 2:**
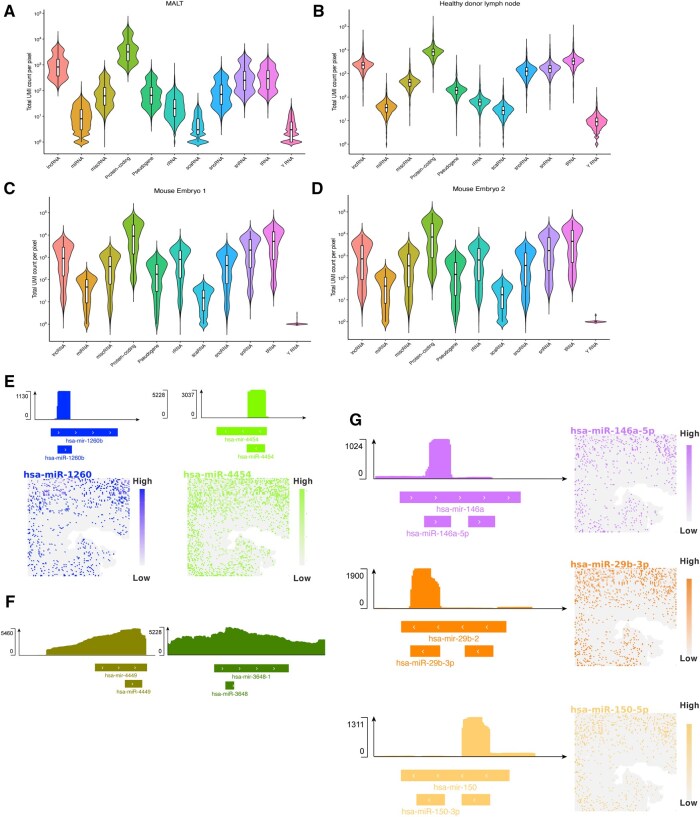
ASTRO enables whole-transcriptome analysis, including miRNAs. (A–D) Violin plots show the ability of ASTRO to detect various RNA species, with the *y*-axis indicating the UMI count assigned to each species (MALT, marginal zone lymphoma of mucosa-associated lymphoid tissue). (E) Examples of miRNAs, including hsa-miR-4454 and hsa-miR-1260b, that are significantly enriched compared to background levels. Only reads on the same strand as the miRNA annotation are retained. The corresponding spatial distributions of these miRNAs are shown alongside. (F) Examples of miRNAs, including hsa-miR-3648 and hsa-miR-4449, that are not significantly enriched above background levels. Only reads on the same strand as the miRNA annotation are retained. (G) Examples of miRNAs, including hsa-miR-146a-5p, hsa-miR-29b-3p, and hsa-miR-150a-5p, whose isoforms display distinct patterns. Only reads on the same strand as the miRNA annotation are retained. The corresponding spatial distributions of these miRNAs are shown alongside.

**Figure 3:**
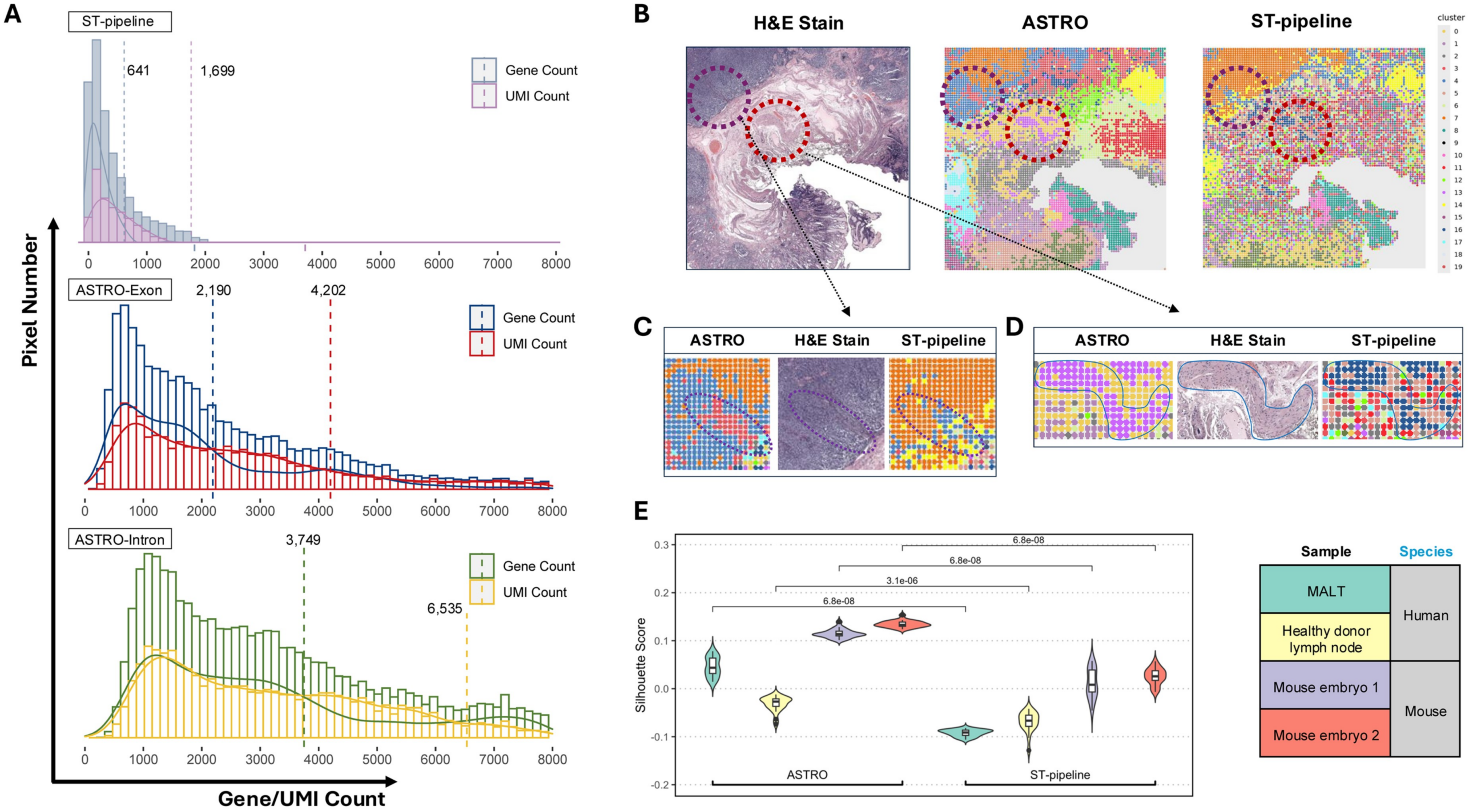
Benchmarking of downstream analysis based on different pipelines. (A) Distribution of detected gene/UMI counts per spatial pixel from different sources. Dashed lines indicate the average levels of gene or UMI counts. (B) Comparison of H&E images, ASTRO-based clustering, and ST-pipeline-based clustering for the MALT sample. The circles highlight two subtle structures in the tissue. (C) Enlarged view of the region within the upper circle in (B). (D) Enlarged view of the region within the lower circle in (B). (E) Quantitative measurement of downstream analyses. Four samples were analysed separately using ASTRO and ST-pipeline. Silhouette scores for each condition are shown.

To demonstrate the performance of ASTRO in whole transcriptome, we deployed it across all four spatial-transcriptome samples. ASTRO successfully captured a wide range of RNA species across the datasets, including lncRNAs, miRNAs, protein-coding RNAs (mRNAs), rRNAs, scaRNAs, snoRNAs, snRNAs, tRNAs, Y RNAs, and miscRNAs. These RNA classes are shown in both violin plots and spatial expression maps, with ST-pipeline results provided for comparison (Fig. 2A–D and Figs 1 and 2, File 1, available as supplementary data at *Bioinformatics* online). Because piRNAs are highly germline-tissue-specific (Wang *et al.* 2024), we excluded them from these statistics. Beyond its broad RNA coverage, ASTRO filters out features that are not truly expressed. For instance, in the MALT sample, ASTRO identified various miRNAs while dismissing those inflated by background noise. Reads mapped to hsa-miR-4454 and hsa-miR-1260b were significantly enriched above background, indicating their validity (Fig. 2E). In contrast, reads mapped to hsa-mir-3648 and hsa-mir-4449 were not significantly enriched relative to neighboring regions, suggesting that they should not be assigned to these miRNAs (Fig. 2F). Moreover, ASTRO captures isoform-level differences. Many miRNAs have two mature isoforms (5p isoform and 3p isoform), and the expression patterns of these isoforms are known to be different. In the MALT dataset, hsa-miR-146a-5p, hsa-miR-29b-3p, and hsa-miR-150a-5p were identified as valid features in this dataset, consistent with their known isoform expression dominance from these miRNA genes (Fig. 2G). The spatial distributions of the ASTRO-validated miRNAs are also shown alongside their genomic read depth plots. In addition, compared with miRDeep2, a gold standard for miRNA quantification for bulk small RNA sequencing data, the pseudo-bulk miRNA expression levels obtained from ASTRO are highly correlated with those derived from miRDeep2 (Fig. 3A and B, available as supplementary data at *Bioinformatics* online). Details of this comparison are provided in the Supplementary Materials, available as supplementary data at *Bioinformatics* online.

ASTRO also enables quantification of spatial distributions of non-coding RNAs. For example, in the human MALT sample, hsa-miR-143-5p was enriched in smooth muscle regions and reduced in the lymphoma regions, whereas hsa-miR-142-5p was depleted in smooth muscle regions and increased in lymphoma regions. This pattern is consistent with established biology: hsa-miR-143-5p is highly expressed in smooth muscle, whereas hsa-miR-142-5p is enriched in immune/hematopoietic cells. Notably, both miRNAs were not detected by the ST-pipeline, indicating that ASTRO reveals tissue associated distributions of non-coding RNAs. missed by the ST-pipeline workflows (Fig. 3C–F, available as supplementary data at *Bioinformatics* online).

When we applied both ASTRO and the ST-pipeline to the same MALT dataset, the total number of captured reads differed. A key feature of ASTRO is its ability to distinguish between exons and introns, allowing it to capture more reads overall. In both exon-assigned and intron-assigned counts, ASTRO detected a higher gene/UMI count than ST-pipeline which does not distinguish introns from exons (Fig. 3A). To further compare the performance of ASTRO and the ST-pipeline, we applied each pipeline separately to the MALT sample. For the Silhouette score, Calinski–Harabasz index, and Davies–Bouldin index, ASTRO-based analysis achieved values of 0.1525, 1726.9187, and 1.4279, respectively, outperforming ST-pipeline-based analysis, which produced values of 0.0989, 985.0426, and 1.5817.

Overall, ASTRO exhibited a much lower noise level, as indicated by spatial clustering and statistical tests (Fig. 3B). Additionally, ASTRO identified detailed tissue structures that ST-pipeline missed (Fig. 3C and D). For example, in the upper circled region, the H&E image revealed a blood vessel within a B-cell lymphoma area. ASTRO clearly delineated a cell group, consistent with H&E staining, whereas ST-pipeline improperly split the region into multiple clusters. Also, in the lower circled region, ASTRO accurately captured a smooth muscle cell group, whereas ST-pipeline failed to identify any distinct structures. Similar patterns were observed in the healthy donor lymph node and mouse embryo samples. ASTRO detected more detailed lymph node architecture that was ignored by ST-pipeline. Although the two mouse embryo samples are adjacent tissue sections, ST-pipeline failed to resolve the two-lobed liver structure in replicate 2, likely because the liver region is smaller in this section. However, ASTRO successfully detected both lobes in both replicates (Fig. 4A–C, available as supplementary data at *Bioinformatics* online). Finally, we assessed all four samples using the Silhouette score, Calinski–Harabasz index, and Davies–Bouldin index. For all datasets, ASTRO-based results demonstrated superior clustering performance (Supplementary Materials, available as supplementary data at *Bioinformatics* online). When subsampling was performed, ASTRO outperformed ST-pipeline across nearly all metrics and samples (Fig. 3E and Fig. 4D and E, available as supplementary data at *Bioinformatics* online).

### 3.2 ASTRO across technologies and datasets

Although ASTRO was designed for spatial transcriptomics datasets of FFPE tissue sections, it is a flexible pipeline compatible with multiple technologies. To demonstrate this flexibility, we analysed datasets from STRS (McKellar *et al.* 2023), 10x Genomics Visium (Ji *et al.* 2020), Smart-seq Total (Isakova *et al.* 2021), DBiT-seq (fresh-frozen; non-FFPE) (Liu *et al.* 2020), and Slide-seq (Sampath Kumar *et al.* 2023). The selected technologies were chosen to span different assay types and library chemistries, demonstrating the flexibility of ASTRO. Smart-seq Total and STRS are total-RNA protocols, whereas the other platforms profile poly(A)-enriched libraries. Smart-seq Total operates at the single-cell level, and the others are at the spatial level. Detailed comparisons of technology characteristics (e.g. chemistry, tissue type) are provided in Table 4, available as supplementary data at *Bioinformatics* online, and scripts documenting the usage of ASTRO on these datasets are available on GitHub. All these five datasets use non-FFPE samples, in contrast to the FFPE Patho-DBiT datasets analysed in Section 2.4.2. Moreover, Slide-seq, DBiT-seq, Smart-seq Total, and 10x Genomics Visium are widely used and commercially available methods that provide ample publicly available datasets for ASTRO. In addition, to evaluate the performance of ASTRO, we conducted benchmarking analyses on these datasets. During benchmarking, we selected suitable baseline pipelines based on compatibility and ease of use. We used the ST-pipeline for DBiT-seq and Space Ranger for 10x Visium. For STRS, Smart-seq Total, and Slide-seq, we compared ASTRO against the author-provided expression matrices from their respective custom pipelines. Across all samples, ASTRO achieved better performance on three quantitative clustering metrics (the Silhouette score, the Calinski–Harabasz index, and the Davies–Bouldin index), indicating that ASTRO performs robustly across these datasets and reliably reveals spatial patterns of non-coding RNA expression (Supplementary Materials, Figs 5–9, available as supplementary data at *Bioinformatics* online). The spatial patterns of pixel-level clusters were also shown, although comparisons between methods at this visualization level were more challenging due to the absence of public H&E staining for some of the samples.

## 4 Conclusions and discussion

In this study, we developed the ASTRO pipeline and demonstrated its utility and performance across multiple datasets addressing diverse biological questions. Overall, ASTRO effectively quantifies on the spatial level the whole transcriptome, including non-coding RNAs, while capturing both RNA isoform details and RNA maturation stages in a spatial context. To the best of our knowledge, it is the first tool specifically designed for this purpose. Unlike most previous studies, which analysed non-coding RNAs separately using different references and mapping steps, our pipeline examines all non-coding RNAs simultaneously, thereby streamlining downstream analyses.

In addition, our pipeline is specialized for FFPE samples, making it particularly useful for spatial profiling using clinical archives. This enhancement, together with broader RNA species coverage, improves downstream analyses when using FFPE datasets. This is especially important in clinical settings, as many FFPE samples have been stored under suboptimal conditions for years (Matsunaga *et al.* 2022). Consequently, maximizing the amount of sequenced information is critical for reliable analysis of FFPE sample sequencing.

Although this pipeline focuses on FFPE spatial-transcriptome data, ASTRO is also compatible with non-FFPE spatial-transcriptome datasets and single-cell sequencing data. Furthermore, if these datasets provide whole-transcriptome coverage (e.g. VASA-seq or STRS) (Salmen *et al.* 2022, McKellar *et al.* 2023), ASTRO can achieve the quantification of various RNA species in the entire transcriptome.

## Supplementary Material

btaf688_Supplementary_Data

## Data Availability

The data underlying this article are available in Gene Expression Omnibus at https://www.ncbi.nlm.nih.gov/geo/, and can be accessed with GSE274641.
