# Supplementary material for: ASTRO: Automated Spatial-Transcriptome whole RNA Output
Source: Bioinformatics. 2026 Jan 6;42(2):btaf688. doi: 10.1093/bioinformatics/btaf688 (PMC12866646; doi:10.1093/bioinformatics/btaf688)
Supplement: btaf688_Supplementary_Data [file btaf688_supplementary_data.zip › Supplementary File 1/Supplementary File 1/Spatial_mapping/the_number_of_gene_feature/ST-pipeline/MALT.pdf]

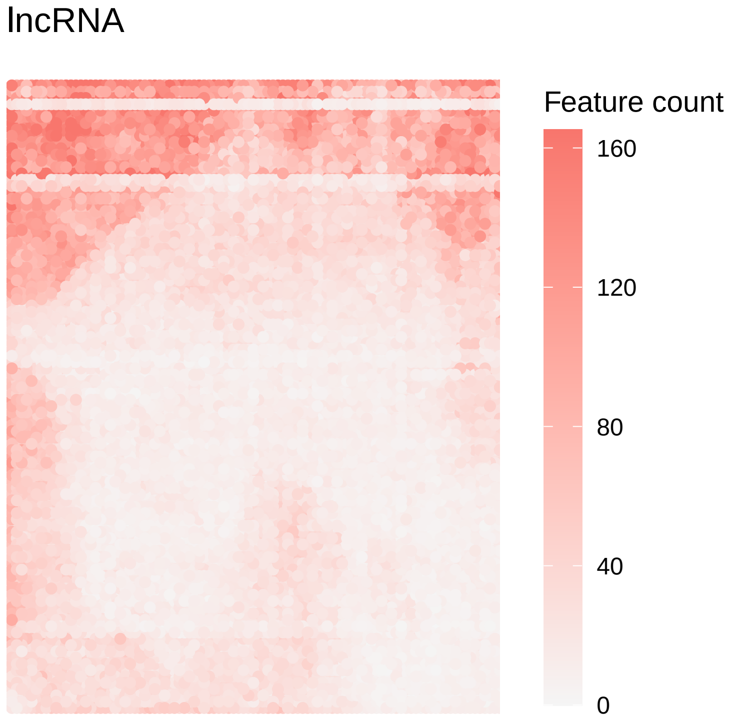
lncRNA
Feature count
160
120
80
40
0

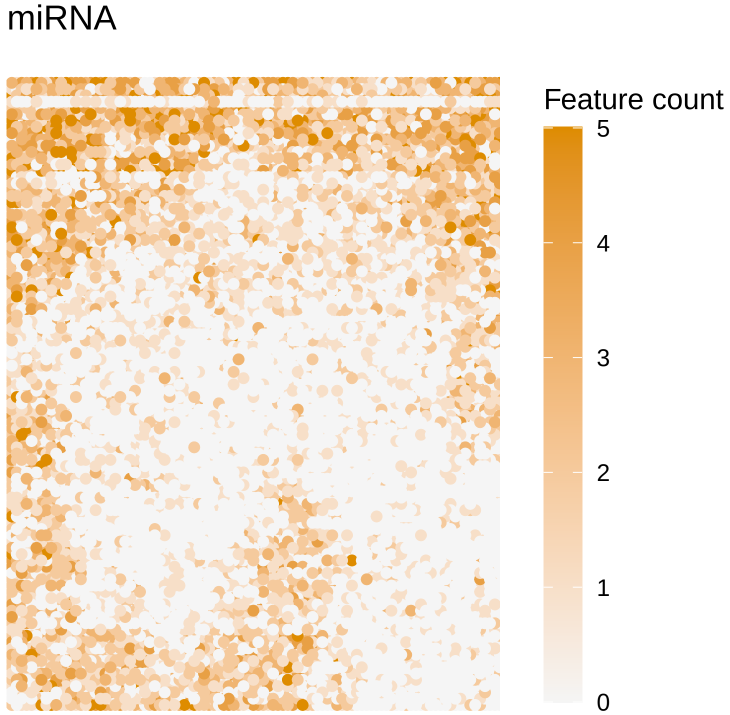
miRNA
Feature count
5
4
3
2
1
0

misc_RNA

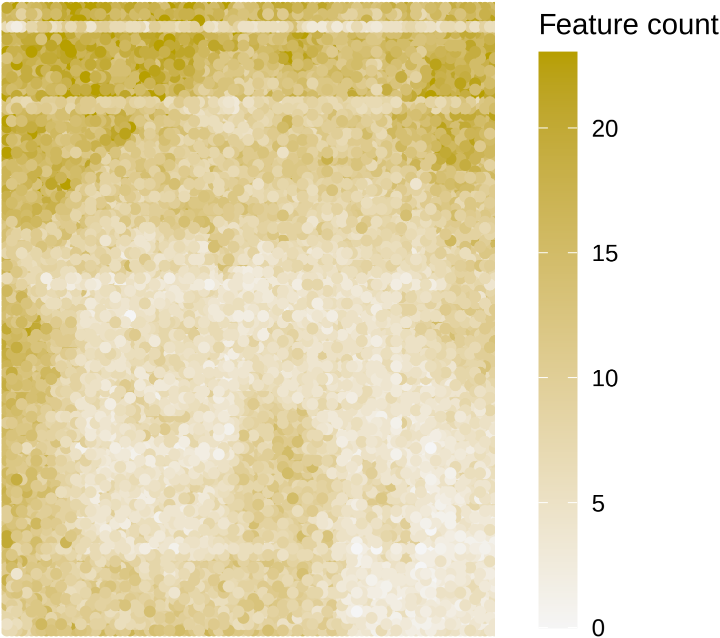

protein_coding

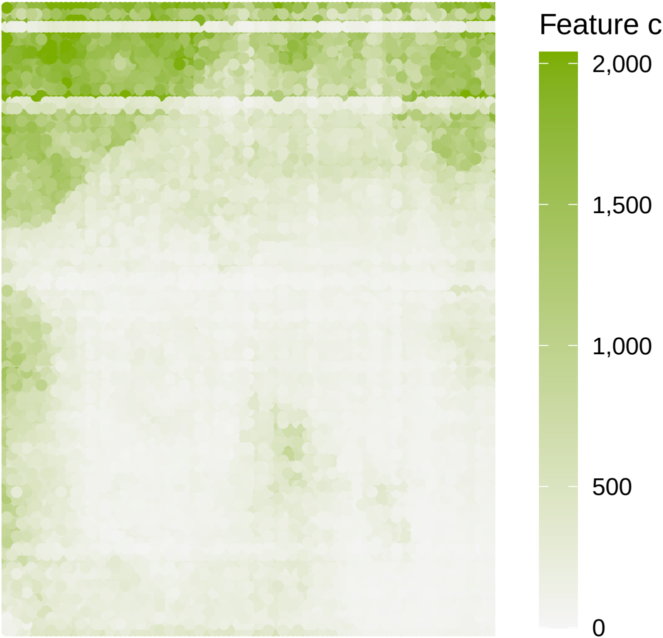

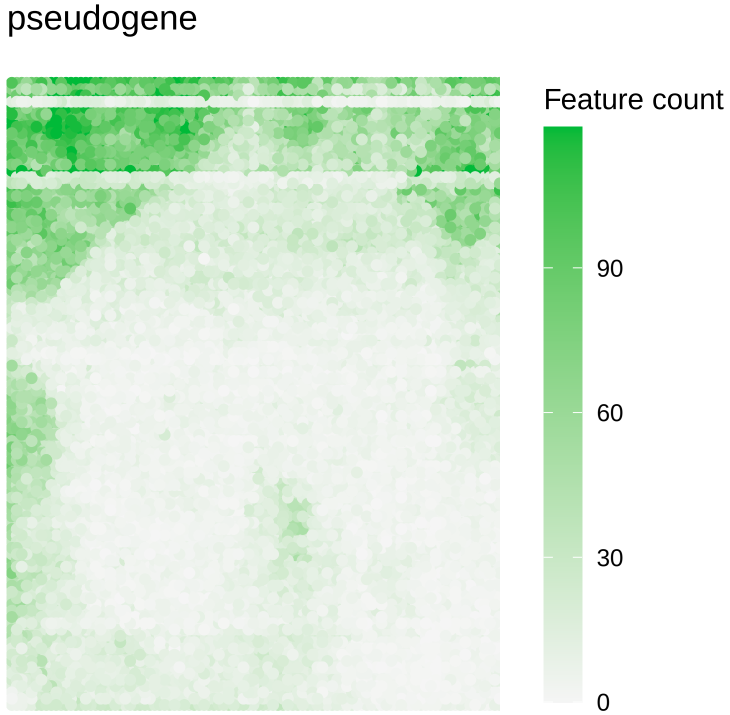
pseudogene
Feature count
90
60
30
0

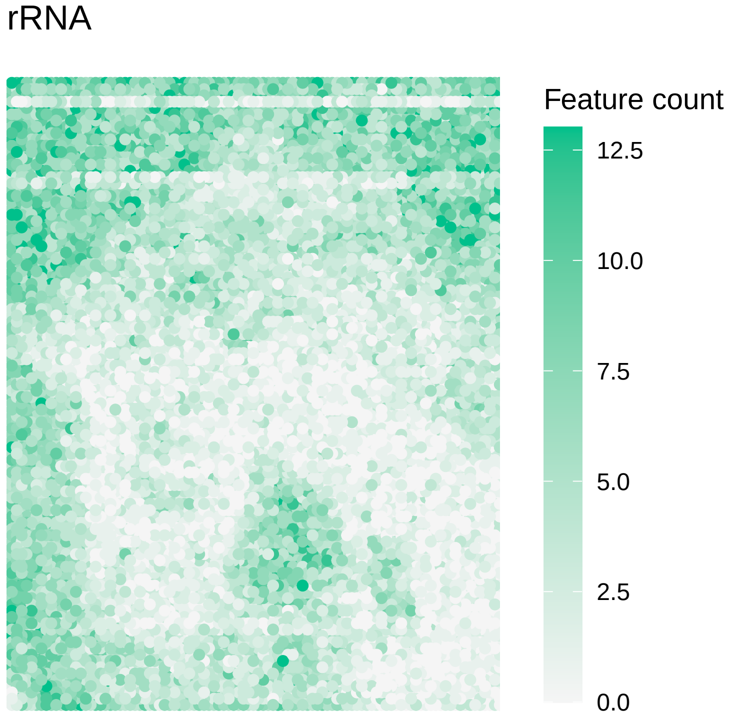

rRNA
Feature count
12.5
10.0
7.5
5.0
2.5
0.0

scaRNA

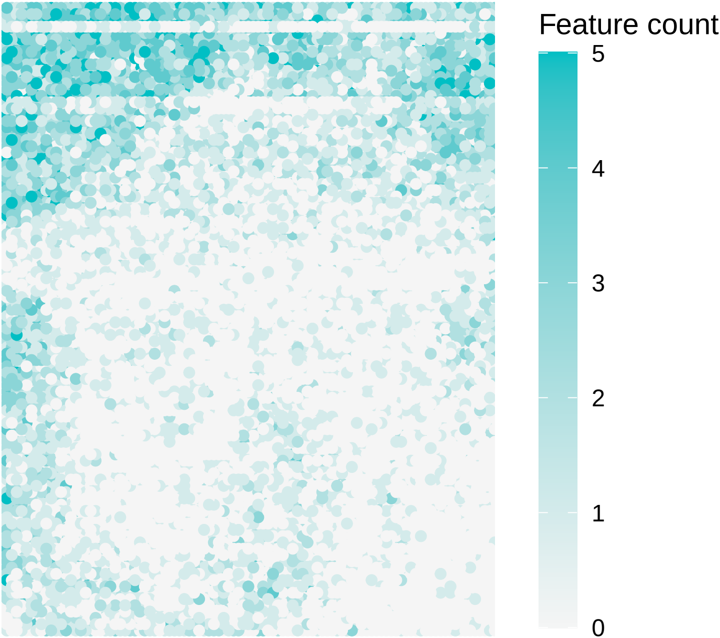

snRNA

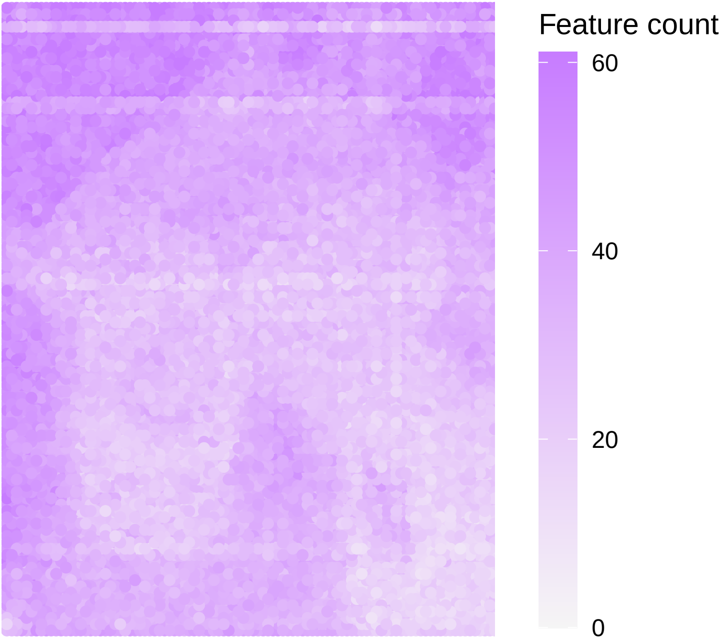

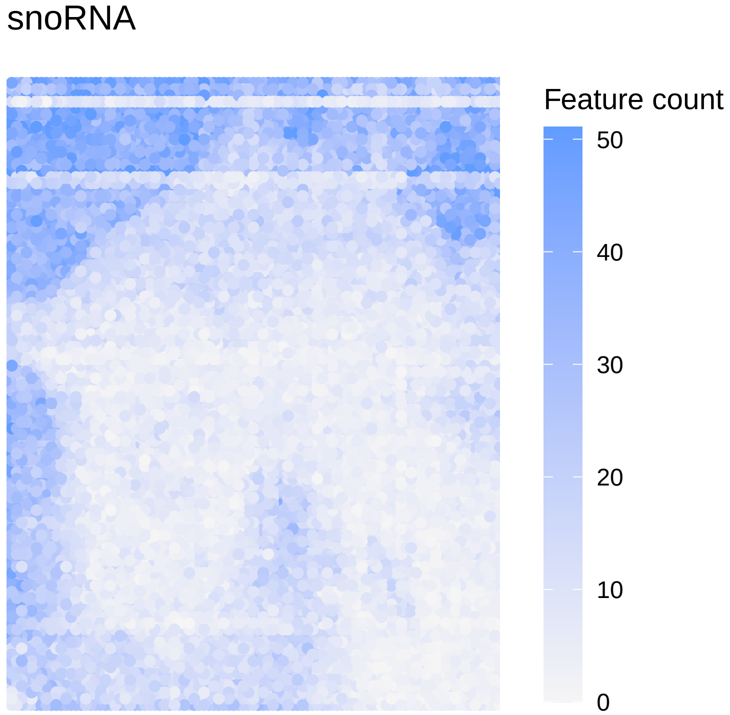
snoRNA
Feature count
50
40
30
20
10
0
