# Supplementary material for: ASTRO: Automated Spatial-Transcriptome whole RNA Output
Source: Bioinformatics. 2026 Jan 6;42(2):btaf688. doi: 10.1093/bioinformatics/btaf688 (PMC12866646; doi:10.1093/bioinformatics/btaf688)
Supplement: btaf688_Supplementary_Data [file btaf688_supplementary_data.zip › Supplementary Material.docx]

***
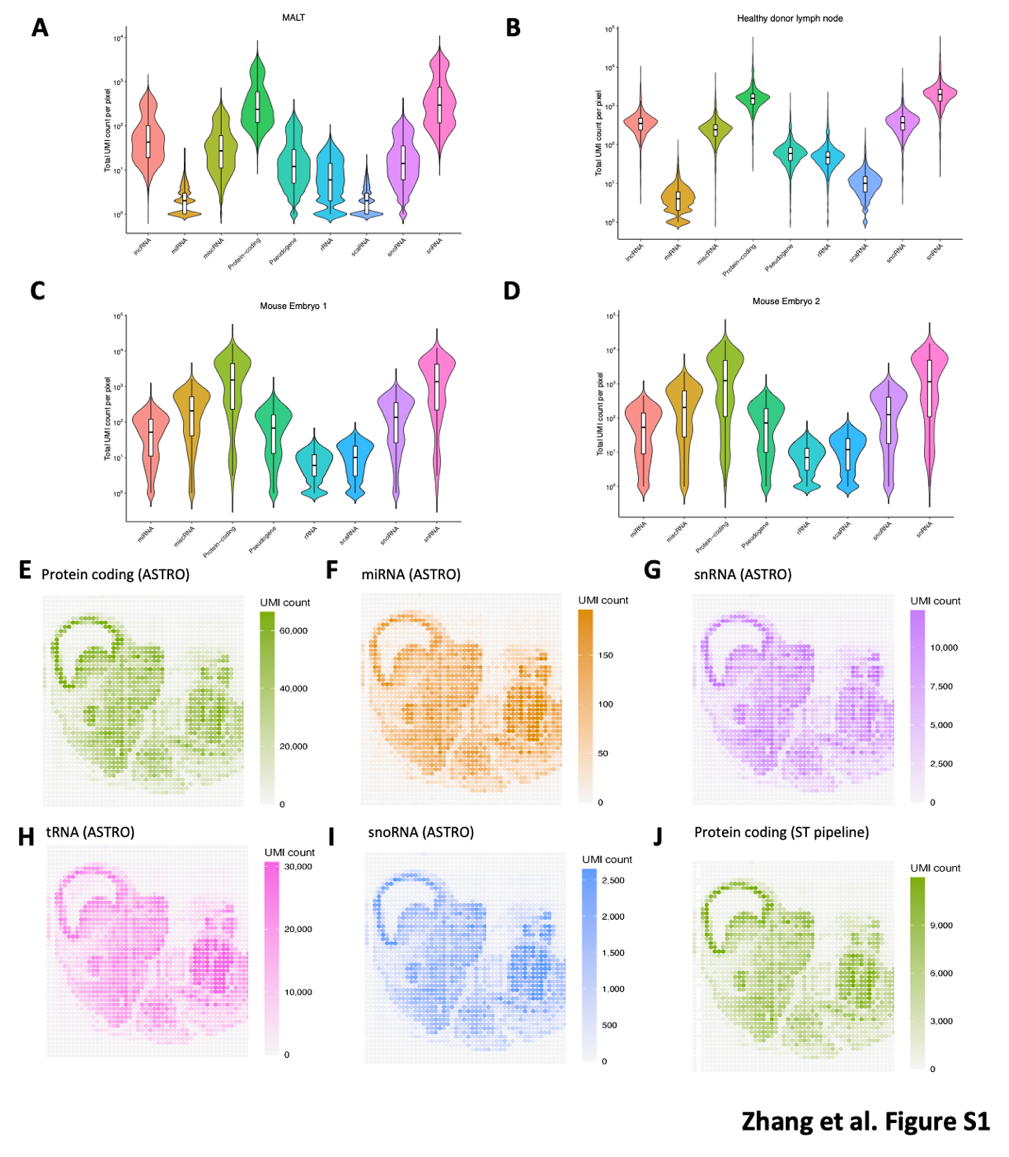
***

**Figure S1 (A-D)** Violin plots show the distribution of UMI per pixel as quantified by the ST-pipeline for the various RNA species, to compare with ASTRO results in **Figure 2**. (**E-I)** The spatial UMI count distributions of the indicated RNA types in sample “Mouse Embryo 1” are shown. (**J)** The UMI count distribution of protein coding genes in sample “Mouse Embryo 1” is shown spatially from the ST-pipeline result.

***
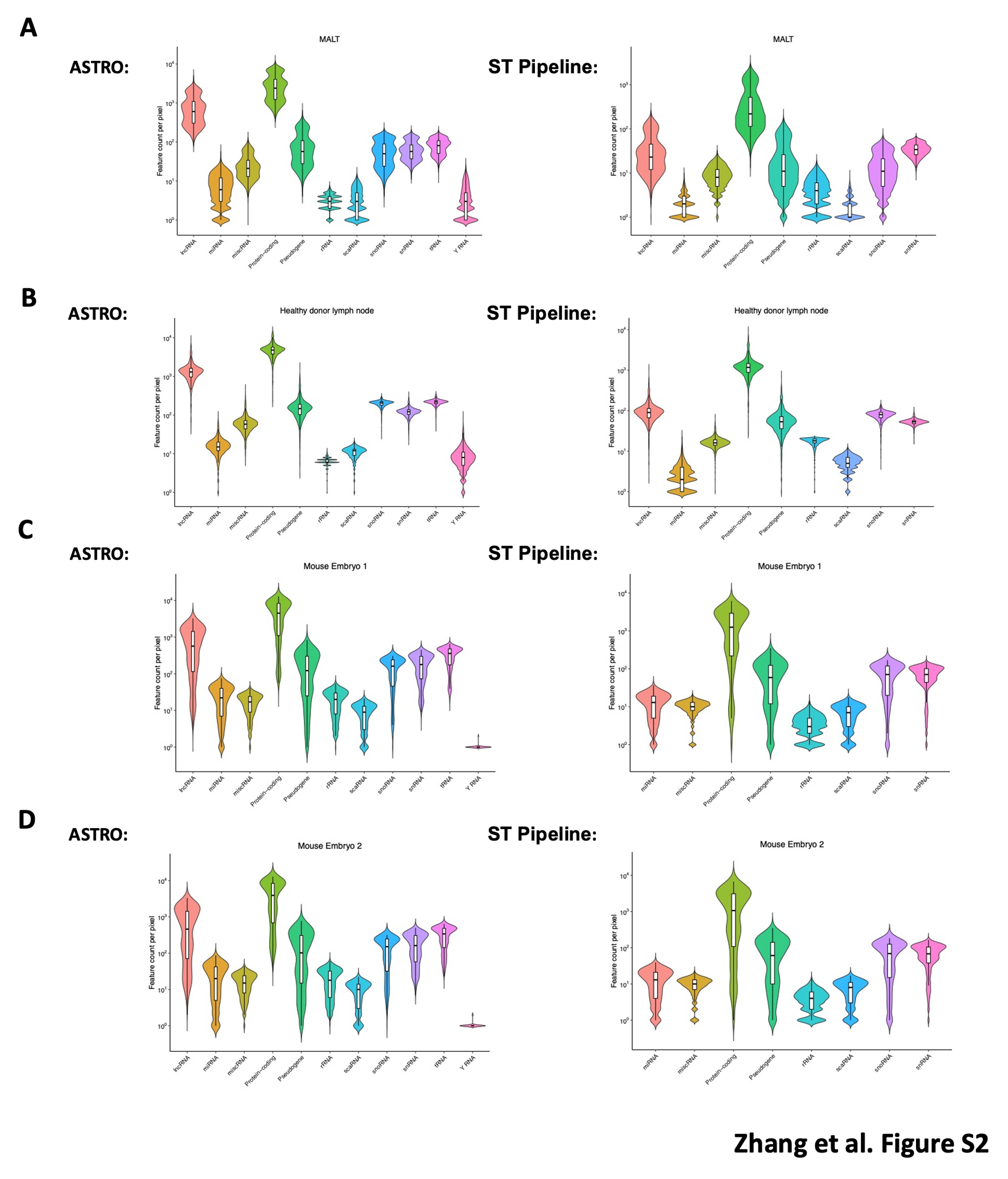
***

**Figure S2** Violin plots show the ability of ASTRO and ST-pipeline to detect various RNA species, with the y-axis indicating the number of gene features assigned to each species. Within each row, the left panel shows ASTRO results, and the right panel shows ST-pipeline results. Different rows correspond to different samples: **(A)** human MALT, **(B)** healthy-donor lymph node, **(C–D)** mouse embryo.

***
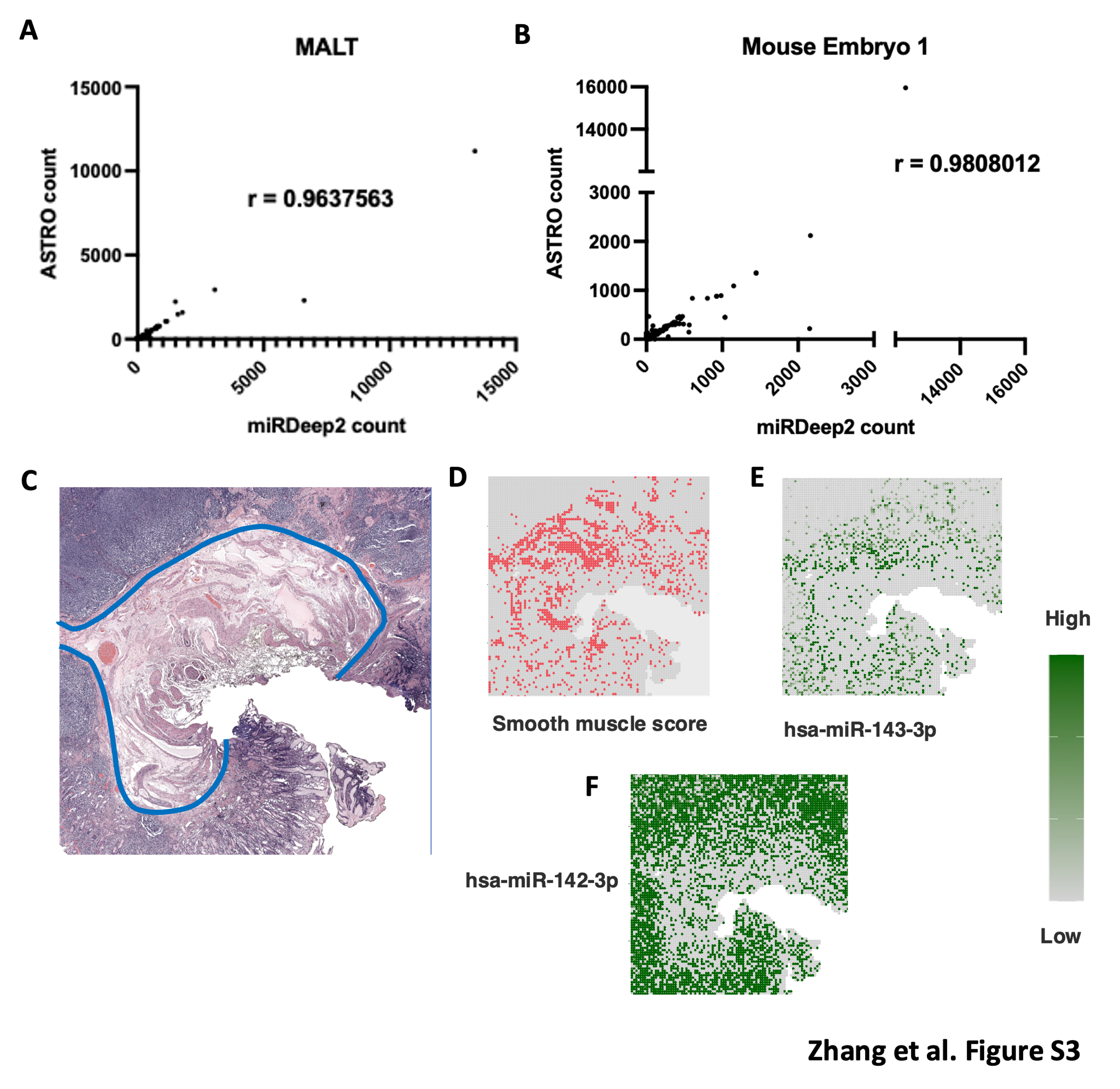
Details of Genomic Feature Validation***

**Figure S3 Spatial miRNA expression detected by ASTRO.** **(A–B)** Scatter plots assessing the correlation of miRNA UMI counts quantified by miRDeep2 and ASTRO are presented. Pearson correlation coefficients are annotated in each plot. The axes in **(B)** are displayed with a break for better visualization. **(C)** Histology image of the MALT sample with the smooth-muscle region outlined in blue. **(D)** Spatial distribution of spots identified as smooth muscle. **(E)** Spatial distribution of hsa-miR-143-3p. **(F)** Spatial distribution of hsa-miR-142-3p.

ASTRO first identifies all reads mapped to a given feature. The pipeline then compares the read coverage in these regions to that of an extended background. Specifically, it counts the number of read start and end sites within an extended feature region (by default, 5 bp upstream and downstream, Region 1) and compares these counts to those in a larger background region (an additional 5 bp upstream and downstream, Region 2). A feature is considered valid if its coverage is significantly enriched relative to the background. The statistical method employed is a two-sample Poisson intensity rate comparison (test_poisson_2indep in the statsmodels package) (Gu et al., 2008; Ng et al., 2007; Seabold & Perktold, 2010). The test compares the densities of 5' and 3' read ends in Regions 1 and 2. When performing test_poisson_2indep, the number of 5'/3' reads located in Region 1 is assigned to count1, and the length of Region 1 is assigned to exposure1. Likewise, the number of start/end sites in Region 2 is assigned to count2, and the length of Region 2 is assigned to exposure2. A read is counted in Region 1 only if both its 5' and 3' ends fall within Region 1. A feature is considered invalid if either the 5' or 3' read ends within Regions 1 and 2 fail to satisfy the following criteria: (1) the density in Region 1 is at least *x*-fold higher than in Region 2, and (2) the *p*-value is below *y*. By default, *x* = 2 and *y* =0.05.

***Details of whole RNA reference establishment***

To include annotations for different RNA species, we collected annotation data from several databases. Because GENCODE is one of the most widely used resources and includes various RNA species (Mudge et al., 2025), we used it as the primary source for our annotation, specifically v45 for GRCh38 human genome assembly and vM34 for mm39 mouse genome assembly. To enable exon–intron differentiation during downstream feature assignment, we retain only exon and transcript annotations, and label transcript records as intron. However, for one of the most studied non-coding RNA species, miRNA, GENCODE does not include isoform information (a single pre-miRNA produces two mature microRNAs, denoted as the 5p and 3p isoforms), and miRBase is therefore a better resource. Hence, we utilized miRBase (v22) for both human and mouse (Kozomara & Griffiths-Jones, 2011, 2014). tRNA annotation with codon and amino acid information is also missing from the GENCODE database. Therefore, we collected tRNA records from the database (Chan & Lowe, 2016). (GtRNAdb does not provide an explicit version number, but the datasets we used are the GRCh38 build for human and the mm39 build for mouse, downloaded on 2024-05-01 and 2024-08-11, respectively.)

In addition, piRNA is another non-coding RNA species that is not annotated in the GENCODE database despite many studies. Therefore, we selected the piRBase database to obtain piRNA annotations (Wang et al., 2019). Also, some other non-coding RNA species, such as vault RNA and Y RNA, are not included in the GENCODE database. As they do not have specialized databases, we extracted these annotations from the RNAcentral database (Sweeney et al., 2021). Both human and mouse GFF3 files used RNAcentral “Release 25”, and the release note file is included in the GitHub repository.

Before combining all datasets, because piRBase and miRBase only provide mouse data for the mm10 genome assembly, we used UCSC liftOver to convert the genomic coordinates in those annotations from mm10 to mm39 (Hinrichs et al., 2006). We then combined all the entries from the above databases into a single gene annotation file.

During the combination, if several entries are on the same chromosome and strand, with identical start and end genomic coordinates, those entries will be collapsed into one new record. The collapsed record was renamed to the concatenation of the pre-collapsed names, using double dashes (e.g., “gene1--gene2--…--geneN”). Additionally, miRNA records in the GENCODE annotation were removed to avoid unnecessary duplication during combination. Also, for genes with exon annotation records, the transcript records of them are labeled as intron annotation records. Finally, to facilitate further analysis, the ninth column of the new GTF files contains a string consisting of the gene name, gene type, and exon/intron label, joined by double underscores (__).

Finally, after the curation, the curated genome annotation file includes those biotypes: protein coding genes, lncRNA, miRNA, mitochondrial rRNA, mitochondrial tRNA, piRNA, pseudogene, ribozyme, sRNA, TEC, immunoglobulin gene, T-cell receptor gene, vault RNA, and Y RNA. And the summary statistics of major RNA biotypes is shown in **Supplementary Table 1-2**.

***Comparison between miRDeep2 and ASTRO in miRNA expression quantification***

miRDeep2 is a widely used tool for miRNA expression quantification and is considered a gold standard in the field. Because miRDeep2 and other commonly used pipelines (e.g., nf-core/smrnaseq and miRge3.0) are not designed for spatial transcriptomics data, we aggregated reads across spots to generate pseudo-bulk libraries for a fair comparison. In both human and mouse samples, the miRNA expression estimates produced by ASTRO were highly correlated with those from miRDeep2 (v2.0.1.2) **(Figure S3 A-B**), with Pearson correlation coefficients of 0.9637563 (MALT sample) and 0.9808012 (mouse embryo sample), respectively. Given that miRDeep2 is designed for bulk small RNA-seq and ASTRO is designed for whole-transcriptome profiling of FFPE tissue sections, these high correlations further support the robustness of ASTRO.

***
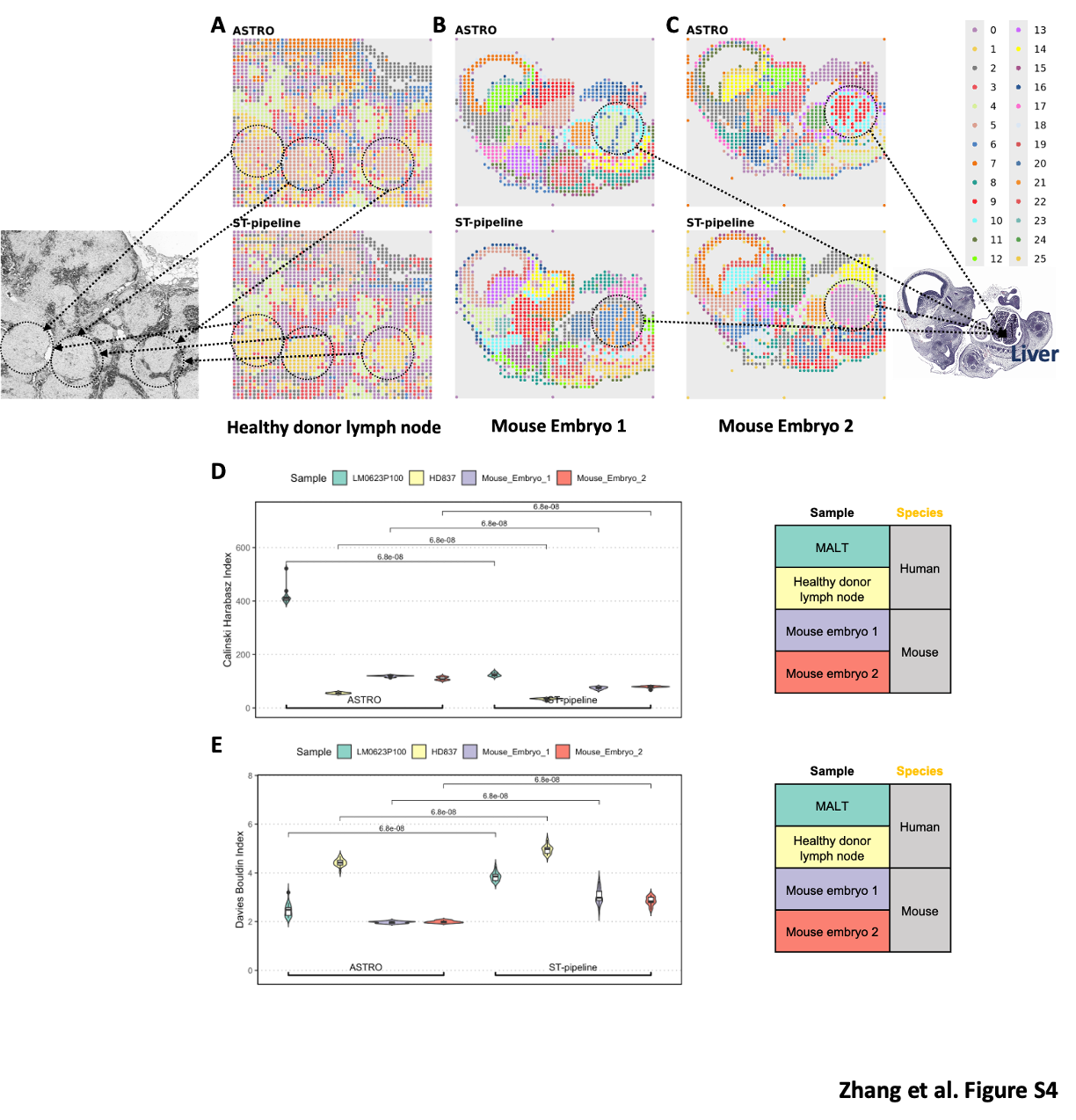
***

**Figure S4 Quantitative measurement of downstream analysis.** **(A)** Comparison of ASTRO-based clustering, and ST-pipeline-based clustering for the healthy donor lymph node sample. The black circles highlight subtle structures in the tissue. The corresponding histological image is shown on the left. **(B-C)** Comparison of ASTRO-based clustering, and ST-pipeline-based clustering in two mouse embryo samples. The black circles highlight the liver region. The corresponding histological image is shown on the right. **(D-E)** Four samples were analyzed by ASTRO and ST-pipeline, separately. The performance is shown as Calinski-Harabasz Index **(D)** Davies-Bouldin Index **(E)**, separately.

***Data Subsampling and Benchmarking of Pipelines in Patho-DBiT datasets***

The original expression data matrices were randomly downsampled to 50% of the total reads, with subsampling repeated 20 times for each sample. Downstream gene expression analysis was conducted using the Seurat V5 pipeline (Hao et al., 2023). SCTransform (Choudhary & Satija, 2022; Hafemeister & Satija, 2019) was employed to normalize gene expression for each pixel, followed by principal component analysis (PCA) via the RunPCA function, retaining the top 50 principal components for further analysis. A K-nearest neighbor graph was then constructed using the FindNeighbors function, based on Euclidean distance in the PCA space, and pixel clustering was performed with the FindClusters function. To facilitate comparisons, custom script was deployed to fix the number of clusters at 15 in each round for both ASTRO-derived and ST-pipeline-derived data. Clustering performance was assessed using the Silhouette score, Calinski–Harabasz index, and Davies–Bouldin index **(Figure 3E, Figure S4 D-E)**. These metrics, which quantify intra-cluster cohesion and inter-cluster separation, are widely used to evaluate the quality of single-cell expression data clustering (Buitinck et al., 2013; Jiang et al., 2018; Leng et al., 2022; Møller & Madsen, 2023; Yu et al., 2022).

***Spatial presentation of Patho-DBiT datasets***

For MALT sample, the analysis was conducted using the Seurat V5 pipeline (Hao et al., 2023). SCTransform (Choudhary & Satija, 2022; Hafemeister & Satija, 2019) was employed to normalize gene expression for each pixel, followed by PCA via the RunPCA function. Elbow plots were used to determine the number of principal components to retain: 10 for ASTRO-based data and 6 for ST-pipeline-based data. A K-nearest neighbor graph was then constructed using the FindNeighbors function, based on Euclidean distance in the PCA space, and pixel clustering was performed with the FindClusters function. To facilitate comparisons, the resolution of clusters was set to 1.2 for both ASTRO- and ST-pipeline-derived data. The same procedure was applied to the healthy donor lymph node sample and two mouse embryo samples (**Figure S4 C-E**). For ASTRO-derived data, the number of retained principal components were 20, 30, and 30 for the three samples, respectively, whereas for ST-pipeline-derived data the retained PCs were also 20, 30, and 30, respectively. The resolutions of clusters were matched between pipelines within each sample and set to 1, 3, and 3 for the three samples, respectively.


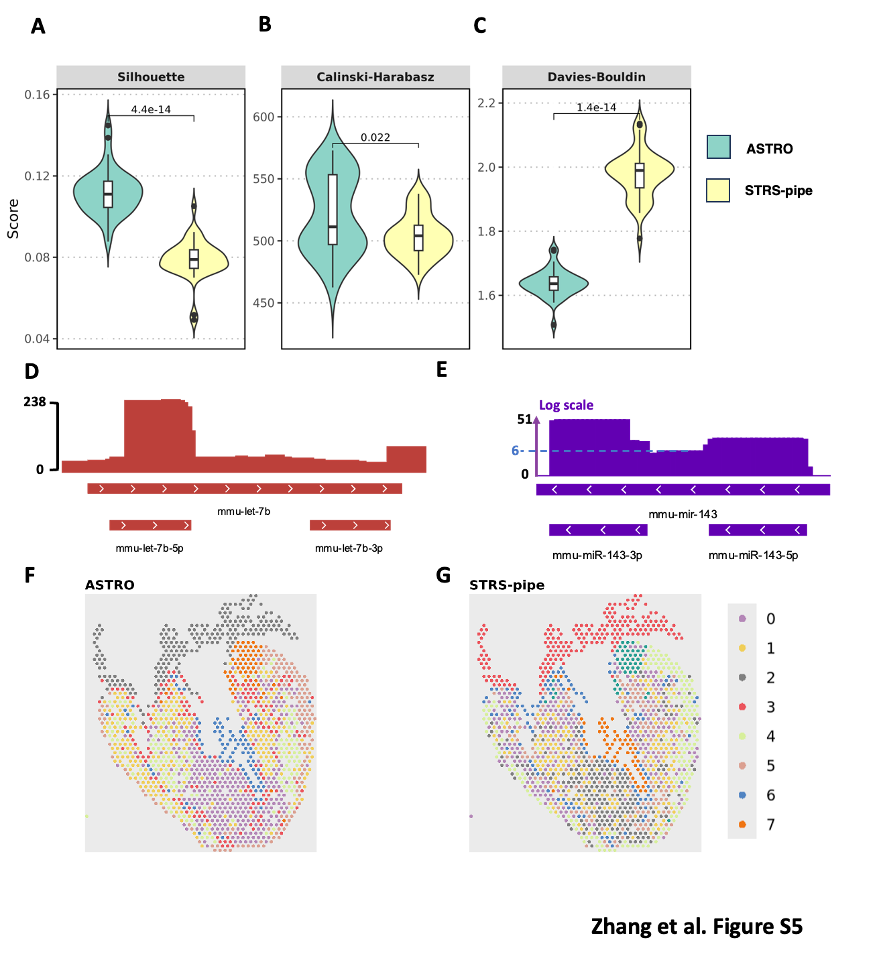


**Figure S5 Benchmarking ASTRO in a STRS dataset. (A–C)** Evaluation of downstream clustering performance for ASTRO in comparison to the custom STRS pipeline (referred to as STRS-pipe). The dataset used was GSM6034864. In each replicate, reads were randomly downsampled to 50%, spots were clustered, and the following metrics were computed: **(A)** Silhouette score, **(B)** Calinski–Harabasz index, and **(C)** Davies–Bouldin index. This downsampling was repeated 40 times to enable a robust comparison. **(D)** Read coverage around the mmu-let-7b locus shows a pattern consistent with the miRNA annotation; ASTRO detected different processing stages of this miRNA. **(E)** Read coverage around the mmu-mir-143 locus shows a pattern consistent with the miRNA annotation; ASTRO detected different processing stages of this miRNA. For better visualization, this plot is shown on a logarithmic scale. **(F-G)** Comparison of ASTRO-based clustering and STRS-pipe-based clustering for this sample.

***Applicability of*** ***other spatial whole transcriptomics datasets***

To address the question whether the applicability of ASTRO to other spatial whole transcriptomics datasets, we directly analyzed publicly available STRS data from a mouse heart sample (GSM6034864). The author-provided count matrix generated by one of the study’s custom pipelines (referred to as STRS-pipe), which uses STAR for read mapping, was used as the baseline for downstream clustering. Based on the elbow plot, we retained the top 15 principal components for this analysis. ASTRO outperformed the baseline on all three clustering metrics: the Silhouette score, Calinski–Harabasz index, and Davies–Bouldin index (**Figure S5 A-C**).

Additionally, we focus on the ability of ASTRO to detect non-coding RNA expression in STRS dataset. In the STRS study, the authors split the data by spatial location and applied miRge3.0 to each subset to obtain microRNA distributions. This is a reasonable approach for quantifying microRNA expression; however, because miRge3.0 is designed for small RNA-seq data rather than whole transcriptome data, it targets mature miRNAs and does not consider different processing stages of miRNAs. In contrast, ASTRO captures different stages of miRNAs. For example, while the authors report expression of mmu-let-7b-5p and mmu-miR-143-3p, ASTRO additionally detects their precursor forms. This added layer of information may facilitate future studies of spatial regulation on miRNA biogenesis. (**Figure S5 D-E**). The spatial cluster plots were generated by the same workflow described in the “Data Subsampling and Benchmarking of Pipelines in Patho-DBiT datasets” section. For both datasets, we applied an identical clustering resolution (1). The number of retained PCs was set to 20 for both ASTRO-derived data and STRS-pipe-derived data (**Figure S5 F-G**).


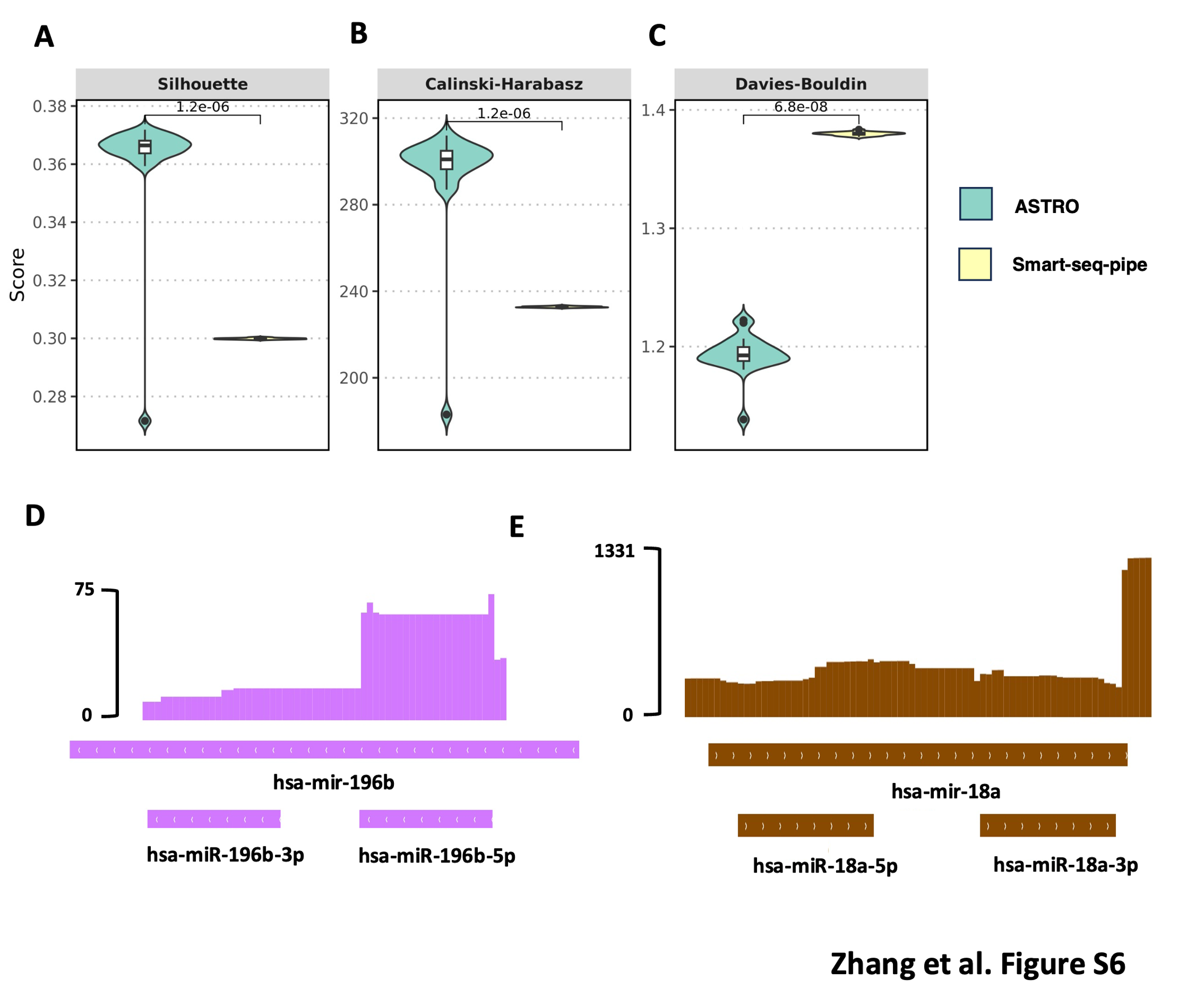


**Figure S6 Benchmarking ASTRO in a Smart-seq Total dataset. (A–C)** Evaluation of downstream clustering performance for ASTRO in comparison to the custom Smart-seq Total analysis pipeline (referred to as Smart-seq-pipe). The dataset used was GSE151334. In each replicate, reads were randomly downsampled to 50%, spots were clustered, and the following metrics were computed: **(A)** Silhouette score, **(B)** Calinski–Harabasz index, and **(C)** Davies–Bouldin index. This downsampling was repeated 20 times to enable a robust comparison. **(D)** Read coverage at the hsa-mir-196b locus shows a pattern consistent with the miRNA annotation; ASTRO detected this miRNA, whereas Smart-seq-pipe did not. **(E)** Read coverage around the hsa-mir-18a locus is inconsistent with the miRNA annotation, suggesting a likely false-positive call; this feature was reported by Smart-seq-pipe but filtered out by ASTRO.

***Applicability of ASTRO to Smart-seq Total***

To test the applicability of ASTRO in Smart-seq Total platform, we directly analyzed a publicly available dataset (GSE151334). For benchmarking, we used a human subset comprising three cell types (MCF7, HEK293T, and dermal fibroblast) and utilized the author-provided count matrix generated by the study’s custom pipeline as a baseline. Downstream clustering performance was evaluated using the Silhouette score, Calinski–Harabasz index, and Davies–Bouldin index, with the cluster number fixed at 3 to correspond to the three cell types. ASTRO outperformed the baseline on all three metrics. Moreover, ASTRO recovered a valid miRNA feature (hsa-miR-196b-3p) that is absent from the baseline matrix and excluded a likely false-positive feature (hsa-miR-18a) present in the baseline (**Figure S6**).


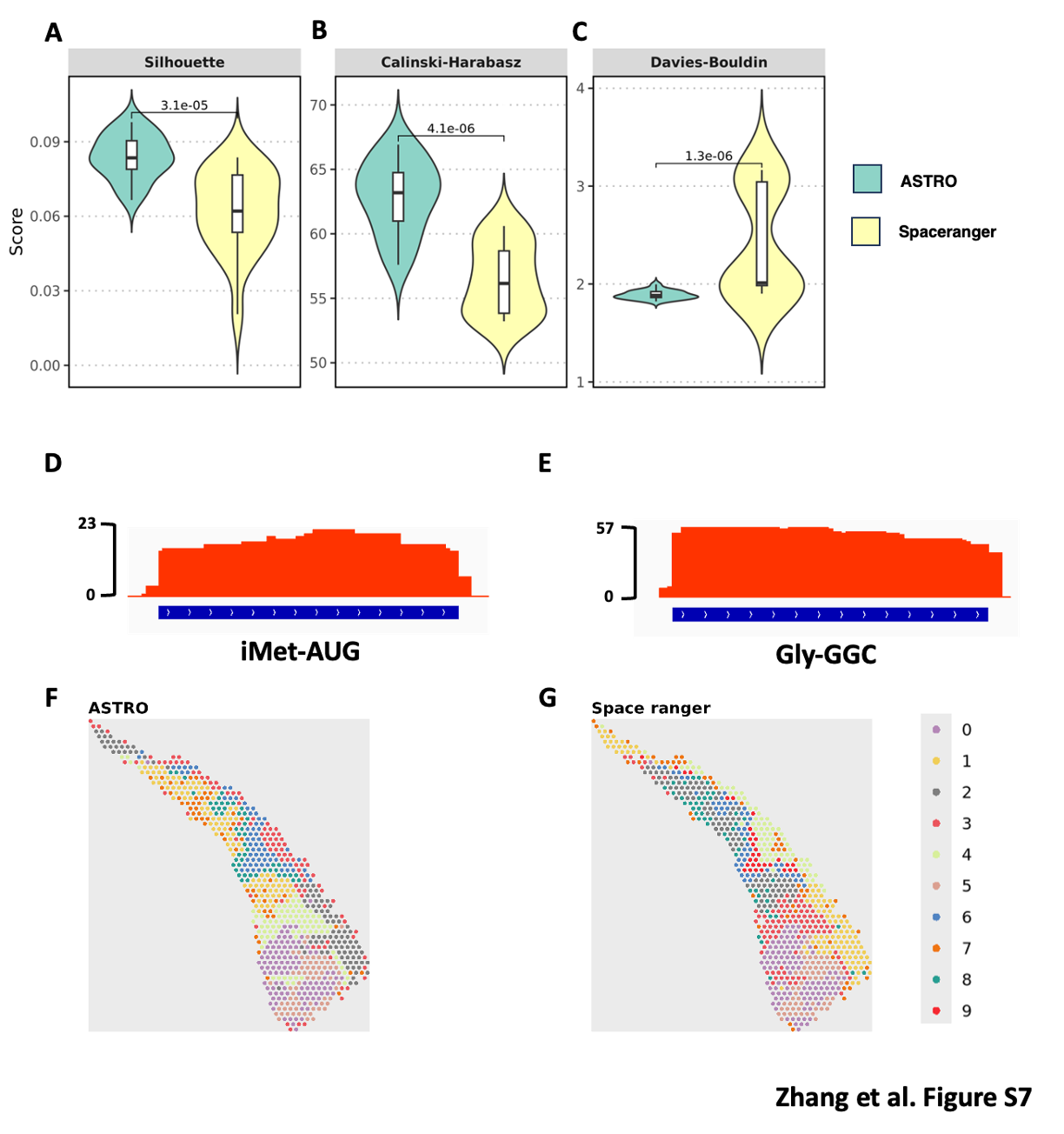


**Figure S7 Benchmarking ASTRO versus Space Ranger on a 10x Genomics Visium dataset. (A–C)** Evaluation of downstream clustering performance for ASTRO and Space Ranger. In each replicate, reads were randomly downsampled to 50%, spots were clustered, and the following metrics were computed: **(A)** Silhouette score, **(B)** Calinski–Harabasz index, and **(C)** Davies–Bouldin index. This downsampling was repeated 20 times to enable a robust comparison. **(D-E)** Read coverage around two tRNA loci shows a pattern consistent with the tRNA annotation; ASTRO detected those tRNAs, whereas Space Ranger did not. **(F-G)** Comparison of ASTRO-based clustering and Space Ranger-based clustering for this sample.

***Applicability of ASTRO to 10x Genomics Visium***

To assess the applicability of ASTRO to commercially available platforms and to benchmark ASTRO against Space Ranger, we downloaded a publicly available 10x Genomics Visium dataset (GSE151334). From the same GEO record, we obtained the author-provided count matrix generated by Space Ranger (v1.0.0). We evaluated downstream clustering performance by computing the Silhouette score, Calinski–Harabasz index, and Davies–Bouldin index on downsampled data. ASTRO-based analysis significantly outperformed Space-Ranger-based analysis on all three metrics **(Figure S7 A-C)**. Also, ASTRO excluded all miRNA features in this dataset, which is expected because the 10x Visium technology has a strong preference for poly(A)-tailed RNA molecules. However, ASTRO detects other non-coding RNAs that are missed by analyses based on the ST-pipeline. For example, two tRNA loci show enrichment of tRNA-derived reads: one corresponds to the initiator methionine tRNA (AUG codon), and the other corresponds to a glycine tRNA (GGC codon) **(Figure S7 D-E)**. The spatial cluster plots were generated by the same workflow described in the “Data Subsampling and Benchmarking of Pipelines in Patho-DBiT datasets” section. For both datasets, we applied an identical clustering resolution (1). The number of retained PCs was set to 15 for ASTRO-derived data and 20 for Spaceranger-derived data (**Figure S7 F-G**).


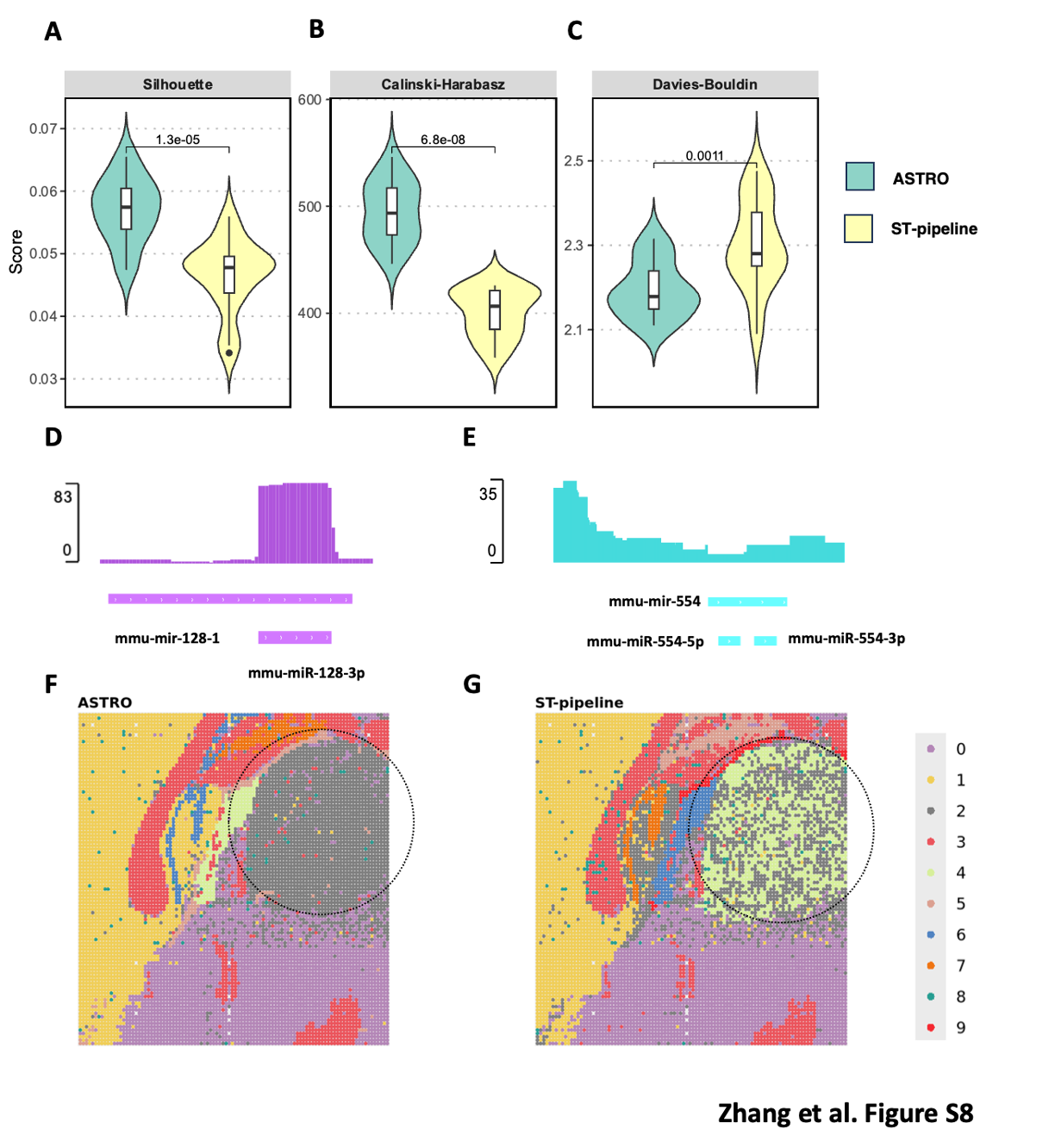


**Figure S8 Benchmarking ASTRO in a DBiT-seq dataset.** (**A–C**) Evaluation of downstream clustering performance for ASTRO. The baseline used for comparison was ST-pipeline generated matrix. In each replicate, reads were randomly downsampled to 50%, spots were clustered, and the following metrics were computed: (**A**) Silhouette score, (**B**) Calinski–Harabasz index, and (**C**) Davies–Bouldin index. This downsampling was repeated 20 times to enable a robust comparison. (**D**) Read coverage at the mmu-miR-128-3p locus shows a pattern consistent with the miRNA annotation; ASTRO detected this miRNA, whereas ST-pipeline did not. **(E)** Read coverage around the hsa-mir-554 locus is inconsistent with the miRNA annotation, suggesting a likely false-positive call; this feature was reported by ST-pipeline but filtered out by ASTRO. **(F-G)** Comparison of ASTRO-based clustering and ST-pipeline-based clustering for this sample. Black circles highlight regions where the two spatial clustering results differ, showing a lower noise level with ASTRO.

***Applicability of ASTRO to DBiT-seq***

To assess ASTRO’s applicability to the fresh-frozen version of the DBiT-seq platform, we generated an additional dataset from fresh-frozen mouse brain tissue by DBiT-seq. We analyzed this dataset with ASTRO and the ST-pipeline separately for benchmarking. We evaluated downstream clustering performance by computing the Silhouette score, Calinski–Harabasz index, and Davies–Bouldin index on downsampled data. ASTRO-based analysis significantly outperformed ST-pipeline-based analysis on all three metrics. Also, ASTRO recovered a valid miRNA feature (mmu-miR-128-3p) that is absent from the baseline matrix and excluded a likely false-positive feature (mmu-mir-554) present in the baseline. The spatial cluster plots were generated by the same workflow described in the “Data Subsampling and Benchmarking of Pipelines in Patho-DBiT datasets” section. For both datasets, we applied an identical clustering resolution (0.2). The number of retained PCs was set to 20 for both ASTRO-derived data and ST-pipeline-derived data (**Figure S8**).


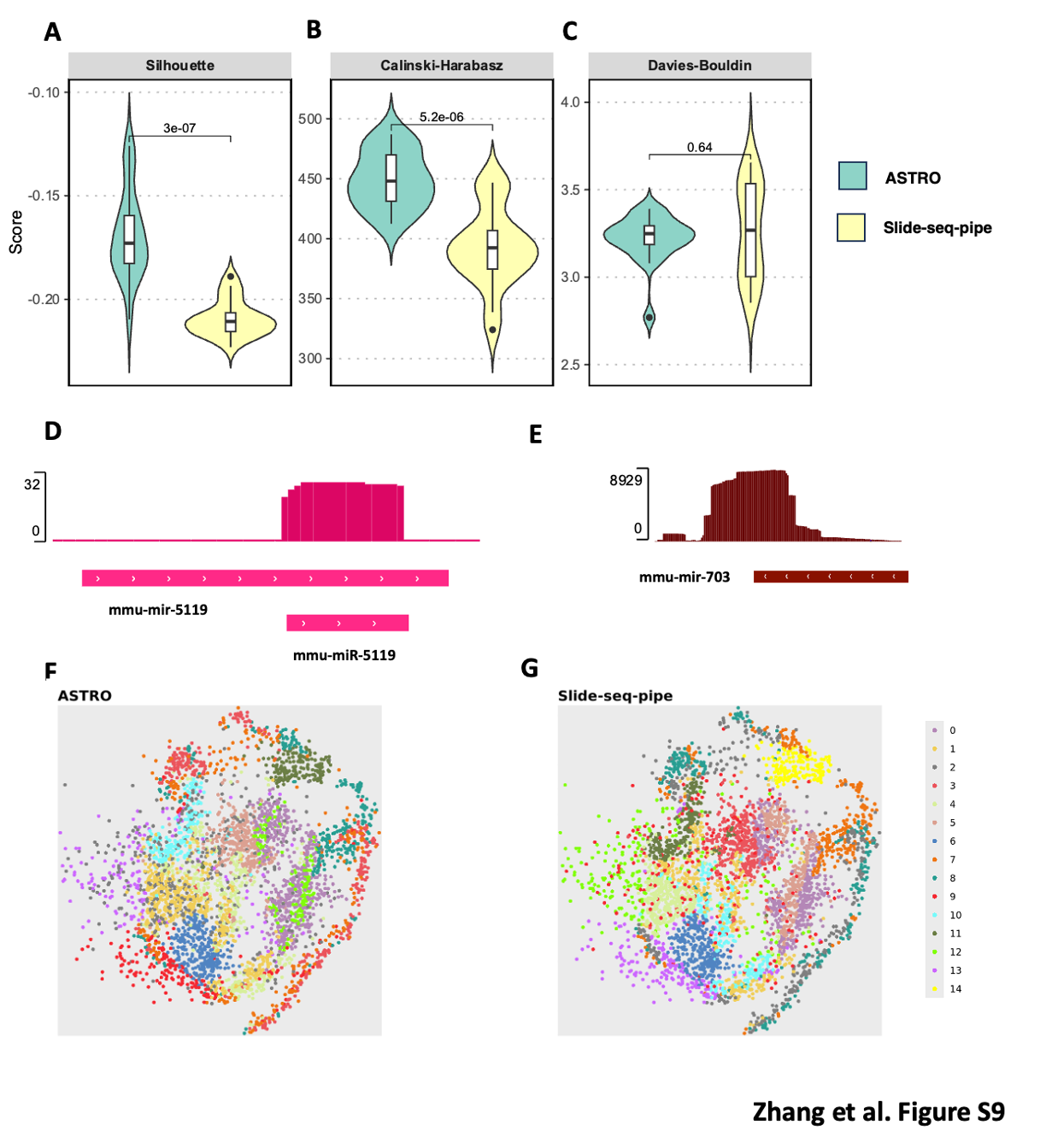


**Figure S9** **Applicability of ASTRO to Slide-seq.** **(A–C)** Evaluation of downstream clustering performance for ASTRO in comparison to the custom Slide-seq analysis pipeline (referred to as Slide-seq-pipe). The dataset used was GSE197353. In each replicate, reads were randomly downsampled to 50%, spots were clustered, and the following metrics were computed: **(A)** Silhouette score, **(B)** Calinski–Harabasz index, and **(C)** Davies–Bouldin index. This downsampling was repeated 20 times to enable a robust comparison. **(D)** Read coverage at the mmu-mir-5119 locus shows a pattern consistent with the miRNA annotation; ASTRO detected this miRNA, whereas Slide-seq-pipe did not. **(E)** Read coverage around the mmu-mir-703 locus is inconsistent with the miRNA annotation, suggesting a likely false-positive call; this feature was reported by Slide-seq-pipe but filtered out by ASTRO. **(F)** Comparison of ASTRO-based clustering and Slide-seq-pipe-based clustering for this sample.

***Applicability of ASTRO to Slide-seq***

To test the applicability of ASTRO in Smart-seq Total platform, we directly analyzed a publicly available dataset (GSE197353). For benchmarking, we used the sample “E8.5_embryo_puck_201104_07” and utilized the author-provided count matrix generated by the study’s custom pipeline as a baseline. Downstream clustering performance was evaluated using the Silhouette score, Calinski–Harabasz index, and Davies–Bouldin index. ASTRO outperformed the baseline on the Silhouette score and Calinski–Harabasz index. For the Davies–Bouldin index (lower is better), ASTRO showed a slightly lower mean, but the difference was not significant. Moreover, ASTRO recovered a valid miRNA feature (mmu-miR-5119), which has been previously reported in mouse embryos (Nagy et al., 2025) but was absent from the baseline matrix. Furthermore, ASTRO excluded a likely false-positive feature (mmu-mir-703) present in the baseline The spatial cluster plots were generated by the same workflow described in the “Data Subsampling and Benchmarking of Pipelines in Patho-DBiT datasets” section. For both datasets, we applied an identical clustering resolution (1.0) and number of retained PCs (20) for ASTRO-derived data and Slide-seq-pipe -derived data (**Figure S9**).

| **RNA Biotypes** | **GRCh38** | **mm39** |
| --- | --- | --- |
| protein coding | 440981 | 282368 |
| lncRNA | 181979 | 75021 |
| piRNA | 113747 | 103771 |
| miRNA | 4800 | 3327 |
| snRNA | 1901 | 1381 |
| snoRNA | 942 | 1507 |
| tRNA | 432 | 407 |
| Y RNA | 212 | 2 |
| rRNA | 47 | 354 |
| Other | 43476 | 30619 |

**Supplementary Table 1**

Number of major RNA biotypes in feature level

(considering exon and intron difference)

| **RNA Biotypes** | **GRCh38** | **mm39** |
| --- | --- | --- |
| protein coding | 19977 | 21570 |
| lncRNA | 19344 | 11882 |
| piRNA | 70078 | 80558 |
| miRNA | 4564 | 3182 |
| snRNA | 1837 | 1381 |
| snoRNA | 792 | 1484 |
| tRNA | 432 | 407 |
| Y RNA | 212 | 2 |
| rRNA | 40 | 354 |
| Other | 17919 | 18025 |

**Supplementary Table 2**

Number of major RNA biotypes in gene level

| **Sample** | **Species** | **Record No.** | **CPU No.** | **Runtime** | **Memory usage** |
| --- | --- | --- | --- | --- | --- |
| MALT | Human | 683247031 | 16 | 08:06:53 | 34.3GB |
| Lymph node (Ctrl) | Human | 268930100 | 16 | 03:29:20 | 34.3GB |
| Embryo | Mouse | 300680325 | 16 | 04:42:00 | 26.8GB |
| Embryo | Mouse | 321740206 | 16 | 04:46:03 | 26.8GB |

**Supplementary Table 3**

Time and memory requirements of ASTRO across different datasets

| Technology | Tissue Type | in situ  poly(A) | Data source | Resolution | Commercialization | Baseline Pipeline |
| --- | --- | --- | --- | --- | --- | --- |
| Patho-DBiT | FFPE | Yes | GSE274641 | Spatial | No | ST-pipeline |
| DBiT | Fresh | No | Unpublished | Spatial | Yes | ST-pipeline |
| Smart-seq  -Total | Fresh | Yes | GSE151334 | Single-cell | Yes | Smart -seq Custom Pipeline |
| 10x Genomics Visium | Fresh | No | GSE144240 | Spatial | Yes | Space Ranger |
| STRS | Fresh | Yes | GSE200481 | Spatial | No | STRS Custom pipeline |
| Slide-seq | Fresh | No | GSE197353 | Spatial | Yes | Slide-seq Custom pipeline |

**Supplementary Table 4**

Comparisons of technologies involved in this dataset

***References:***

Buitinck, L., Louppe, G., Blondel, M., Pedregosa, F., Müller, A. C., Grisel, O., Niculae, V., Prettenhofer, P., Gramfort, A., Grobler, J., Layton, R., Vanderplas, J., Joly, A., Holt, B., & Varoquaux, G. (2013). API design for machine learning software: experiences from the scikit-learn project. https://arxiv.org/abs/1309.0238v1

Chan, P. P., & Lowe, T. M. (2016). GtRNAdb 2.0: an expanded database of transfer RNA genes identified in complete and draft genomes. Nucleic Acids Research, 44(D1), D184–D189. https://doi.org/10.1093/NAR/GKV1309

Choudhary, S., & Satija, R. (2022). Comparison and evaluation of statistical error models for scRNA-seq. Genome Biology, 23(1), 1–20. https://doi.org/10.1186/S13059-021-02584-9/FIGURES/4

Gu, K., Ng, H. K. T., Man, L. T., & Schucany, W. R. (2008). Testing the ratio of two poisson rates. Biometrical Journal. Biometrische Zeitschrift, 50(2), 283–298. https://doi.org/10.1002/BIMJ.200710403

Hafemeister, C., & Satija, R. (2019). Normalization and variance stabilization of single-cell RNA-seq data using regularized negative binomial regression. Genome Biology, 20(1), 1–15. https://doi.org/10.1186/S13059-019-1874-1/FIGURES/6

Hao, Y., Stuart, T., Kowalski, M. H., Choudhary, S., Hoffman, P., Hartman, A., Srivastava, A., Molla, G., Madad, S., Fernandez-Granda, C., & Satija, R. (2023). Dictionary learning for integrative, multimodal and scalable single-cell analysis. Nature Biotechnology 2023 42:2, 42(2), 293–304. https://doi.org/10.1038/s41587-023-01767-y

Hinrichs, A. S., Karolchik, D., Baertsch, R., Barber, G. P., Bejerano, G., Clawson, H., Diekhans, M., Furey, T. S., Harte, R. A., Hsu, F., Hillman-Jackson, J., Kuhn, R. M., Pedersen, J. S., Pohl, A., Raney, B. J., Rosenbloom, K. R., Siepel, A., Smith, K. E., Sugnet, C. W., … Kent, W. J. (2006). The UCSC Genome Browser Database: update 2006. Nucleic Acids Research, 34(suppl_1), D590–D598. https://doi.org/10.1093/NAR/GKJ144

Jiang, H., Sohn, L. L., Huang, H., & Chen, L. (2018). Single cell clustering based on cell-pair differentiability correlation and variance analysis. Bioinformatics (Oxford, England), 34(21), 3684–3694. https://doi.org/10.1093/BIOINFORMATICS/BTY390

Kozomara, A., & Griffiths-Jones, S. (2011). miRBase: integrating microRNA annotation and deep-sequencing data. Nucleic Acids Research, 39(Database issue). https://doi.org/10.1093/NAR/GKQ1027

Kozomara, A., & Griffiths-Jones, S. (2014). MiRBase: Annotating high confidence microRNAs using deep sequencing data. Nucleic Acids Research, 42(D1). https://doi.org/10.1093/nar/gkt1181

Leng, D., Zheng, L., Wen, Y., Zhang, Y., Wu, L., Wang, J., Wang, M., Zhang, Z., He, S., & Bo, X. (2022). A benchmark study of deep learning-based multi-omics data fusion methods for cancer. Genome Biology, 23(1). https://doi.org/10.1186/S13059-022-02739-2

Møller, A. F., & Madsen, J. G. S. (2023). JOINTLY: interpretable joint clustering of single-cell transcriptomes. Nature Communications 2023 14:1, 14(1), 1–15. https://doi.org/10.1038/s41467-023-44279-8

Mudge, J. M., Carbonell-Sala, S., Diekhans, M., Gonzalez Martinez, J., Hunt, T., Jungreis, I., Lo eland, J. E., me Ar nan, C., Bar nes, I., Bennett, R., Berry, A., Bignell, A., Cerdán-Vélez, D., Coc hr an, K., Cor és, L. T., Da vidson, C., ah Donaldson, S., ata Dursun, C., Fatima, R., … dam ankish, A. F. (2025). GENCODE 2025: reference gene annotation for human and mouse. Nucleic Acids Research, 53(D1), D966–D975. https://doi.org/10.1093/NAR/GKAE1078

Nagy, B., Bognár, Z., Csabai, T. J., Fekete, N., Buzás, E. I., Kovács, Á. F., Szekeres-Barthó, J., & Pállinger, É. (2025). Effects of light exposure during IVF: transcriptomic analysis of murine embryos and embryo-derived EVs. Frontiers in Immunology, 16, 1429252. https://doi.org/10.3389/FIMMU.2025.1429252/FULL

Ng, H. K. T., Gu, K., & Tang, M. L. (2007). A comparative study of tests for the difference of two Poisson means. Computational Statistics & Data Analysis, 51(6), 3085–3099. https://doi.org/10.1016/J.CSDA.2006.02.004

Seabold, S., & Perktold, J. (2010). statsmodels: Econometric and statistical modeling with python. 9th Python in Science Conference.

Sweeney, B. A., Petrov, A. I., Ribas, C. E., Finn, R. D., Bateman, A., Szymanski, M., Karlowski, W. M., Seemann, S. E., Gorodkin, J., Cannone, J. J., Gutell, R. R., Kay, S., Marygold, S., Dos Santos, G., Frankish, A., Mudge, J. M., Barshir, R., Fishilevich, S., Chan, P. P., … Weinberg, Z. (2021). RNAcentral 2021: secondary structure integration, improved sequence search and new member databases. Nucleic Acids Research, 49(D1), D212–D220. https://doi.org/10.1093/NAR/GKAA921

Wang, J., Zhang, P., Lu, Y., Li, Y., Zheng, Y., Kan, Y., Chen, R., & He, S. (2019). piRBase: a comprehensive database of piRNA sequences. Nucleic Acids Research, 47(D1), D175–D180. https://doi.org/10.1093/NAR/GKY1043

Yu, L., Cao, Y., Yang, J. Y. H., & Yang, P. (2022). Benchmarking clustering algorithms on estimating the number of cell types from single-cell RNA-sequencing data. Genome Biology, 23(1), 1–21. https://doi.org/10.1186/S13059-022-02622-0/TABLES/1
