# Supplementary figures and images for: ASTRO: Automated Spatial-Transcriptome whole RNA Output
Source: Bioinformatics. 2026 Jan 6;42(2):btaf688. doi: 10.1093/bioinformatics/btaf688 (PMC12866646; doi:10.1093/bioinformatics/btaf688)

# Healthy donor lymph node

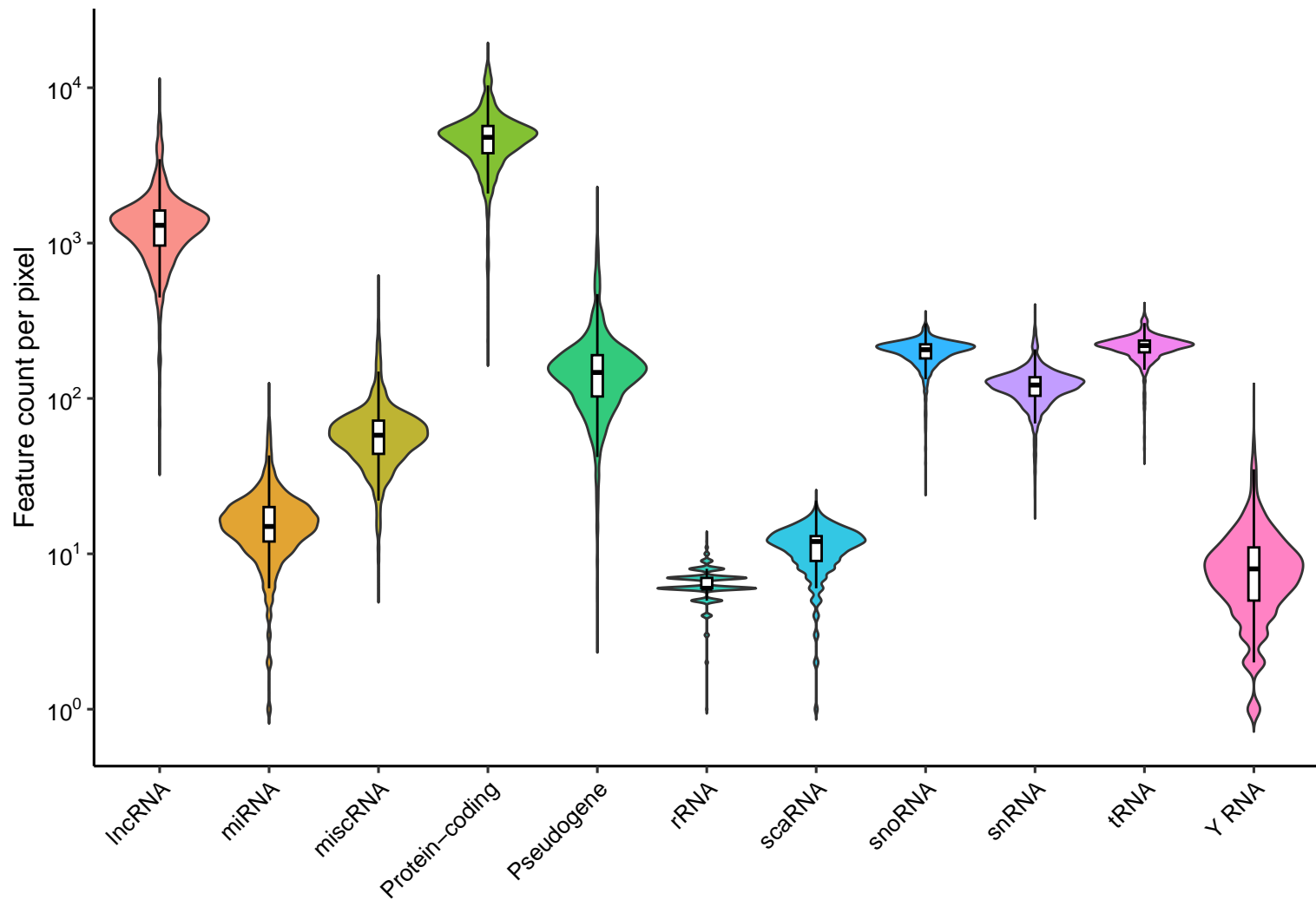

Supplement: btaf688_Supplementary_Data [file btaf688_supplementary_data.zip › Supplementary File 1/Supplementary File 1/the_number_of_gene_feature/ASTRO/Healthy donor lymph node.pdf]

# MALT

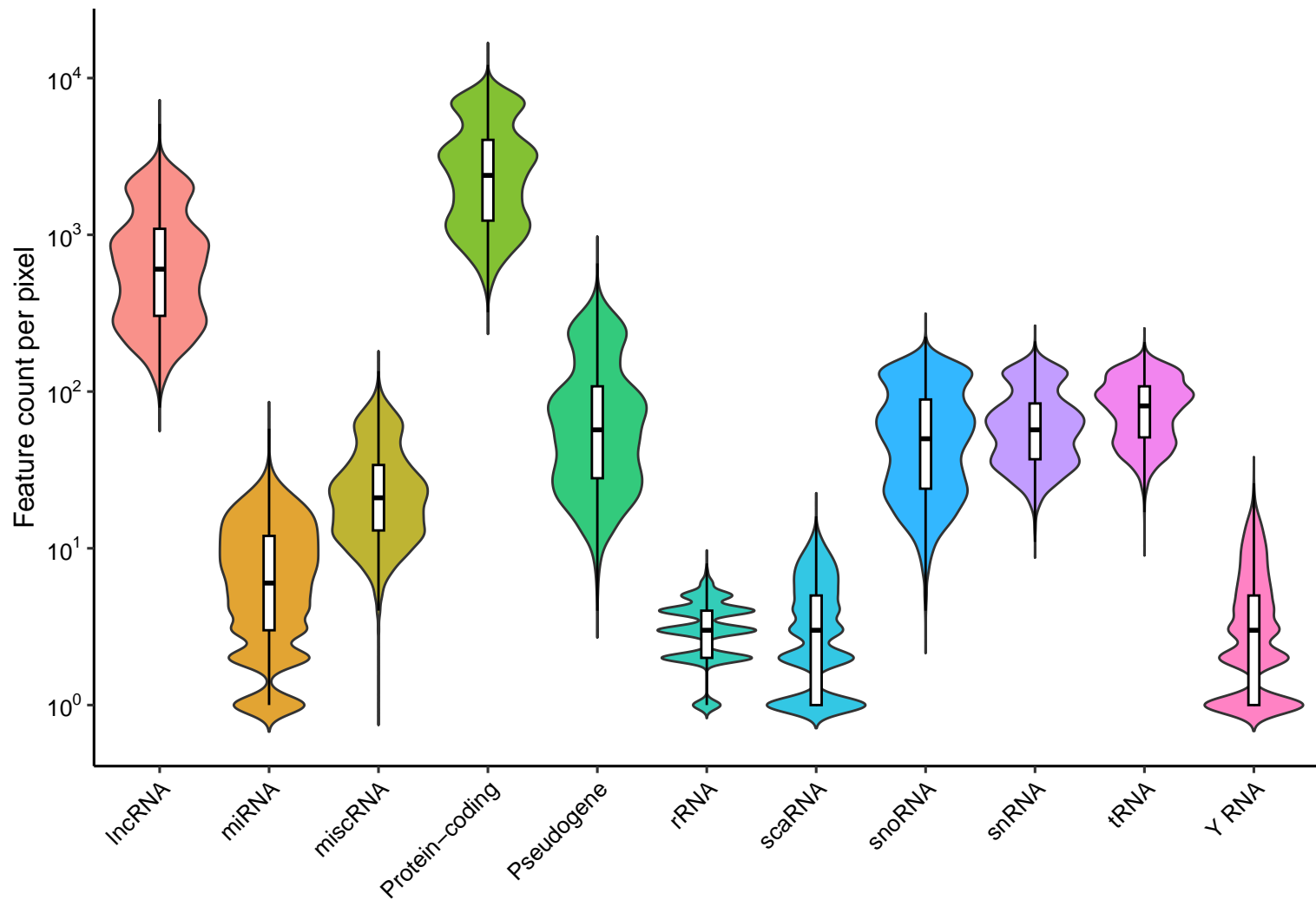

Supplement: btaf688_Supplementary_Data [file btaf688_supplementary_data.zip › Supplementary File 1/Supplementary File 1/the_number_of_gene_feature/ASTRO/MALT.pdf]

# Mouse Embryo 1

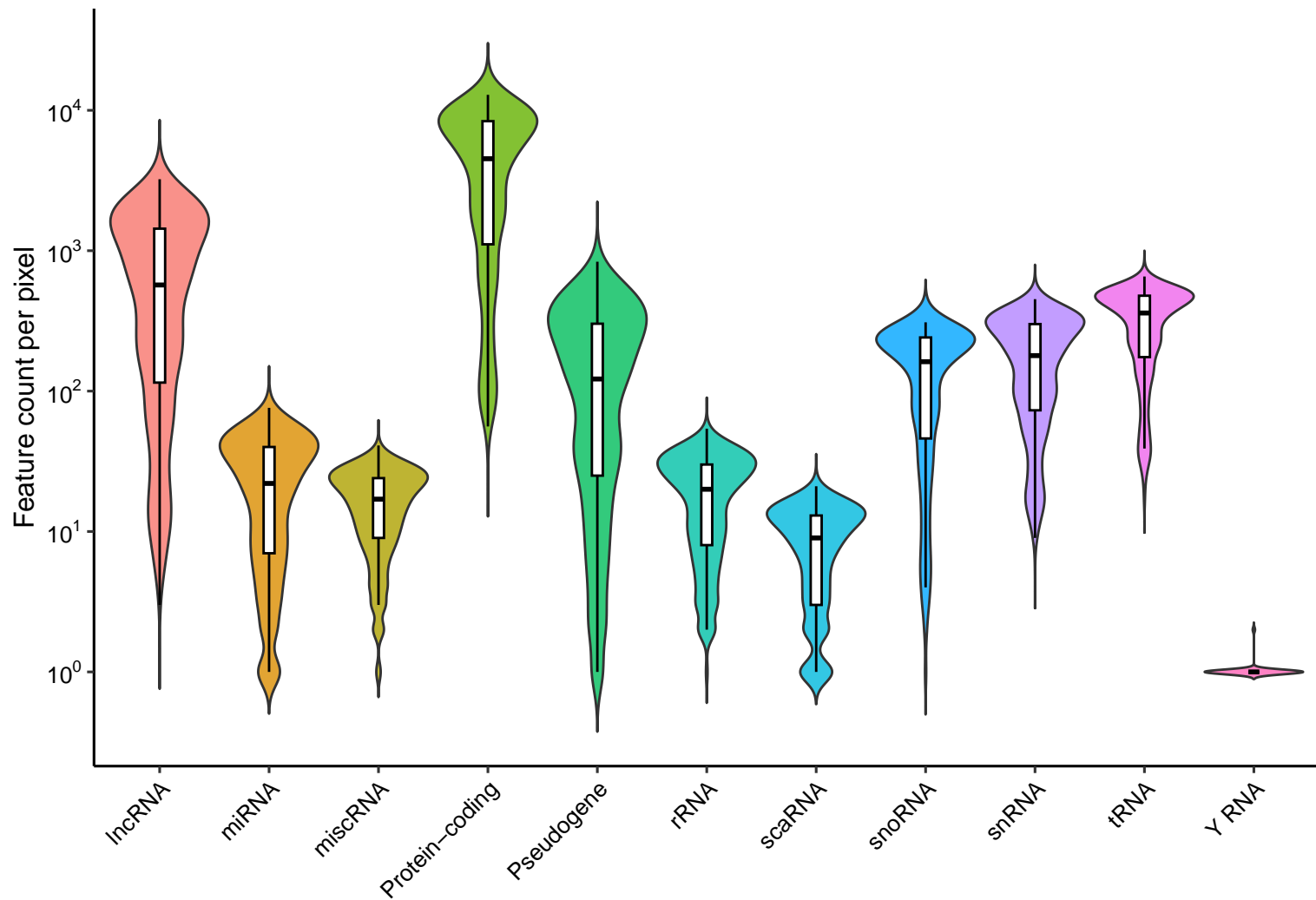

Supplement: btaf688_Supplementary_Data [file btaf688_supplementary_data.zip › Supplementary File 1/Supplementary File 1/the_number_of_gene_feature/ASTRO/Mouse Embryo 1.pdf]

# Mouse Embryo 2

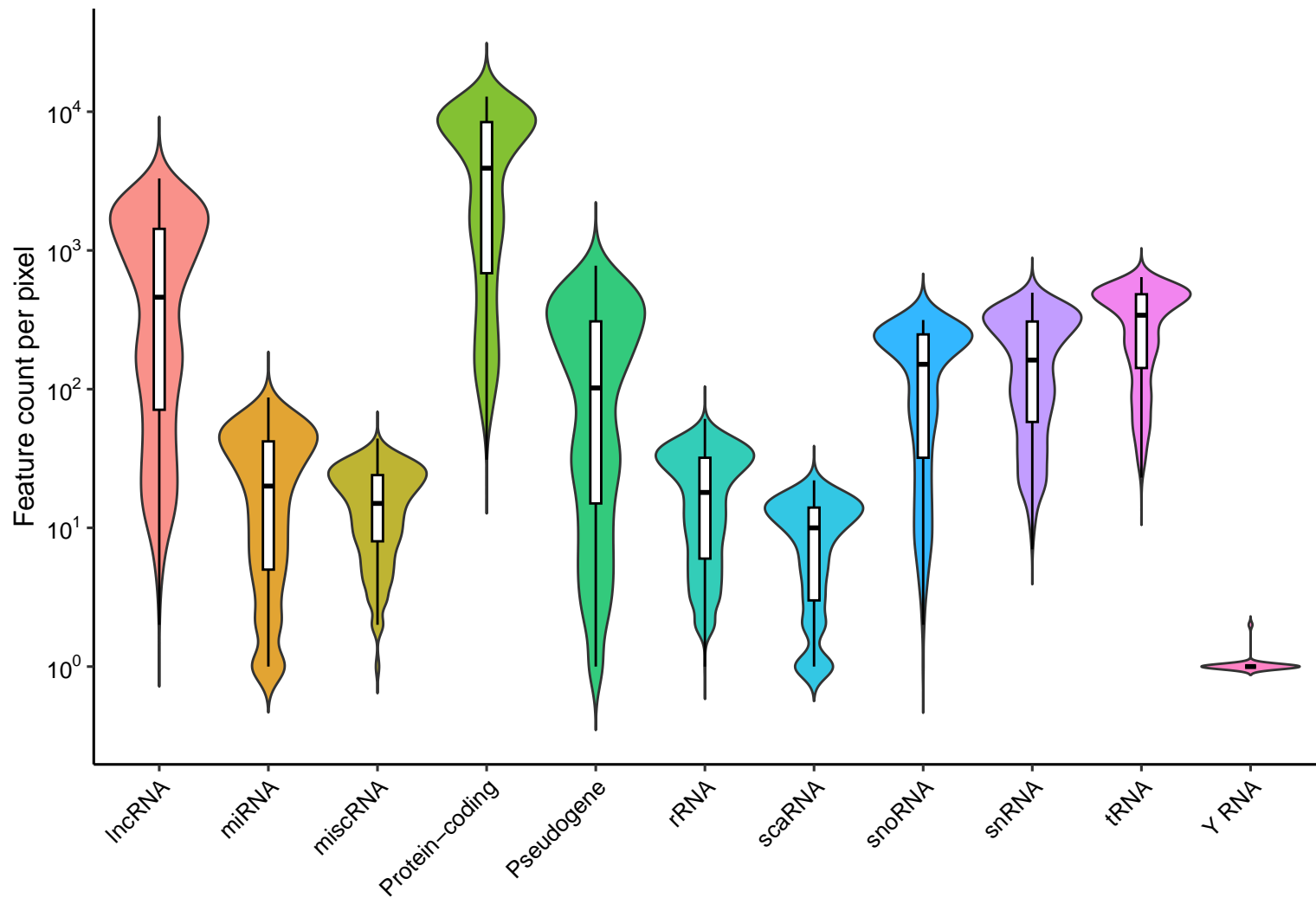

Supplement: btaf688_Supplementary_Data [file btaf688_supplementary_data.zip › Supplementary File 1/Supplementary File 1/the_number_of_gene_feature/ASTRO/Mouse Embryo 2.pdf]

Healthy donor lymph node

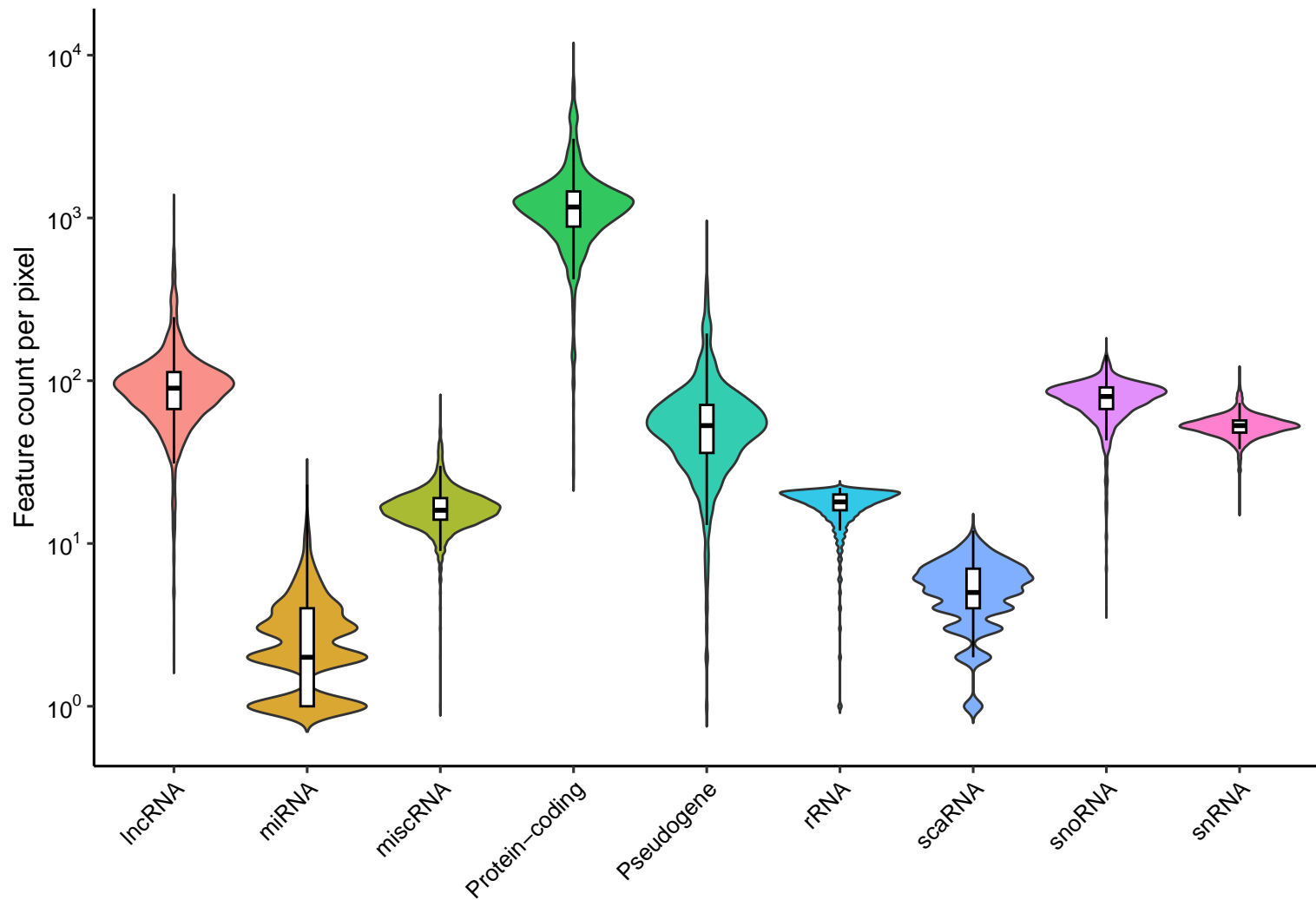

Supplement: btaf688_Supplementary_Data [file btaf688_supplementary_data.zip › Supplementary File 1/Supplementary File 1/the_number_of_gene_feature/ST-pipeline/Healthy donor lymph node.pdf]

# MALT

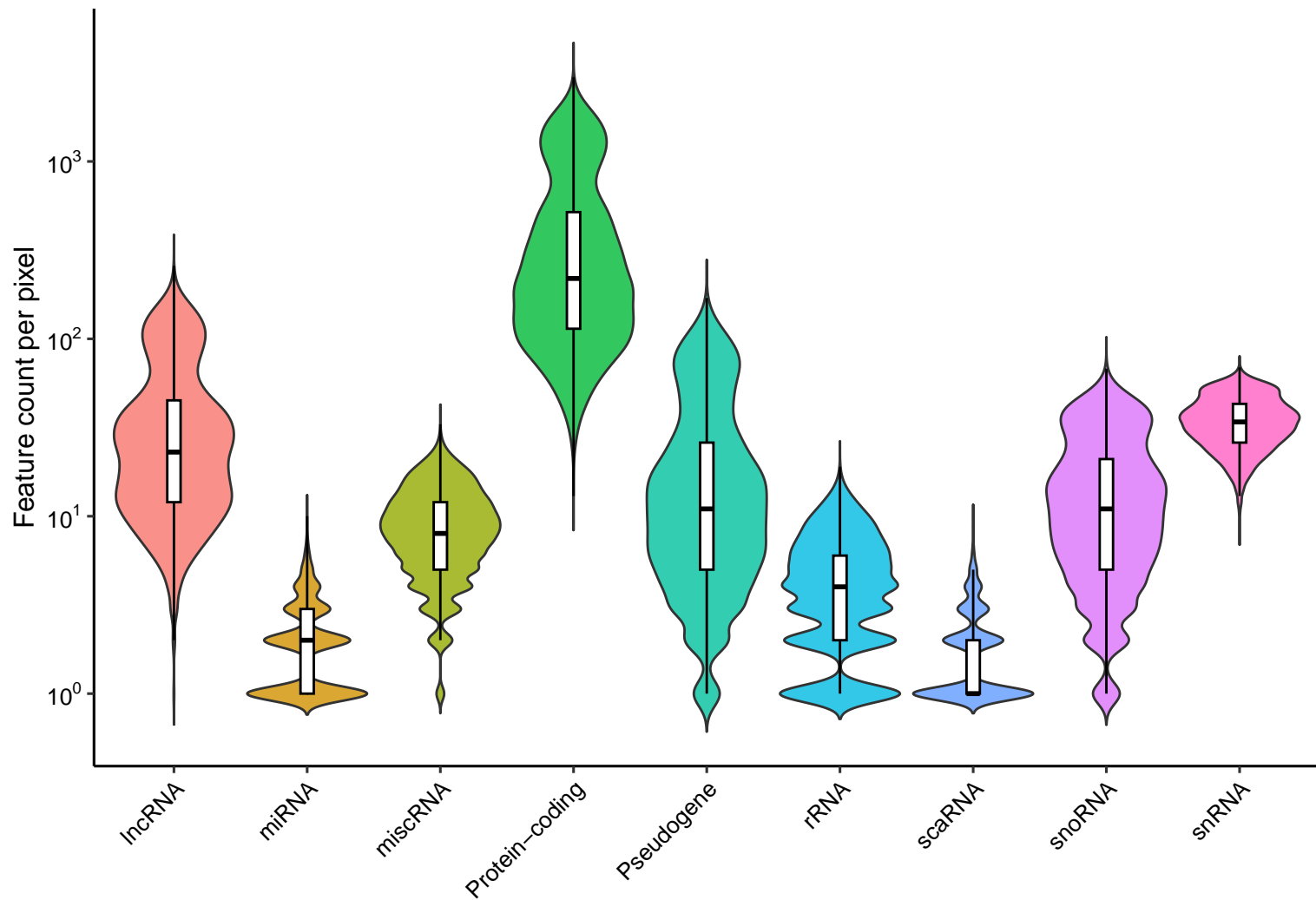

Supplement: btaf688_Supplementary_Data [file btaf688_supplementary_data.zip › Supplementary File 1/Supplementary File 1/the_number_of_gene_feature/ST-pipeline/MALT.pdf]

# Mouse Embryo 1

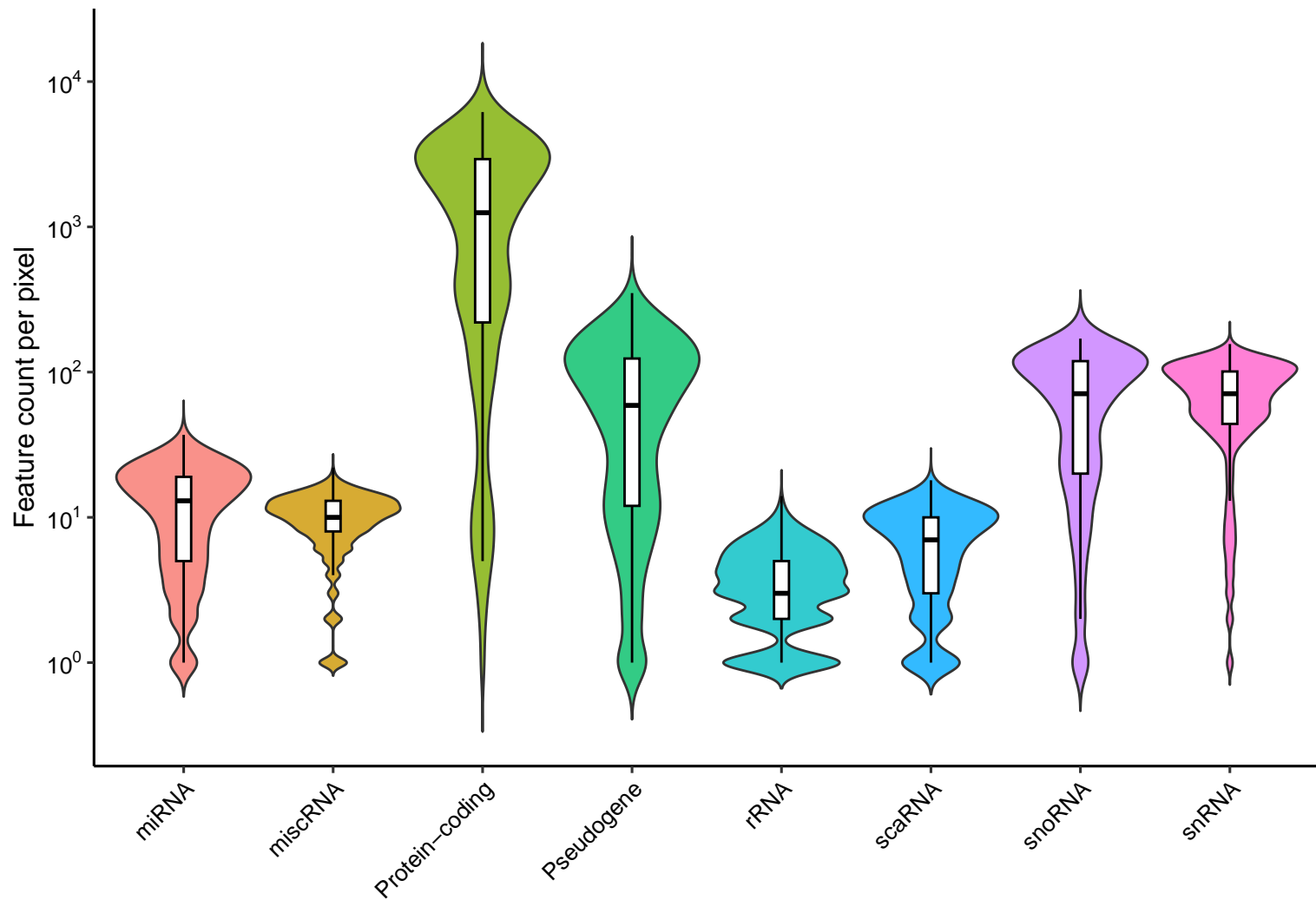

Supplement: btaf688_Supplementary_Data [file btaf688_supplementary_data.zip › Supplementary File 1/Supplementary File 1/the_number_of_gene_feature/ST-pipeline/Mouse Embryo 1.pdf]

# Mouse Embryo 2

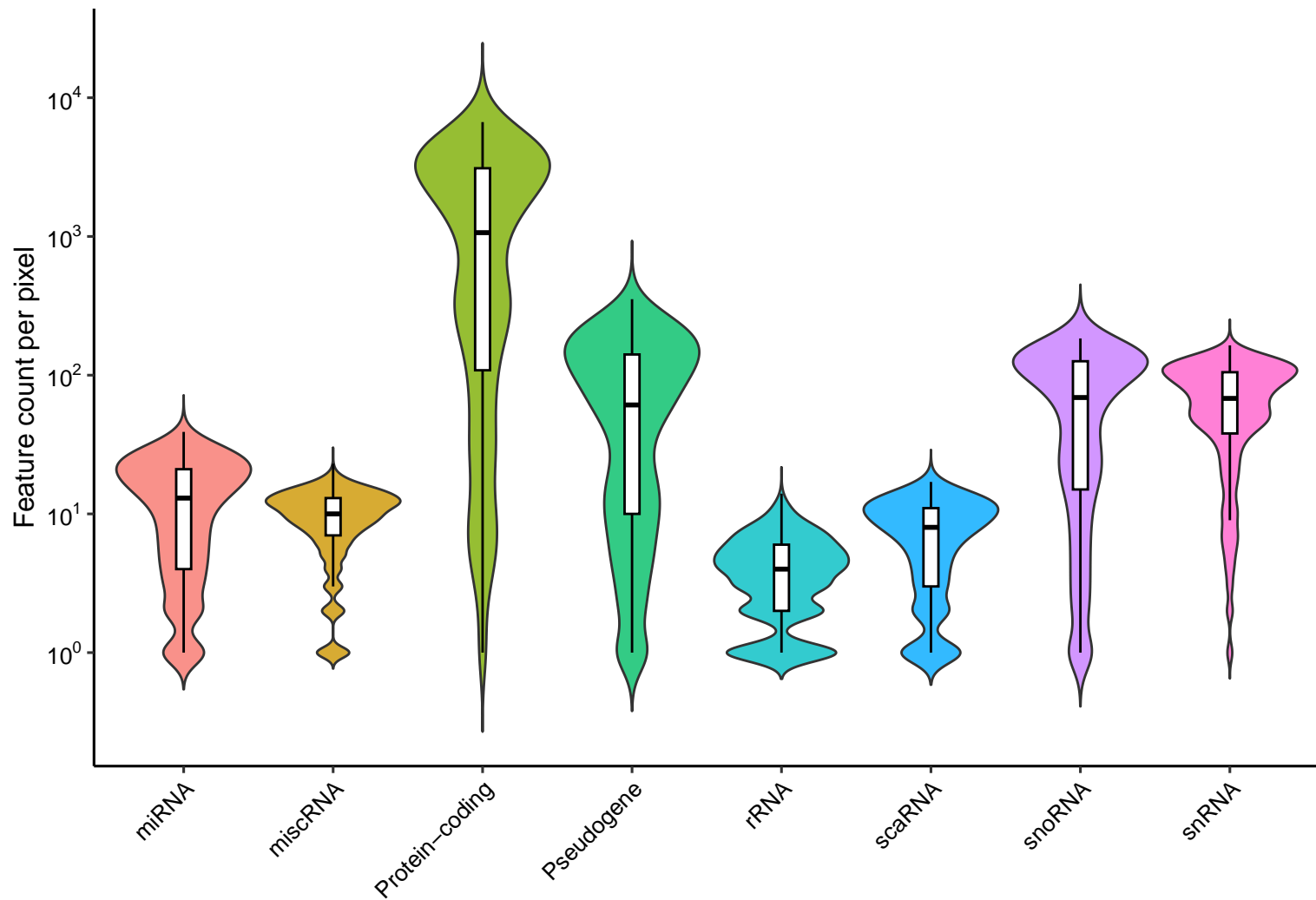

Supplement: btaf688_Supplementary_Data [file btaf688_supplementary_data.zip › Supplementary File 1/Supplementary File 1/the_number_of_gene_feature/ST-pipeline/Mouse Embryo 2.pdf]

# Healthy donor lymph node

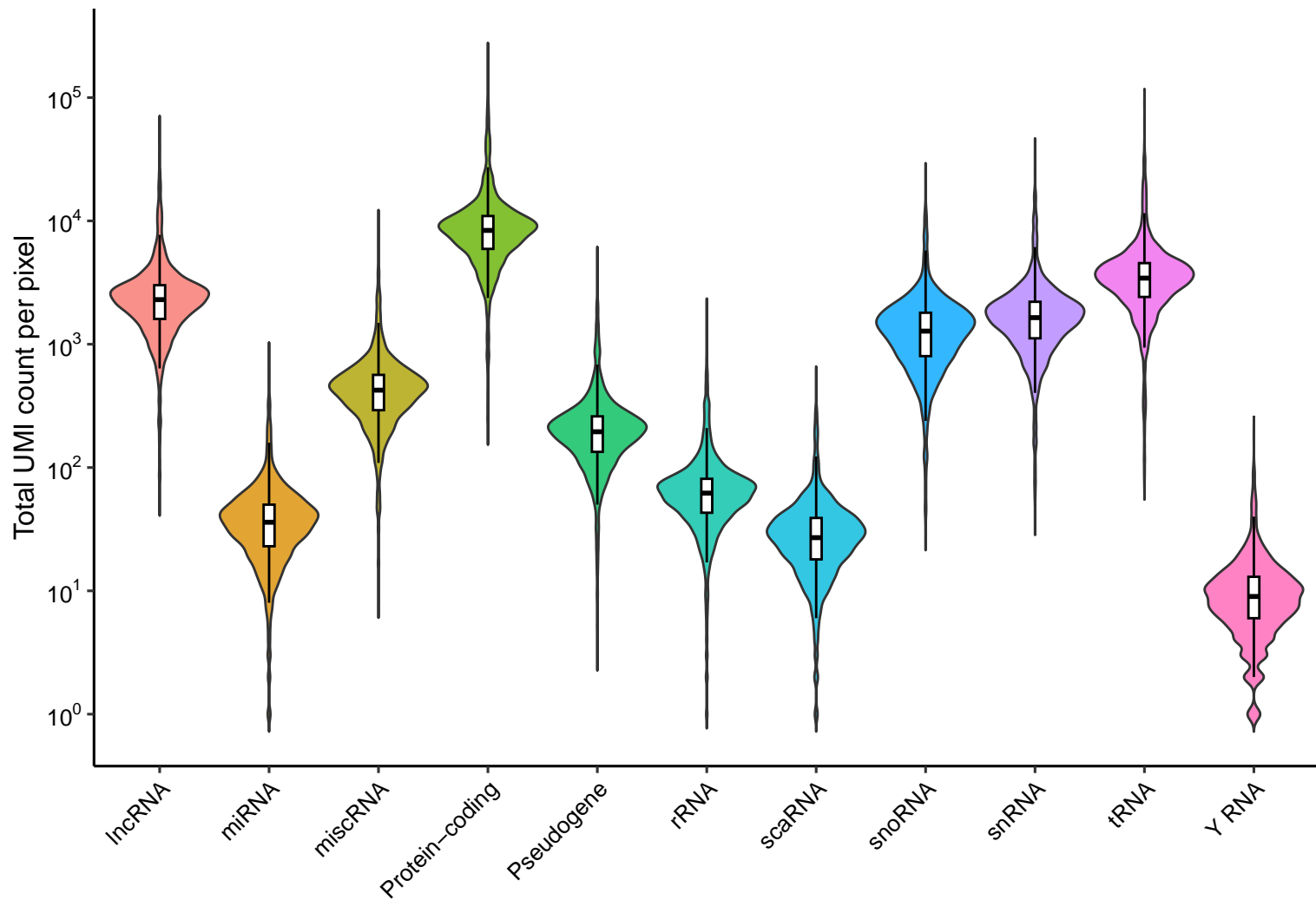

Supplement: btaf688_Supplementary_Data [file btaf688_supplementary_data.zip › Supplementary File 1/Supplementary File 1/the_number_of_UMI/ASTRO/Healthy donor lymph node.pdf]

# MALT

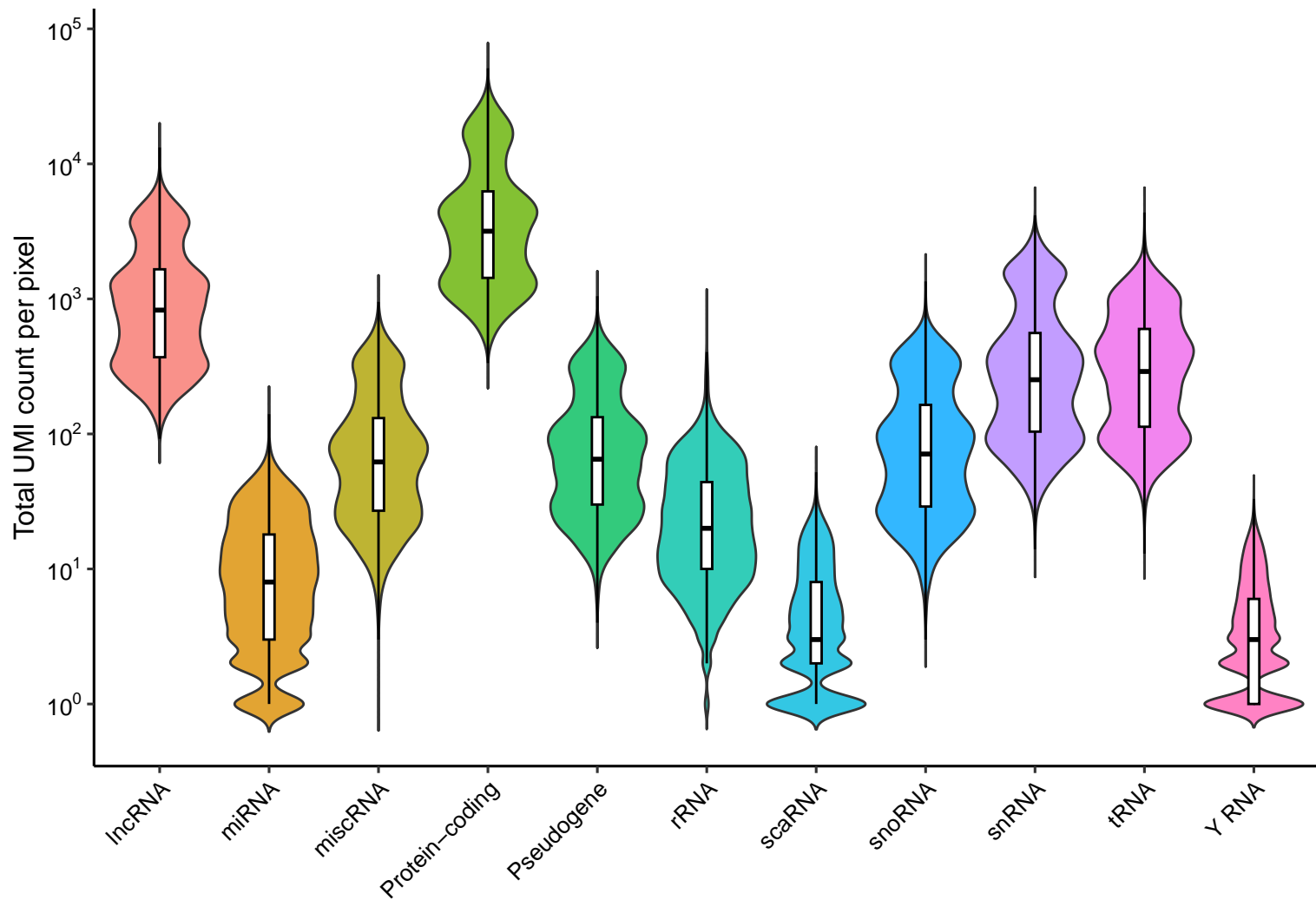

Supplement: btaf688_Supplementary_Data [file btaf688_supplementary_data.zip › Supplementary File 1/Supplementary File 1/the_number_of_UMI/ASTRO/MALT.pdf]

# Mouse Embryo 1

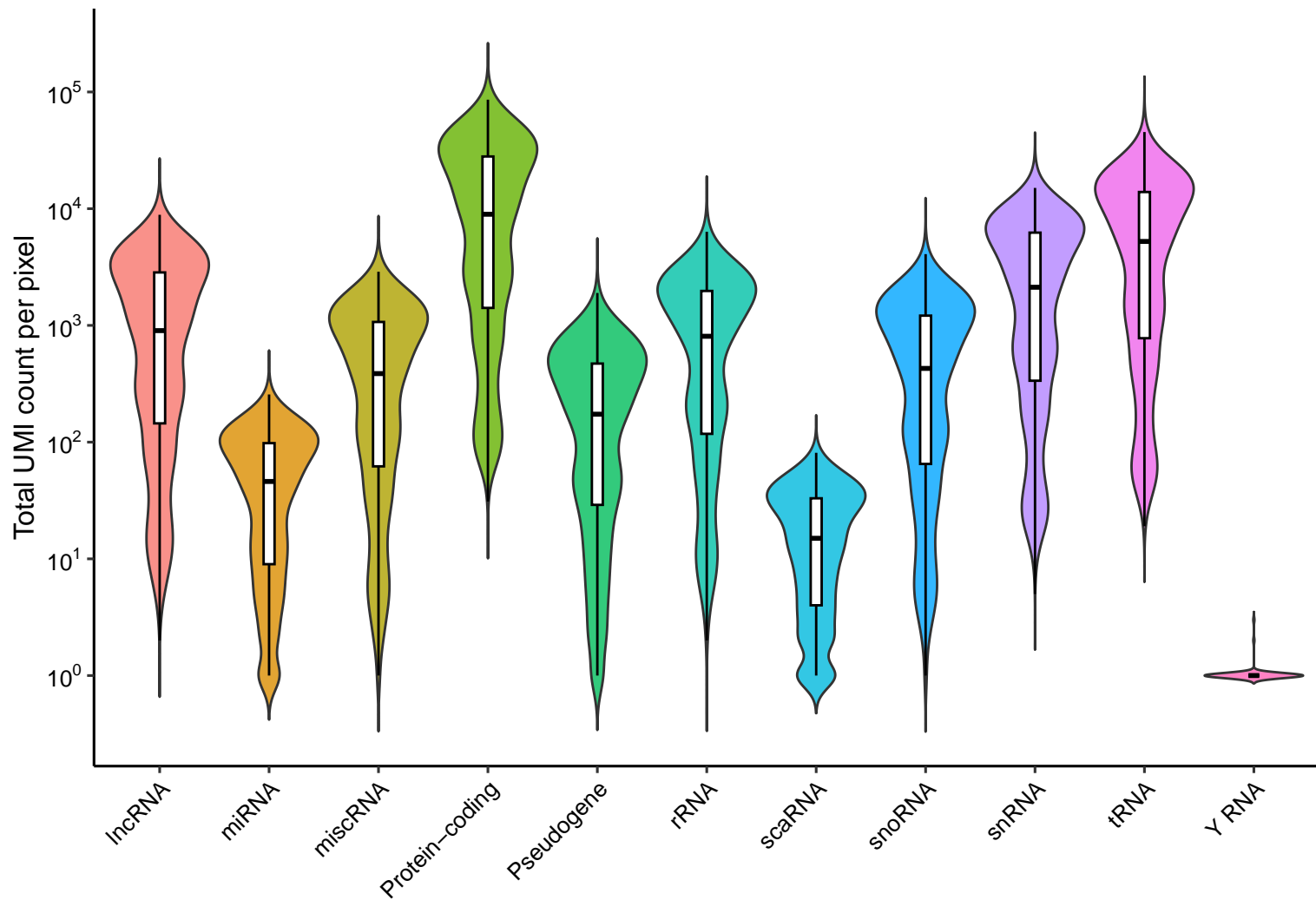

Supplement: btaf688_Supplementary_Data [file btaf688_supplementary_data.zip › Supplementary File 1/Supplementary File 1/the_number_of_UMI/ASTRO/Mouse Embryo 1.pdf]

## Mouse Embryo 2

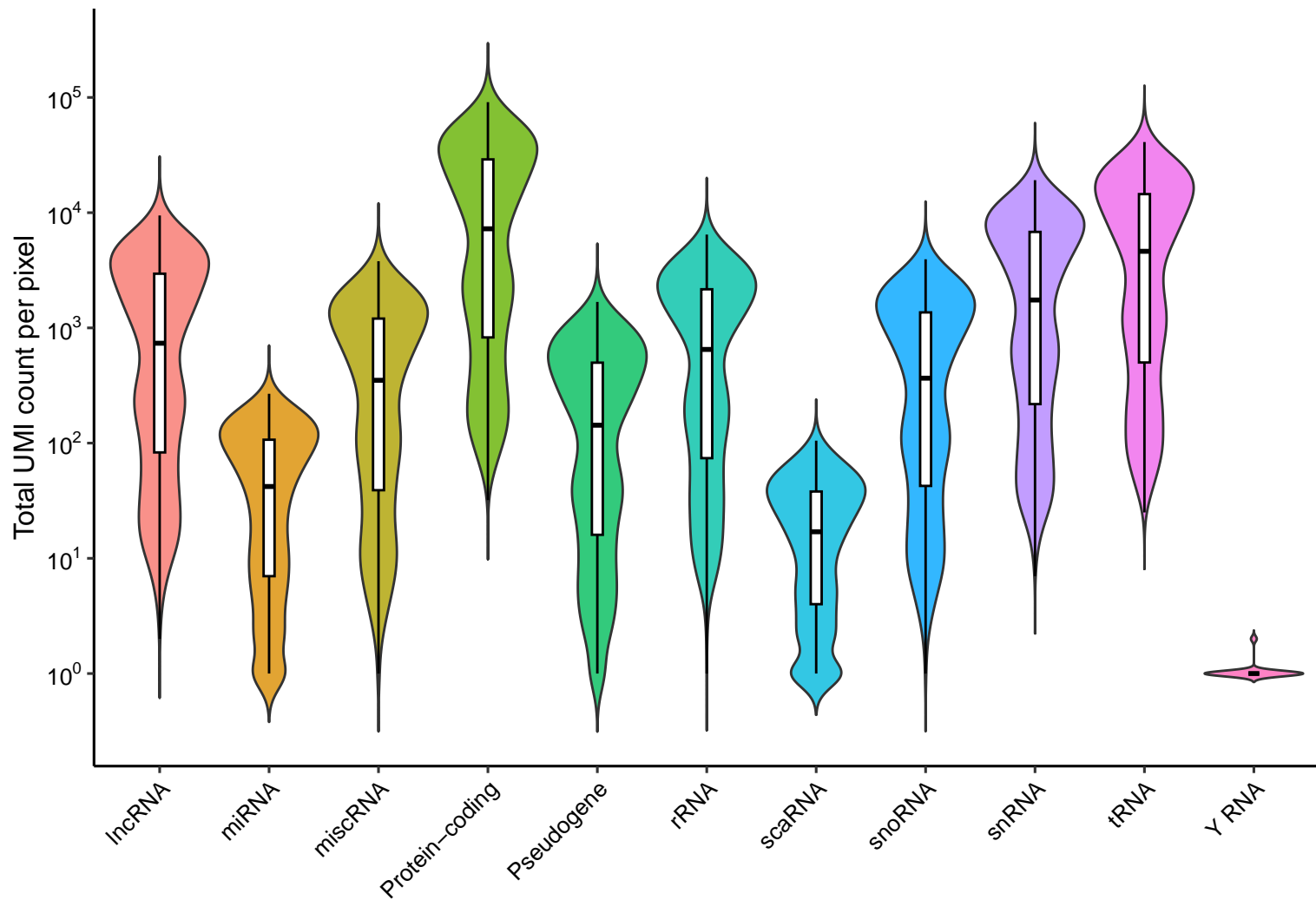

Supplement: btaf688_Supplementary_Data [file btaf688_supplementary_data.zip › Supplementary File 1/Supplementary File 1/the_number_of_UMI/ASTRO/Mouse Embryo 2.pdf]

# Healthy donor lymph node

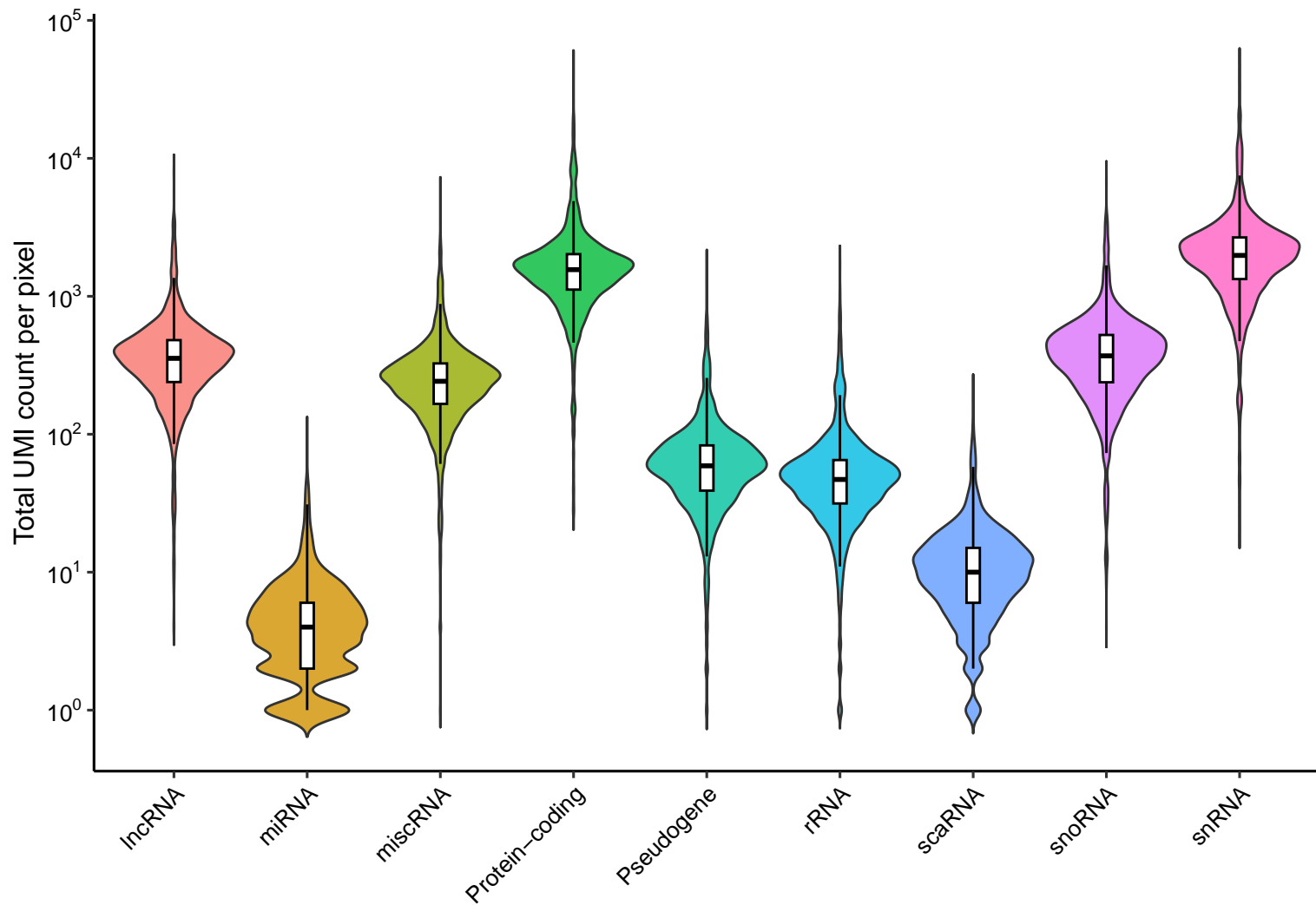

Supplement: btaf688_Supplementary_Data [file btaf688_supplementary_data.zip › Supplementary File 1/Supplementary File 1/the_number_of_UMI/ST-pipeline/Healthy donor lymph node.pdf]

# MALT

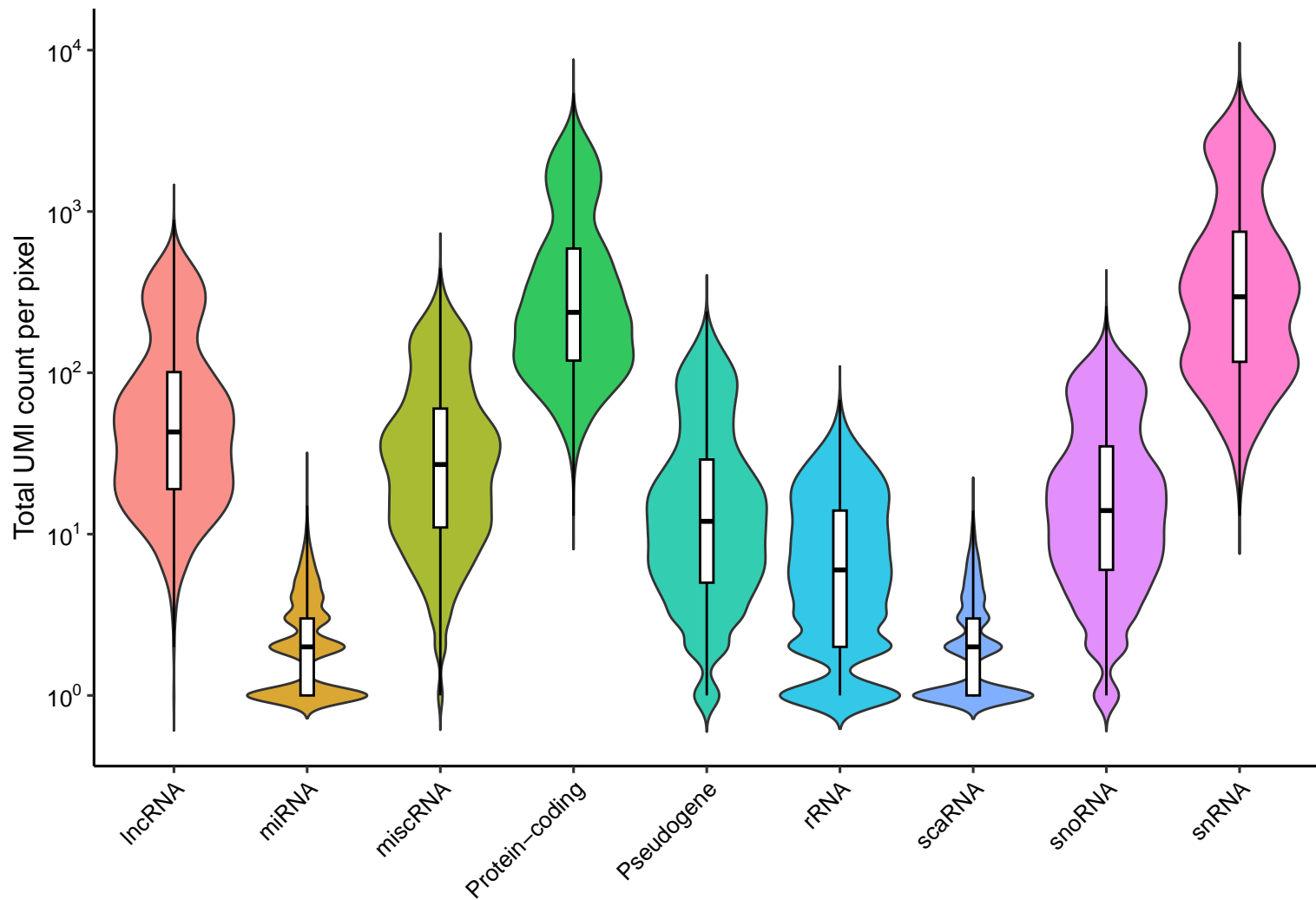

Supplement: btaf688_Supplementary_Data [file btaf688_supplementary_data.zip › Supplementary File 1/Supplementary File 1/the_number_of_UMI/ST-pipeline/MALT.pdf]

# Mouse Embryo 1

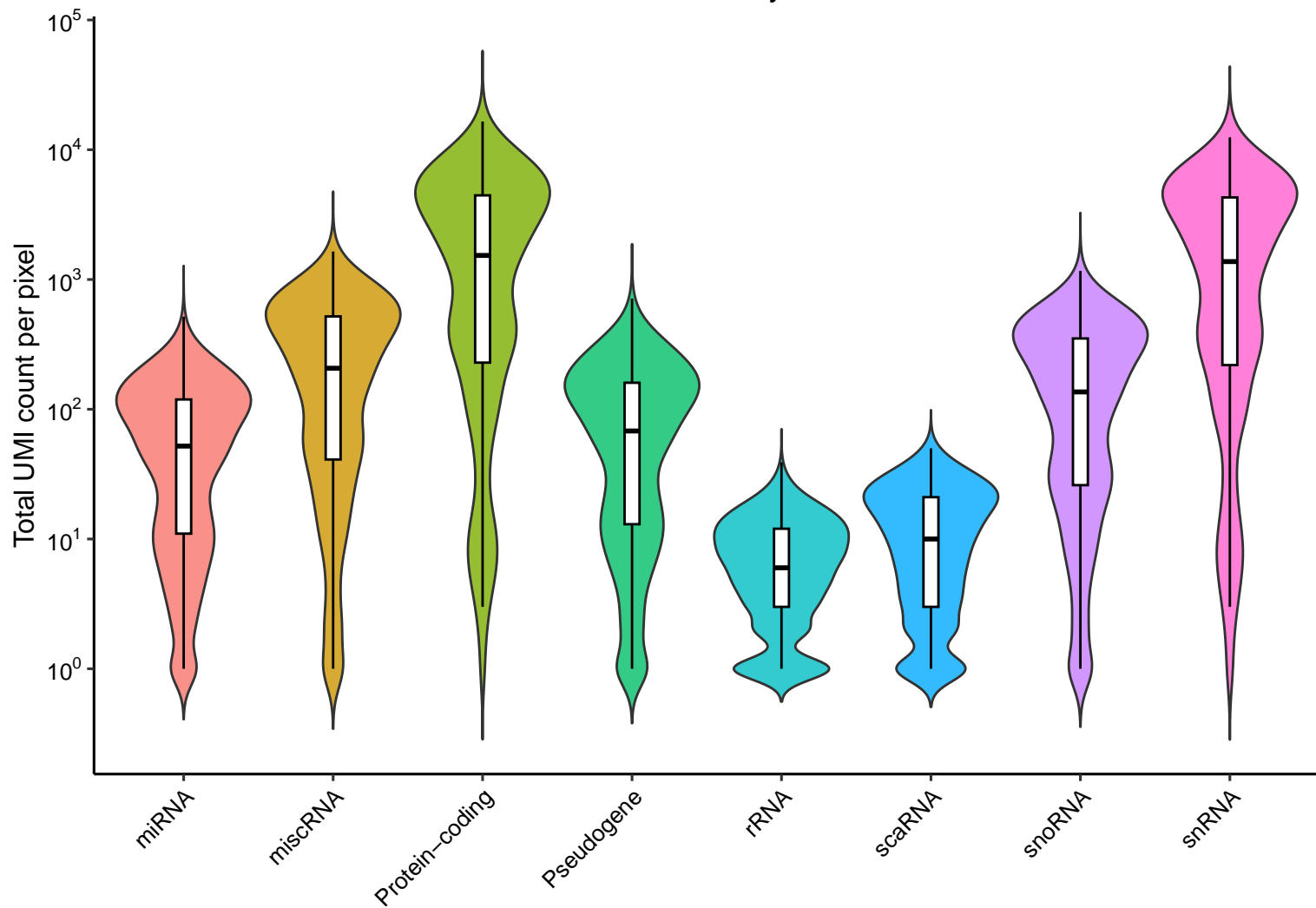

Supplement: btaf688_Supplementary_Data [file btaf688_supplementary_data.zip › Supplementary File 1/Supplementary File 1/the_number_of_UMI/ST-pipeline/Mouse Embryo 1.pdf]

# Mouse Embryo 2

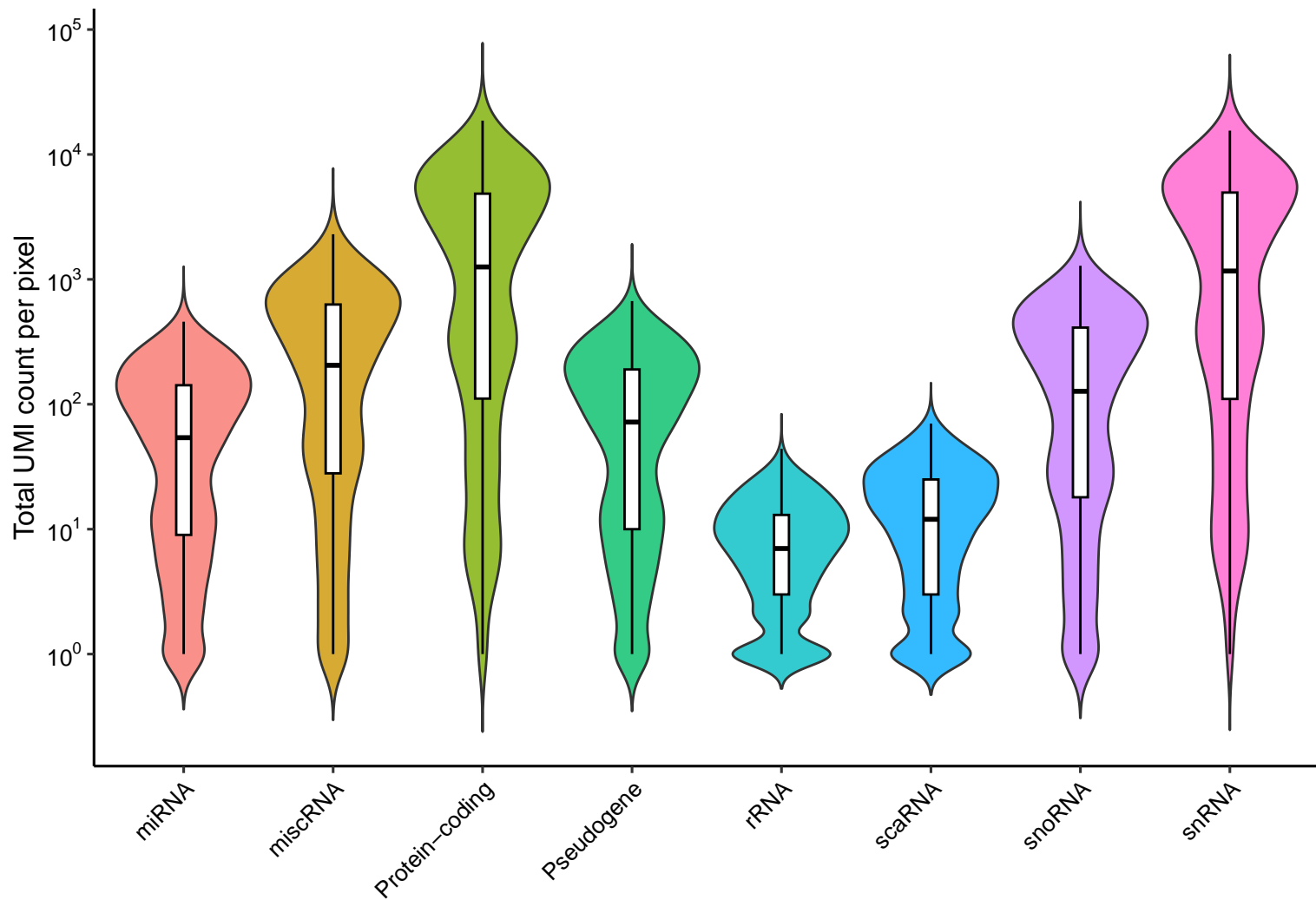

Supplement: btaf688_Supplementary_Data [file btaf688_supplementary_data.zip › Supplementary File 1/Supplementary File 1/the_number_of_UMI/ST-pipeline/Mouse Embryo 2.pdf]
