# Supplementary material for: ASTRO: Automated Spatial-Transcriptome whole RNA Output
Source: Bioinformatics. 2026 Jan 6;42(2):btaf688. doi: 10.1093/bioinformatics/btaf688 (PMC12866646; doi:10.1093/bioinformatics/btaf688)
Supplement: btaf688_Supplementary_Data [file btaf688_supplementary_data.zip › Supplementary File 1.zip!/Supplementary File 1/Spatial_mapping/the_number_of_gene_feature/ST-pipeline/Mouse Embryo 1.pdf]

miRNA

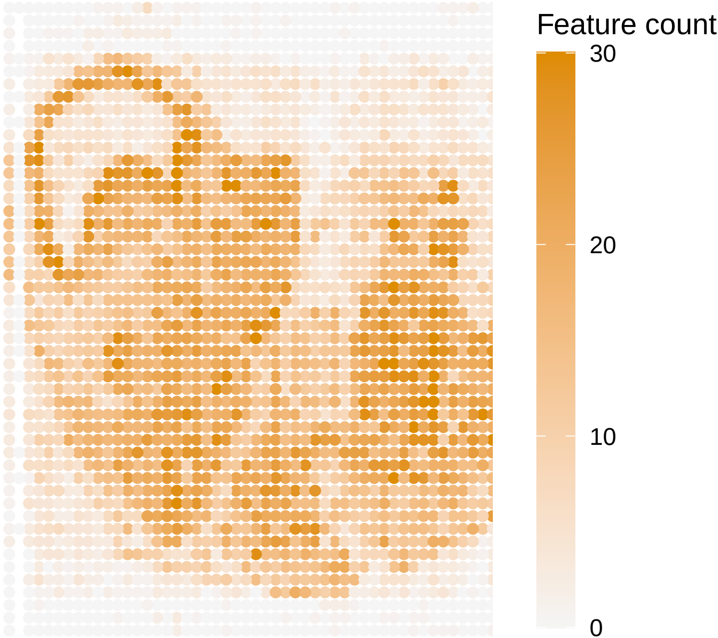

misc_RNA

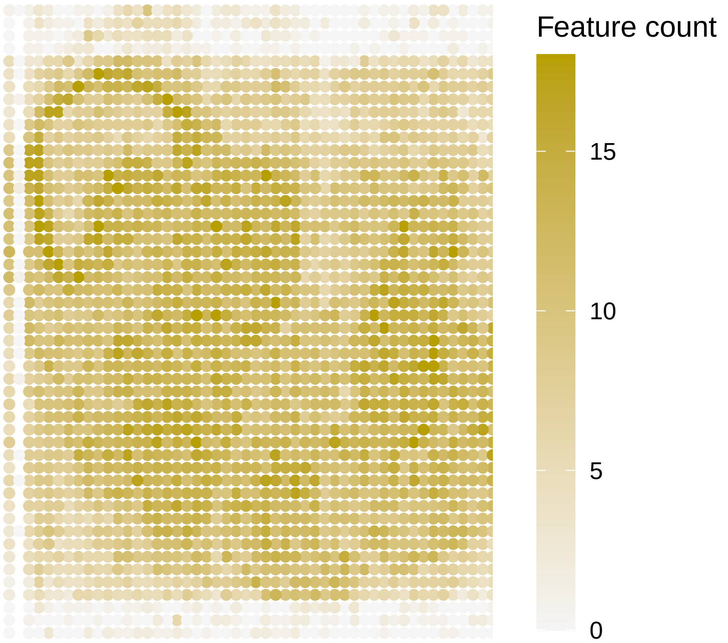

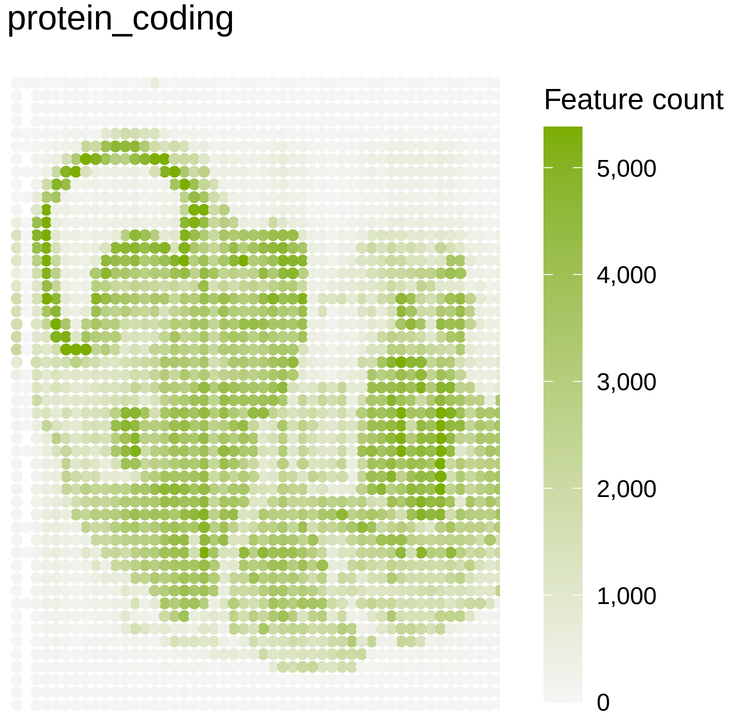
protein_coding
Feature count
5,000
4,000
3,000
2,000
1,000
0

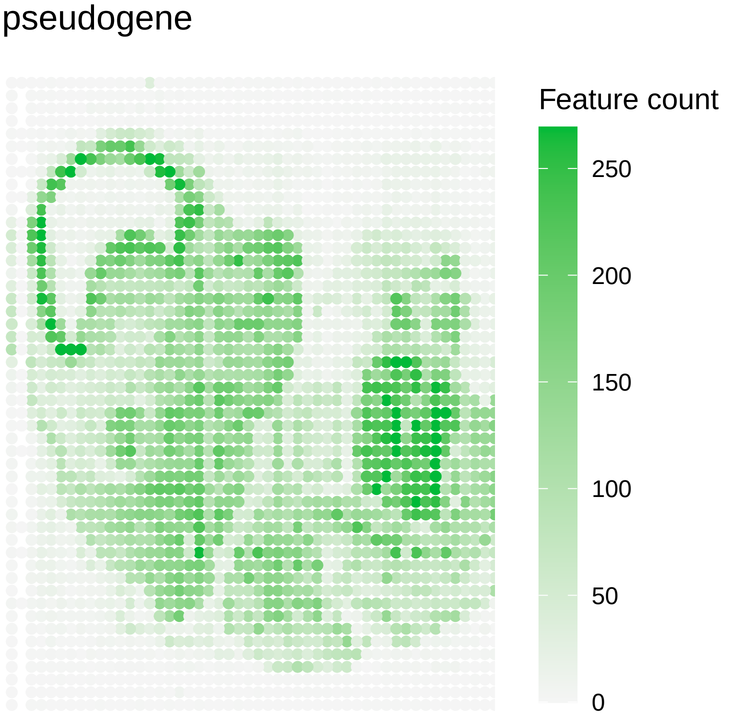
pseudogene
Feature count
250
200
150
100
50
0

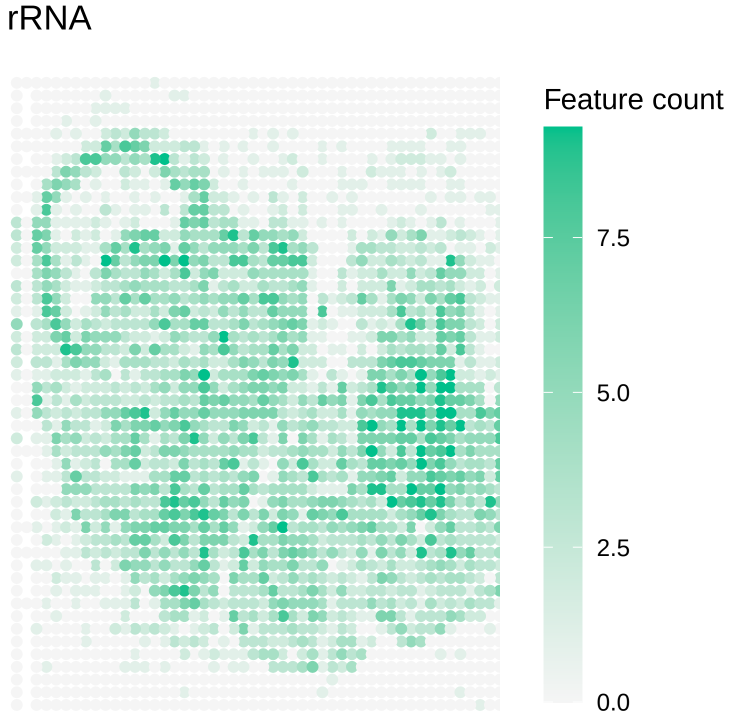
rRNA
Feature count
7.5
5.0
2.5
0.0

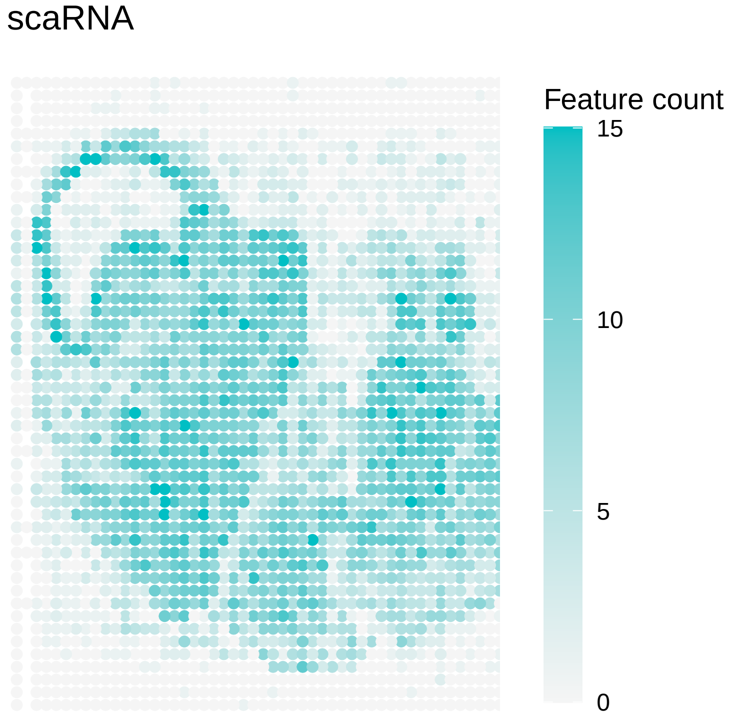
scaRNA
Feature count
15
10
5
0

snRNA

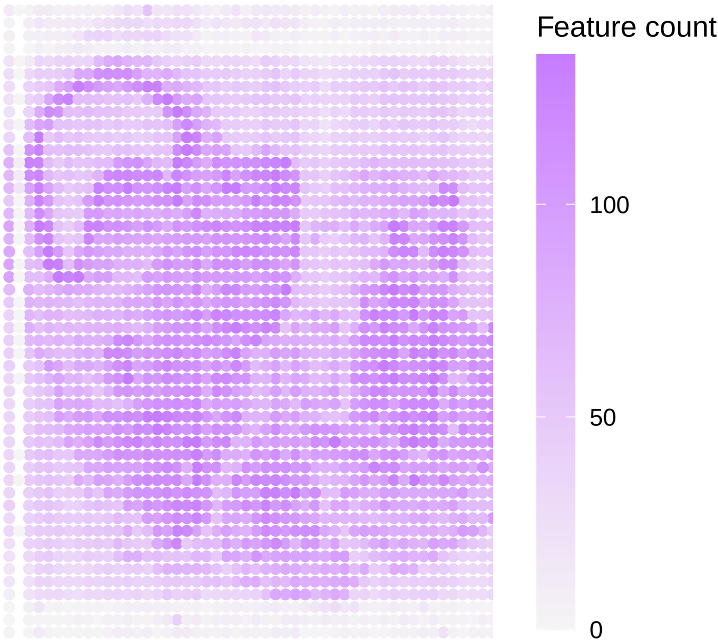

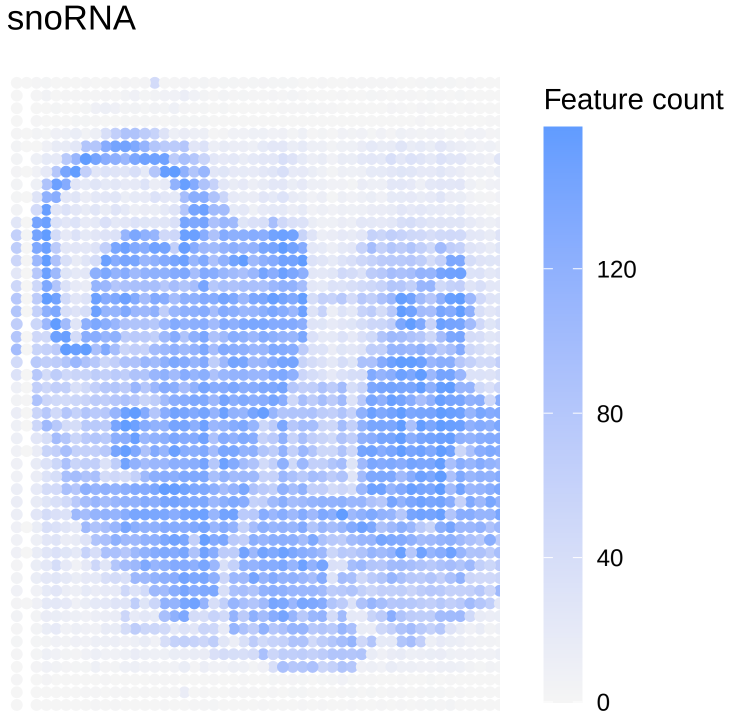
snoRNA
Feature count
120
80
40
0
