# Supplementary material for: ASTRO: Automated Spatial-Transcriptome whole RNA Output
Source: Bioinformatics. 2026 Jan 6;42(2):btaf688. doi: 10.1093/bioinformatics/btaf688 (PMC12866646; doi:10.1093/bioinformatics/btaf688)

miRNA

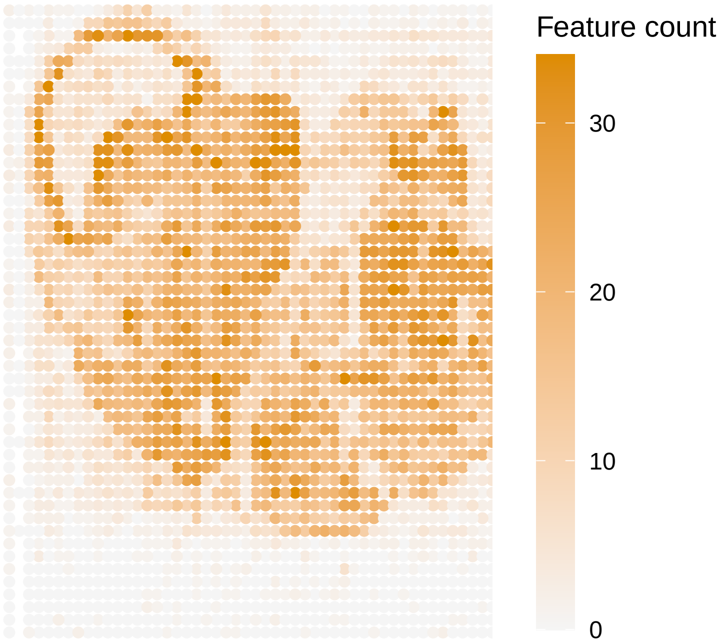

misc_RNA

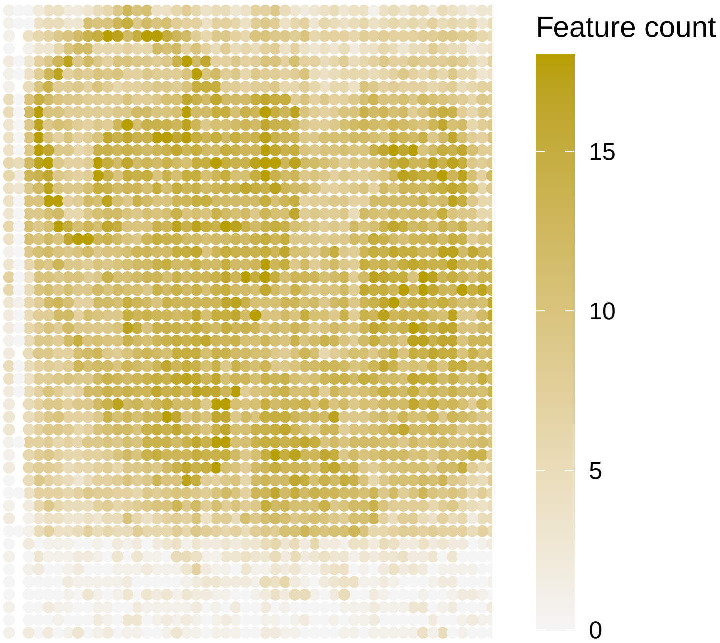

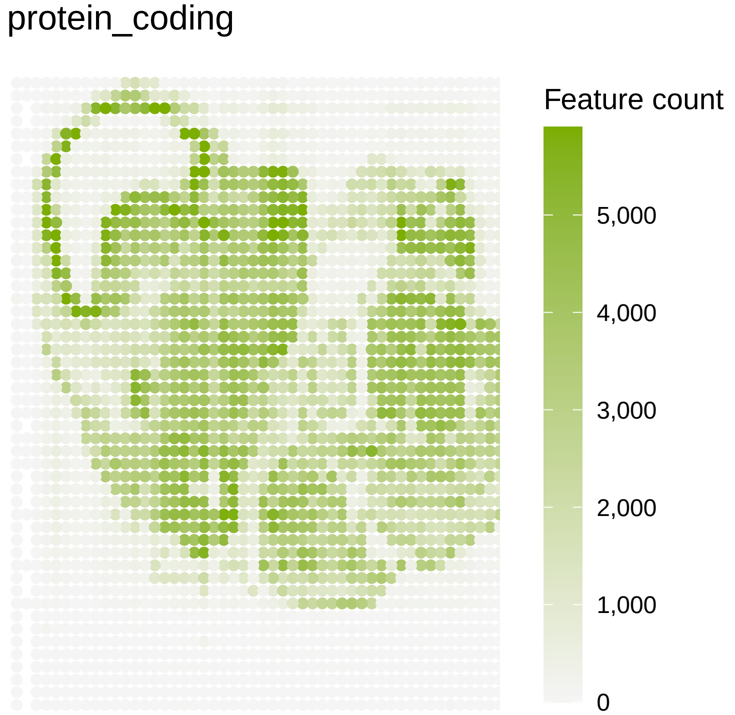

protein_coding
Feature count
5,000
4,000
3,000
2,000
1,000
0

pseudogene

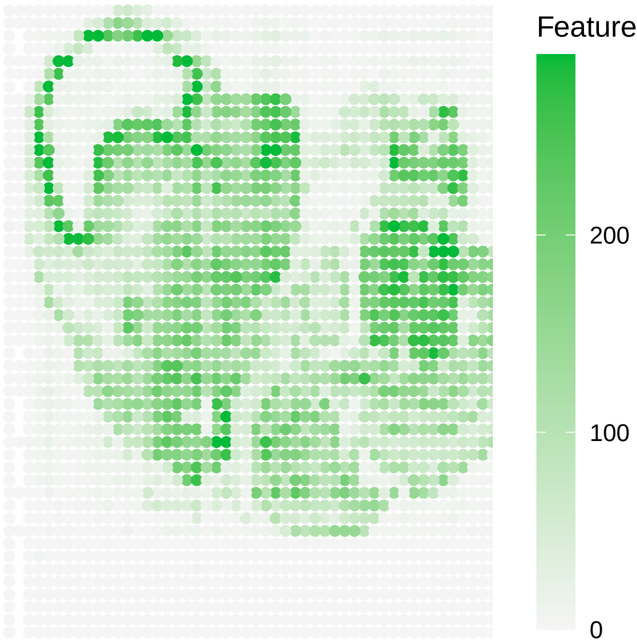

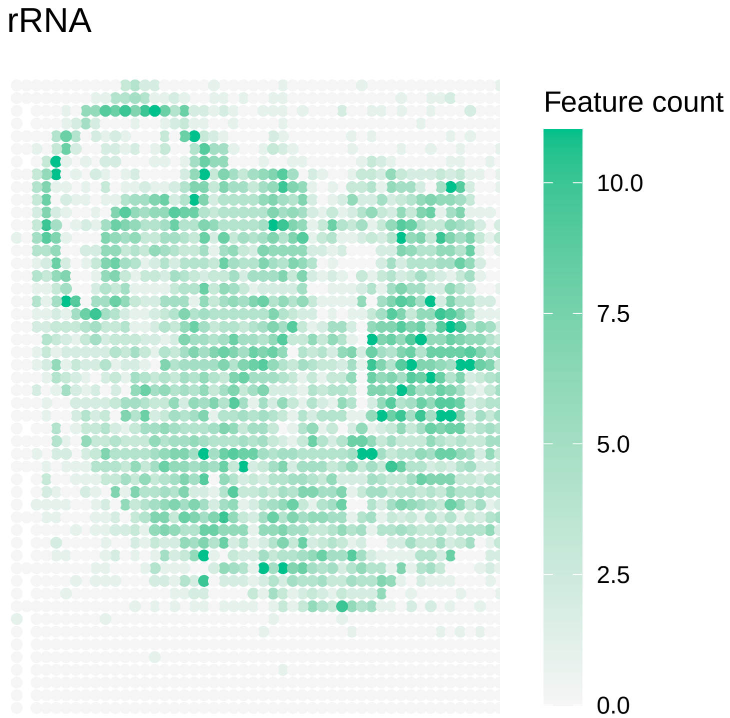

rRNA
Feature count
10.0
7.5
5.0
2.5
0.0

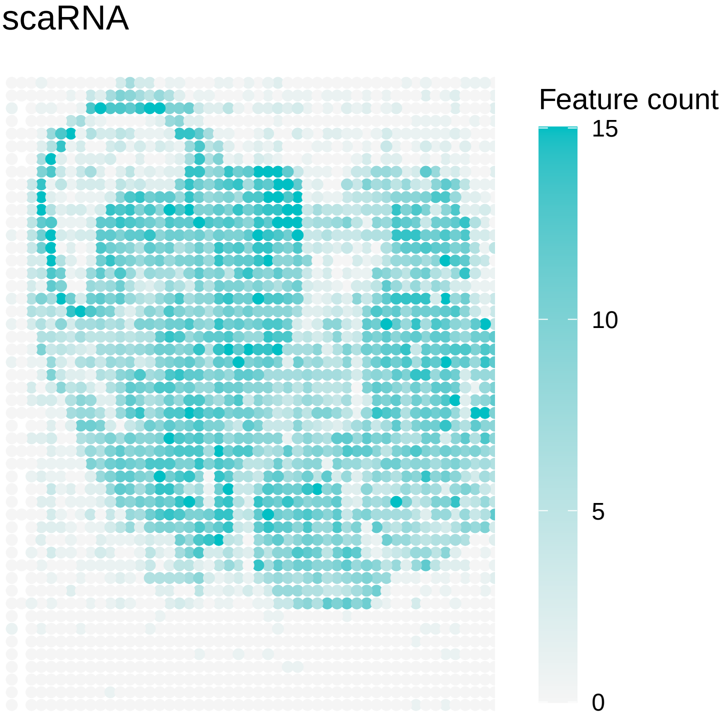

scaRNA
Feature count
15
10
5
0

snRNA

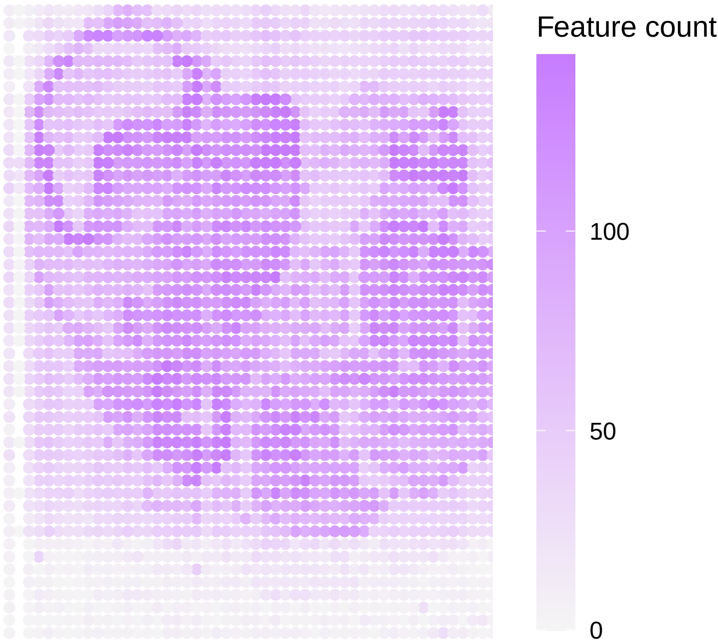

snoRNA

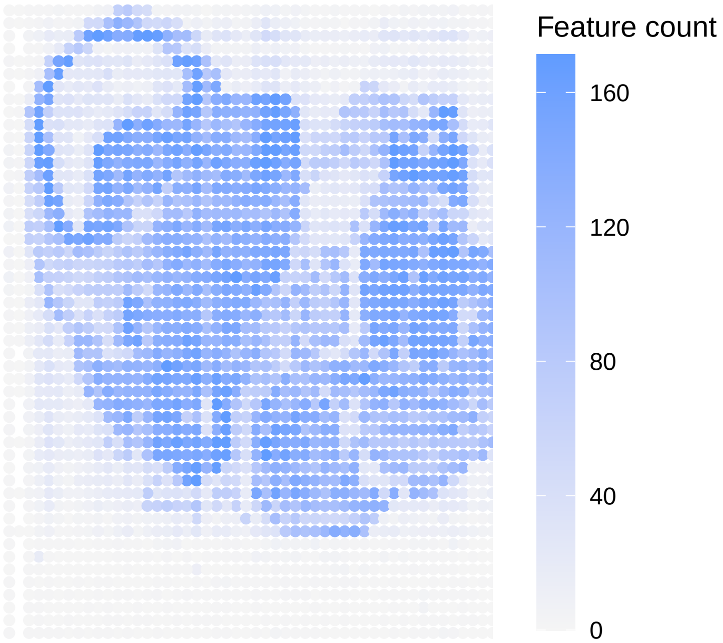

Supplement: btaf688_Supplementary_Data [file btaf688_supplementary_data.zip › Supplementary File 1.zip!/Supplementary File 1/Spatial_mapping/the_number_of_gene_feature/ST-pipeline/Mouse Embryo 2.pdf]
