# Supplementary material for: ASTRO: Automated Spatial-Transcriptome whole RNA Output
Source: Bioinformatics. 2026 Jan 6;42(2):btaf688. doi: 10.1093/bioinformatics/btaf688 (PMC12866646; doi:10.1093/bioinformatics/btaf688)
Supplement: btaf688_Supplementary_Data [file btaf688_supplementary_data.zip › Supplementary File 1.zip!/Supplementary File 1/Spatial_mapping/the_number_of_gene_feature/ST-pipeline/Healthy donor lymph node.pdf]

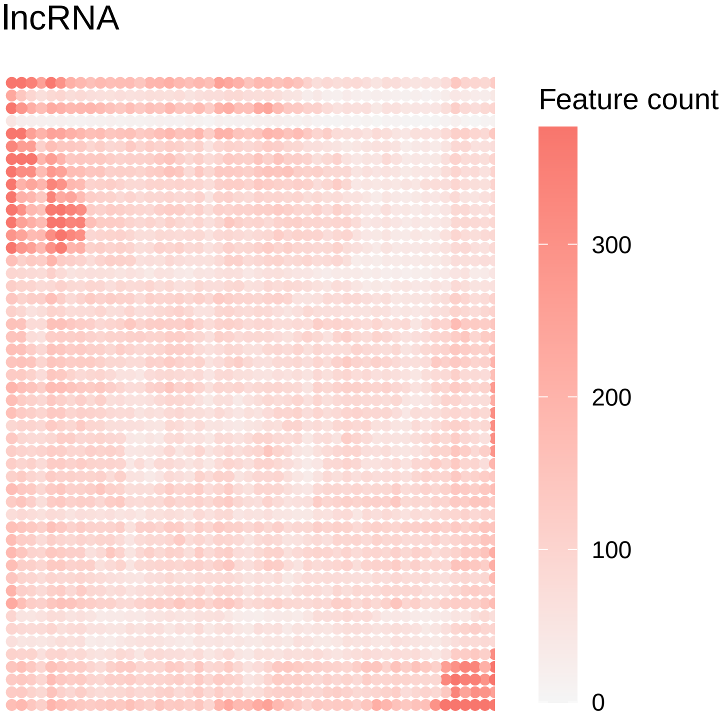
lncRNA
Feature count
300
200
100
0

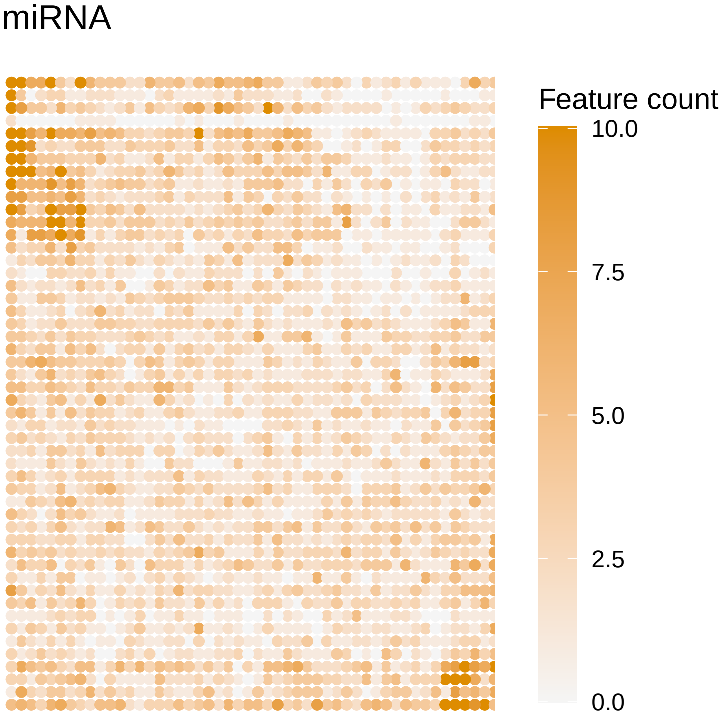
miRNA
Feature count
10.0
7.5
5.0
2.5
0.0

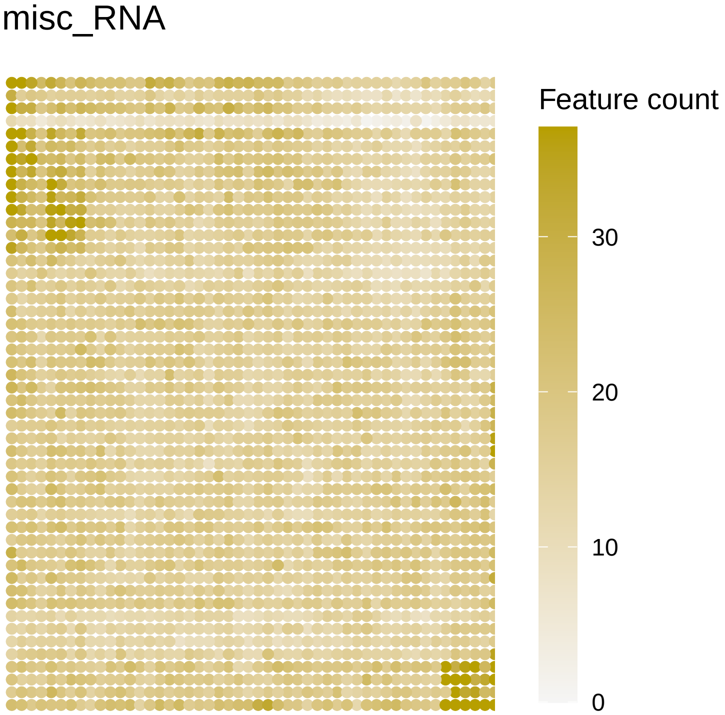
misc_RNA
Feature count
30
20
10
0

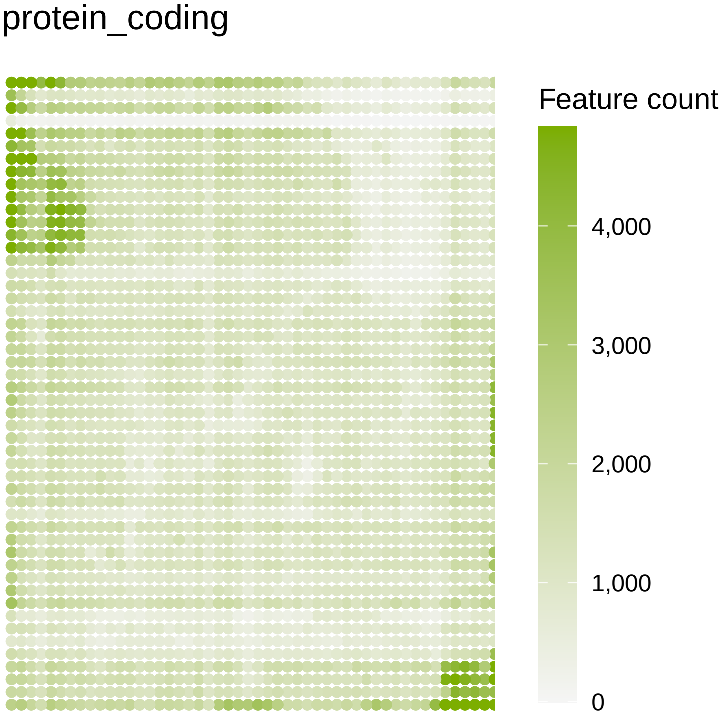
protein_coding
Feature count
4,000
3,000
2,000
1,000
0

pseudogene

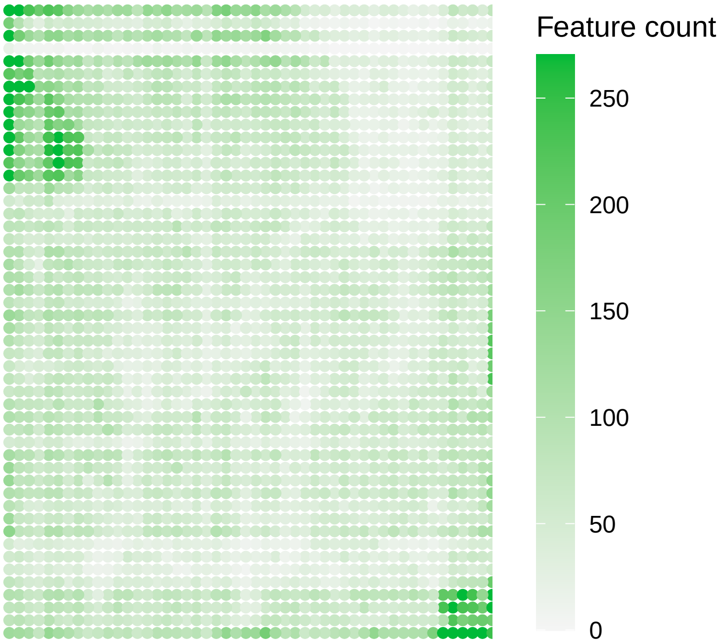

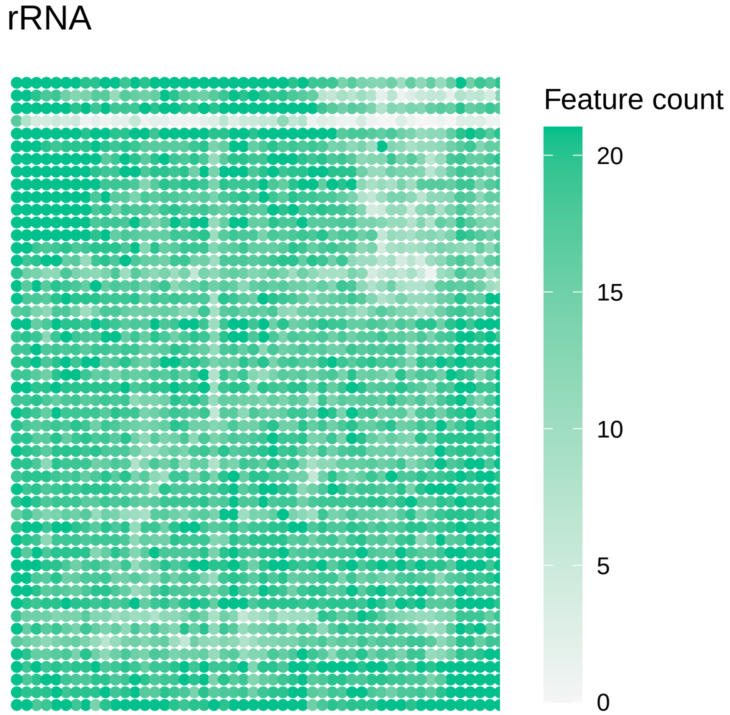
rRNA
Feature count
20
15
10
5
0

scaRNA

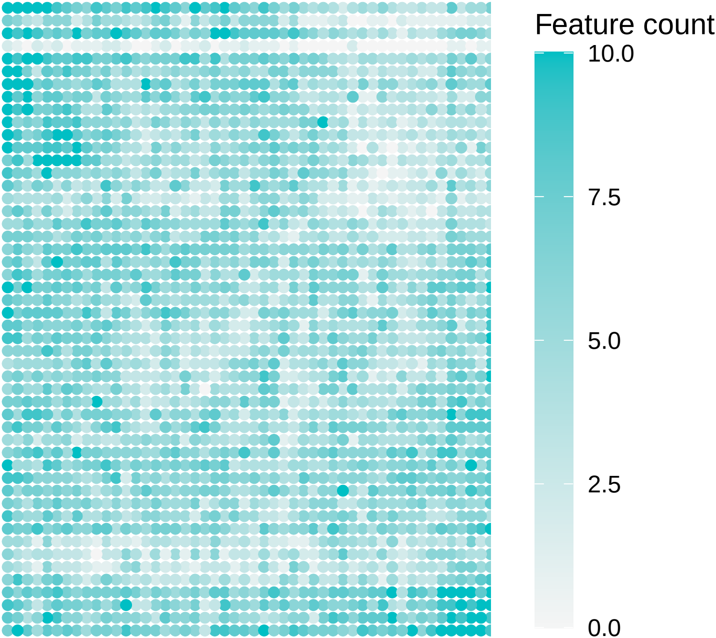

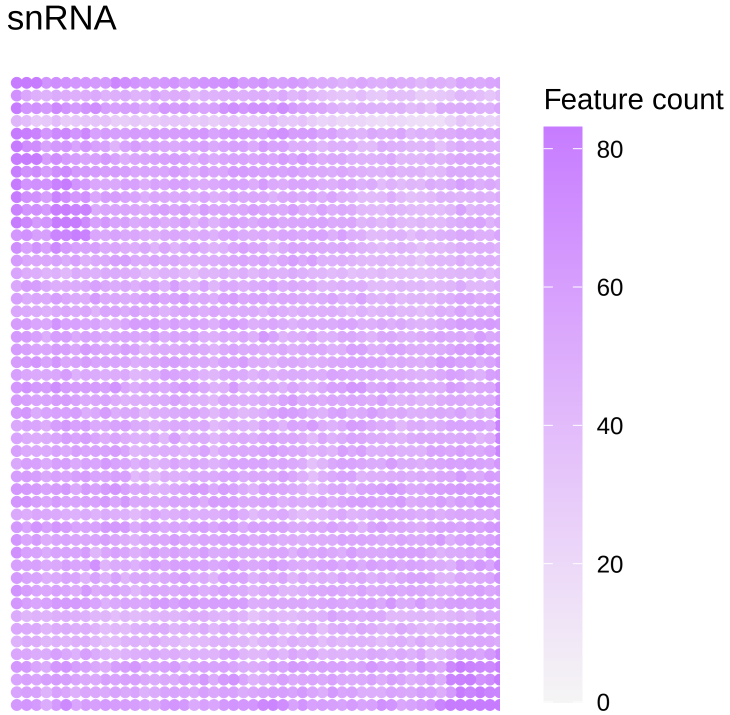

snRNA
Feature count
80
60
40
20
0

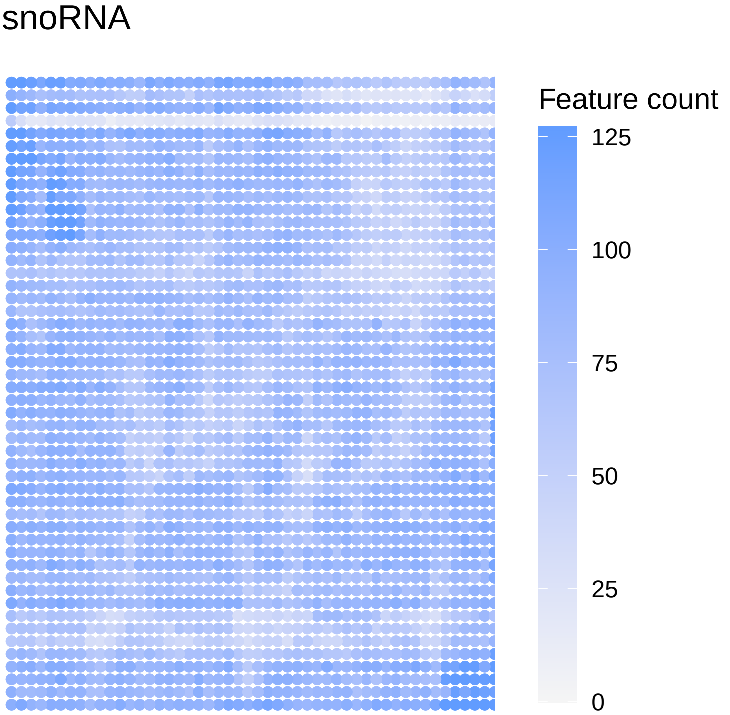
snoRNA
Feature count
125
100
75
50
25
0
