# Supplementary material for: ASTRO: Automated Spatial-Transcriptome whole RNA Output
Source: Bioinformatics. 2026 Jan 6;42(2):btaf688. doi: 10.1093/bioinformatics/btaf688 (PMC12866646; doi:10.1093/bioinformatics/btaf688)

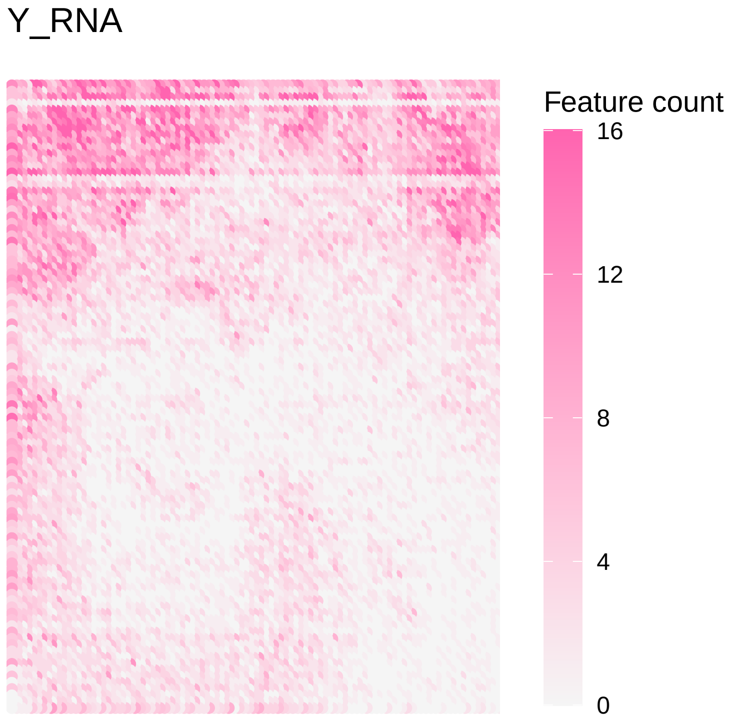
Y_RNA
Feature count
16
12
8
4
0

lncRNA

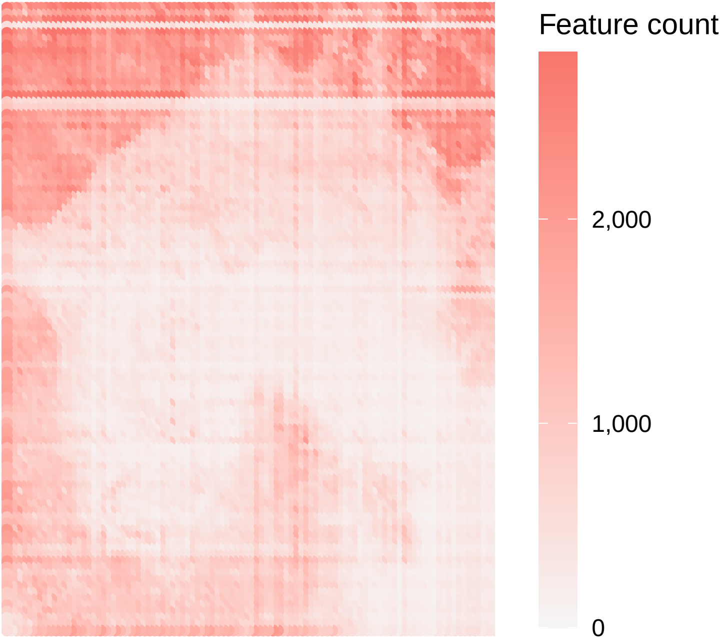

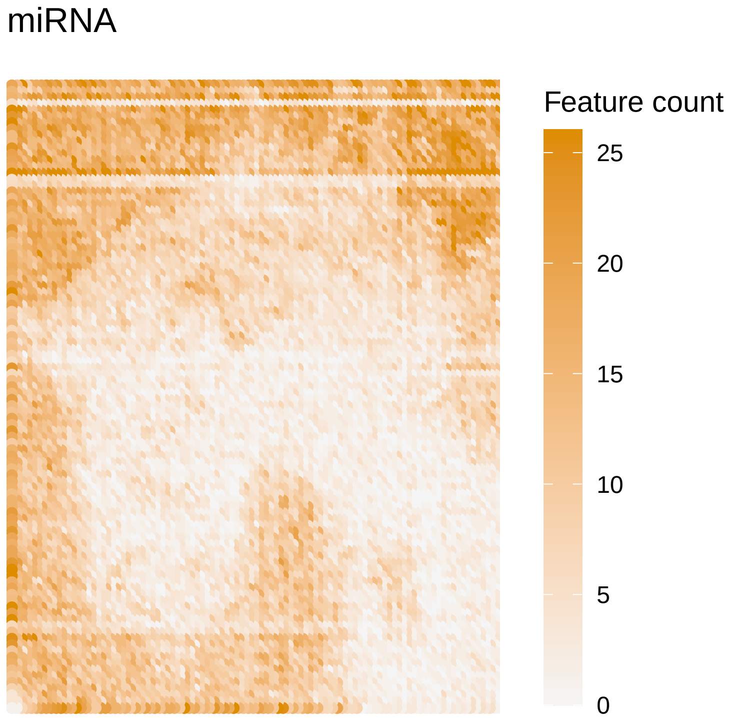

miRNA
Feature count
25
20
15
10
5
0

misc_RNA

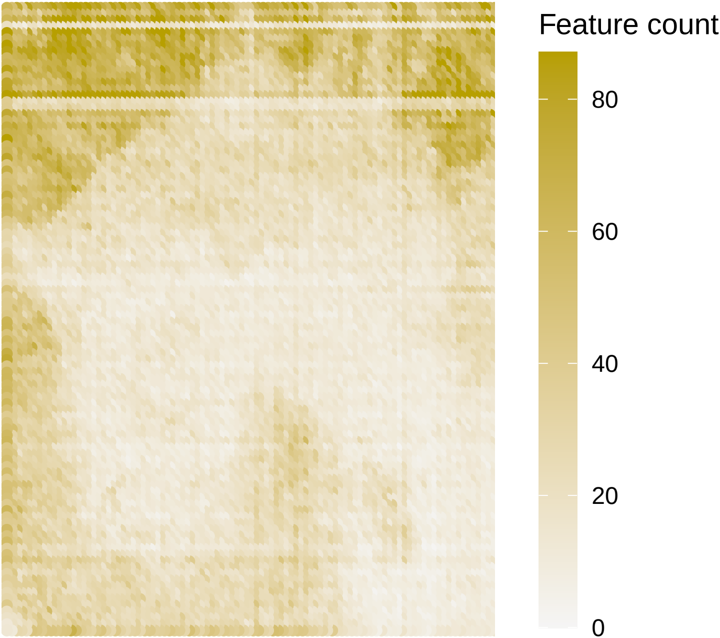

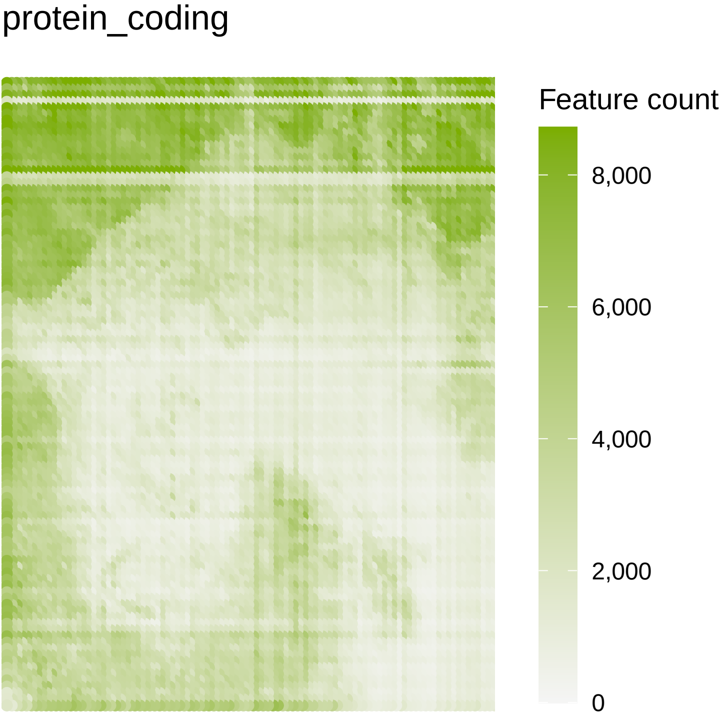
protein_coding
Feature count
8,000
6,000
4,000
2,000
0

pseudogene

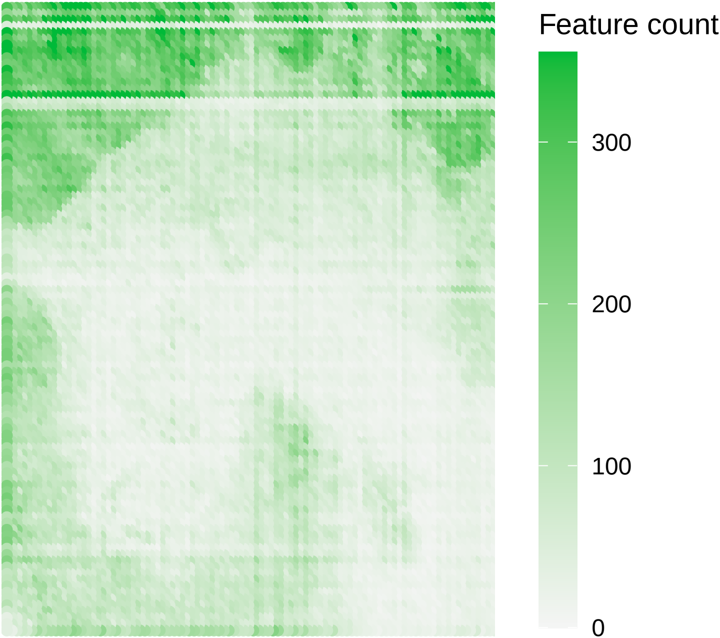

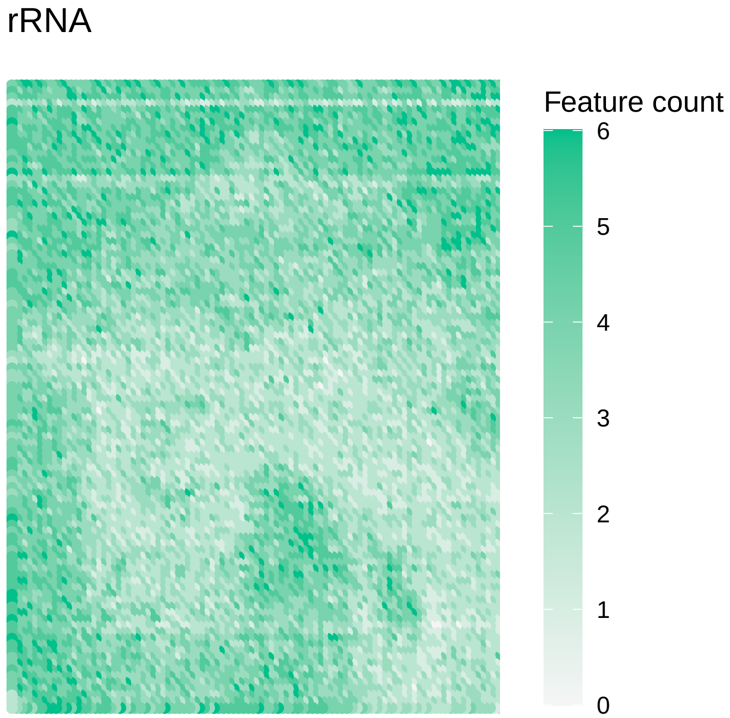
rRNA
Feature count
6
5
4
3
2
1
0

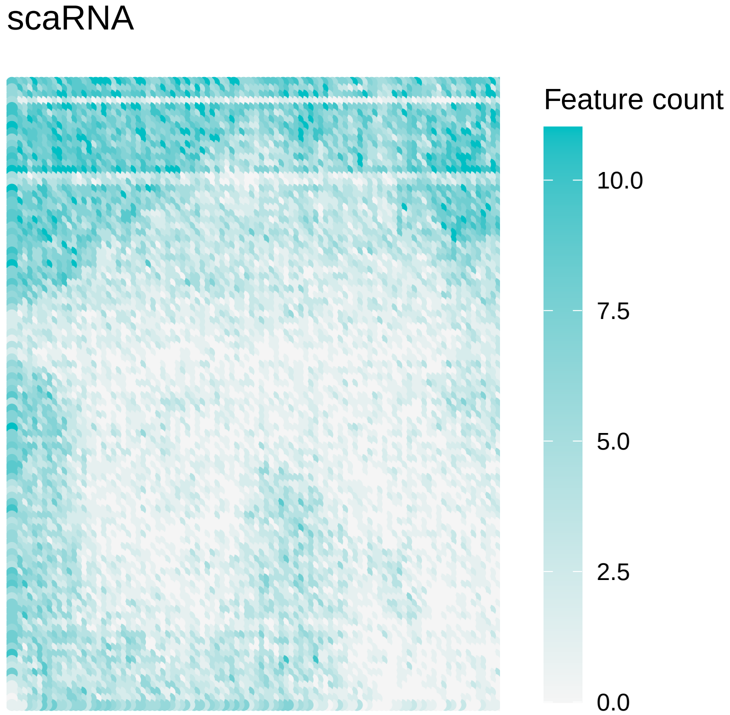
scaRNA
Feature count
10.0
7.5
5.0
2.5
0.0

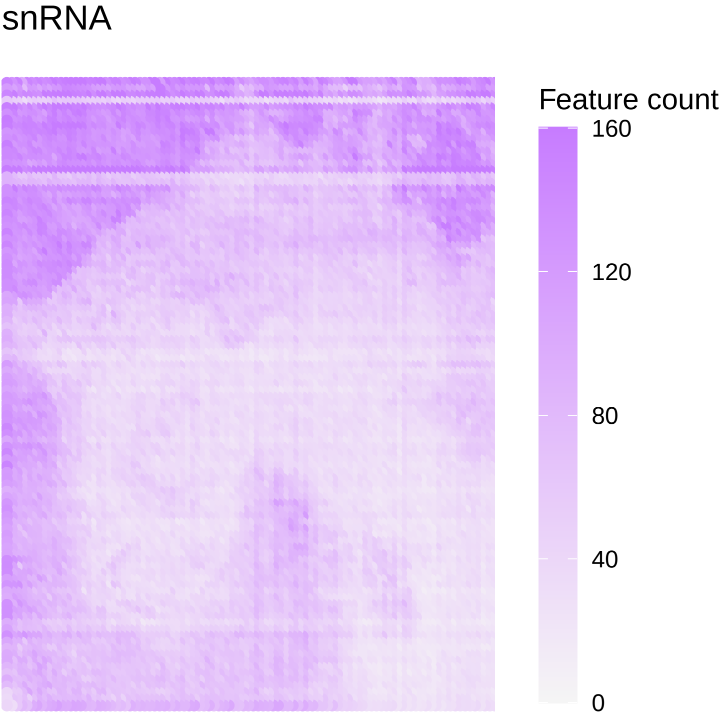
snRNA
Feature count
160
120
80
40
0

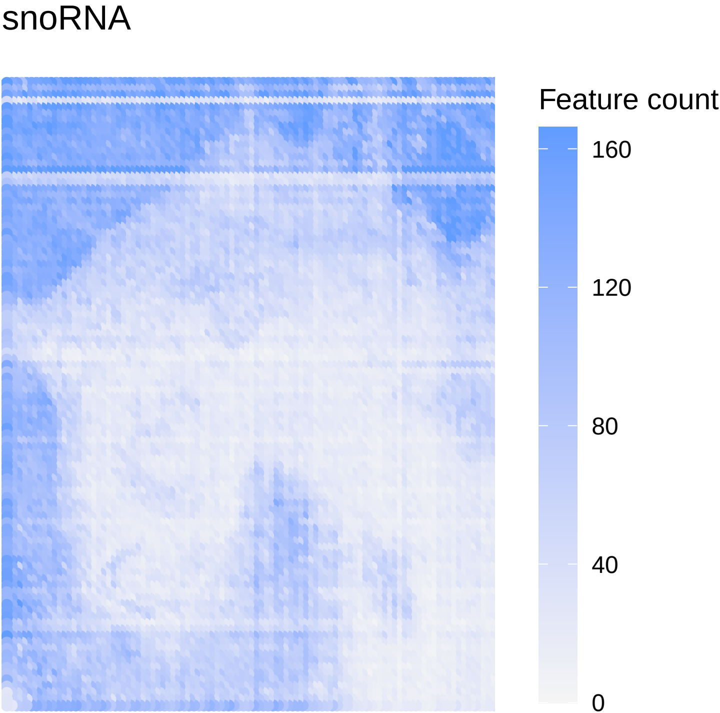
snoRNA
Feature count
160
120
80
40
0

tRNA

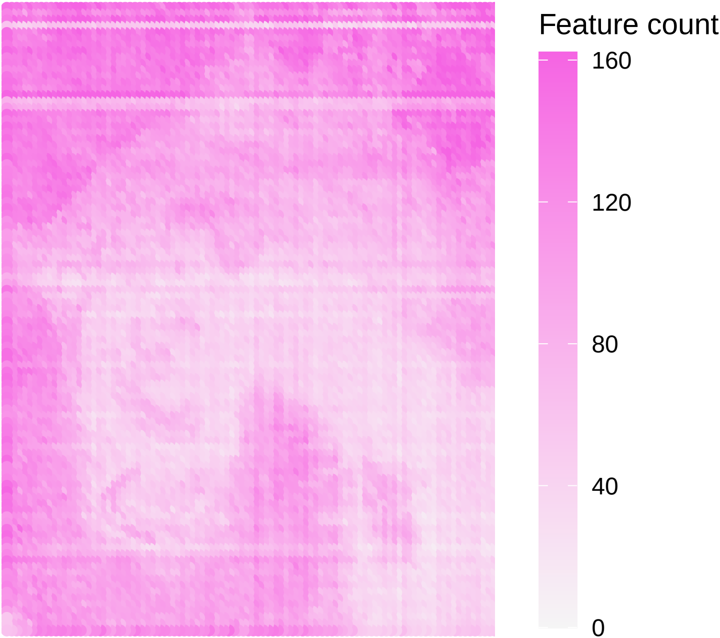

Supplement: btaf688_Supplementary_Data [file btaf688_supplementary_data.zip › Supplementary File 1.zip!/Supplementary File 1/Spatial_mapping/the_number_of_gene_feature/ASTRO/MALT.pdf]
