# Supplementary material for: ASTRO: Automated Spatial-Transcriptome whole RNA Output
Source: Bioinformatics. 2026 Jan 6;42(2):btaf688. doi: 10.1093/bioinformatics/btaf688 (PMC12866646; doi:10.1093/bioinformatics/btaf688)
Supplement: btaf688_Supplementary_Data [file btaf688_supplementary_data.zip › Supplementary File 1.zip!/Supplementary File 1/Spatial_mapping/the_number_of_gene_feature/ASTRO/Mouse Embryo 1.pdf]

Y_RNA

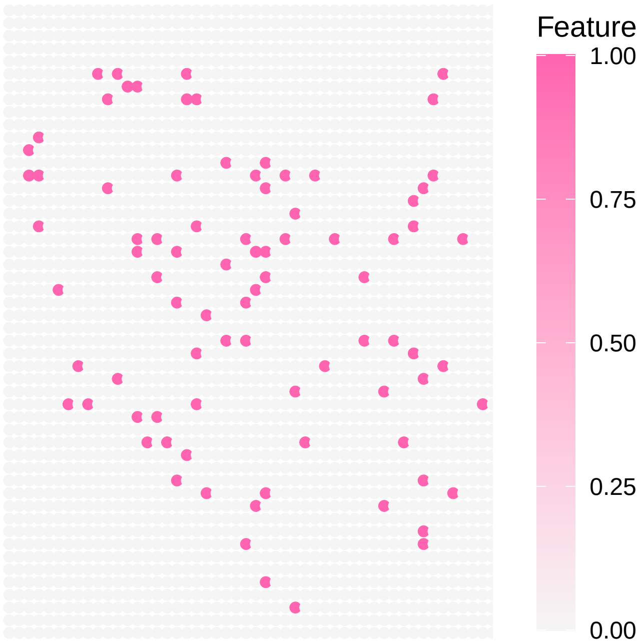

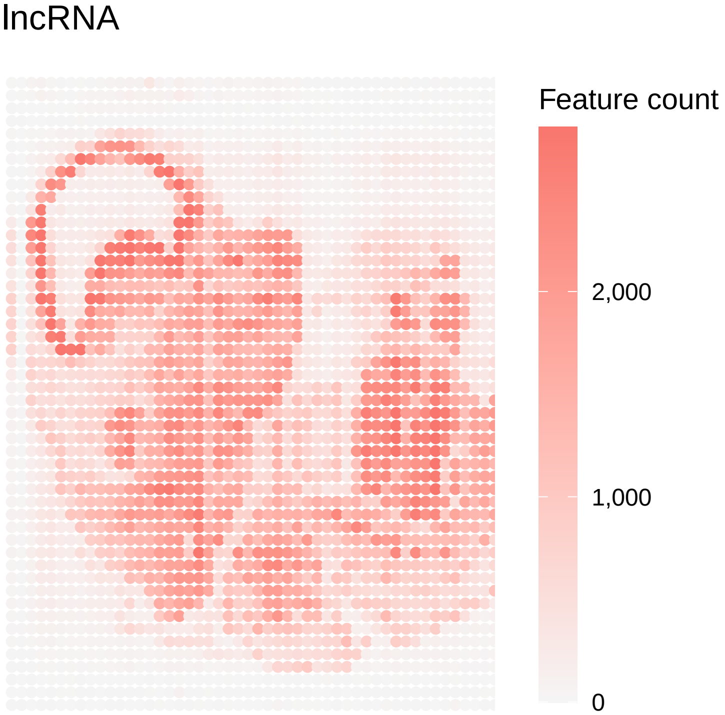
lncRNA
Feature count
2,000
1,000
0

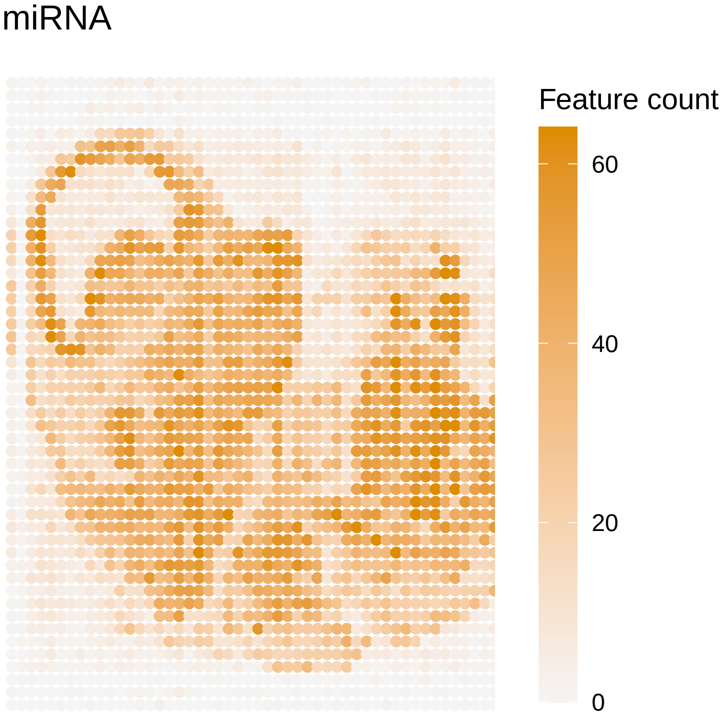
miRNA
Feature count
60
40
20
0

misc_RNA

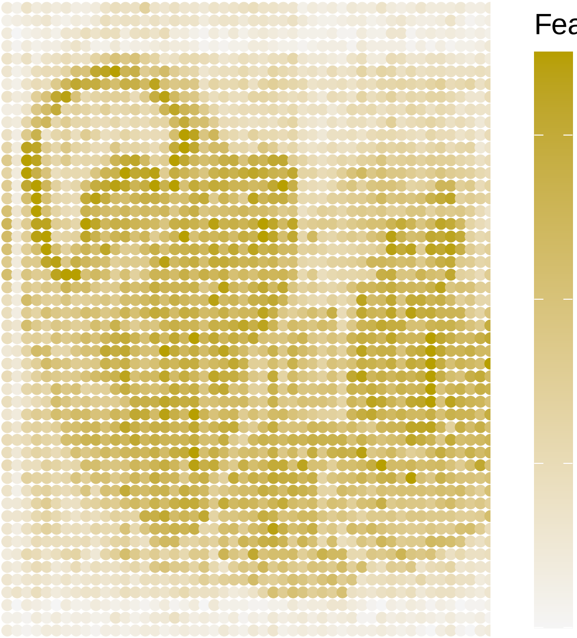

protein_coding

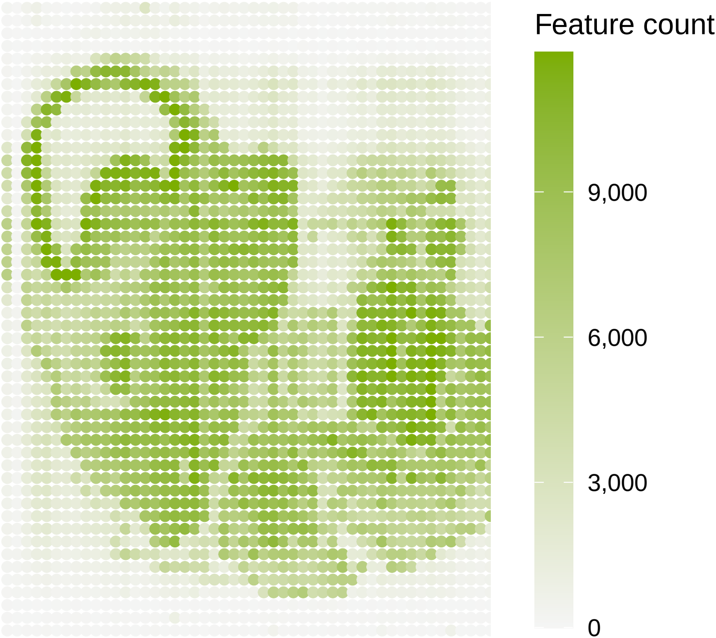

pseudogene

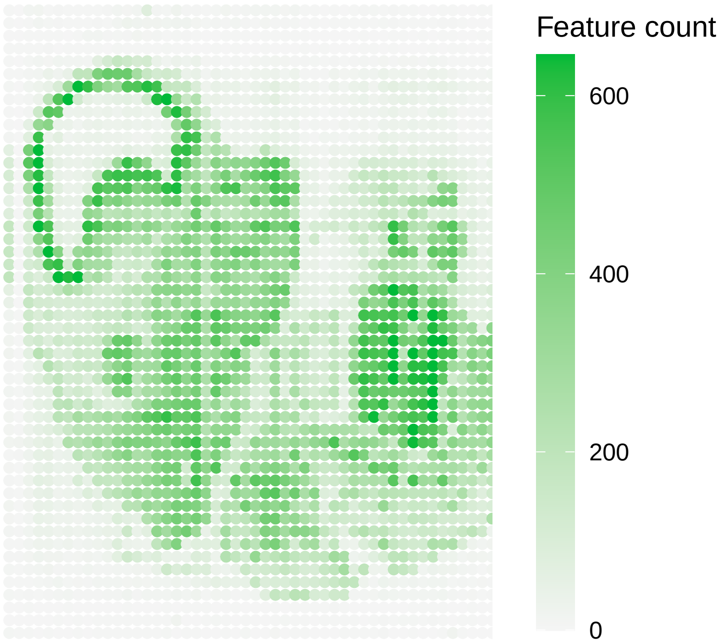

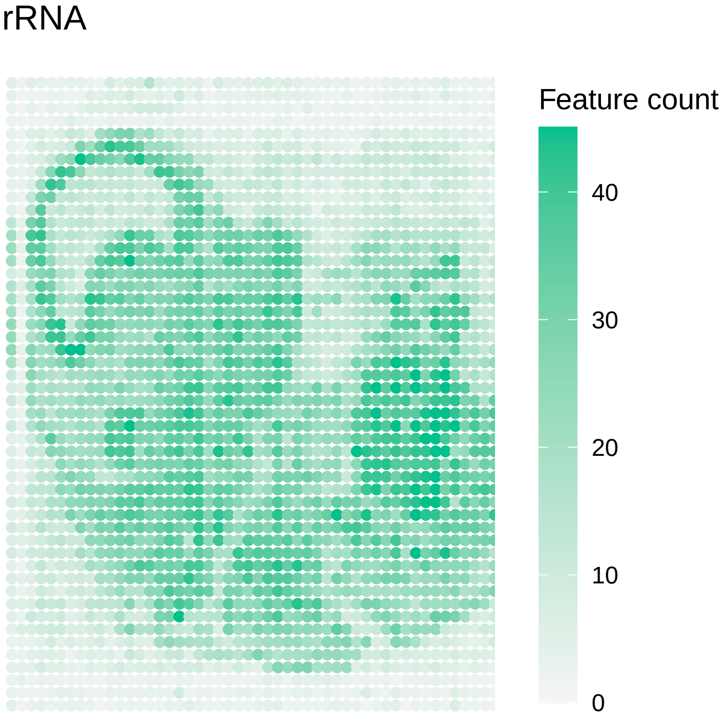
rRNA
Feature count
40
30
20
10
0

scaRNA

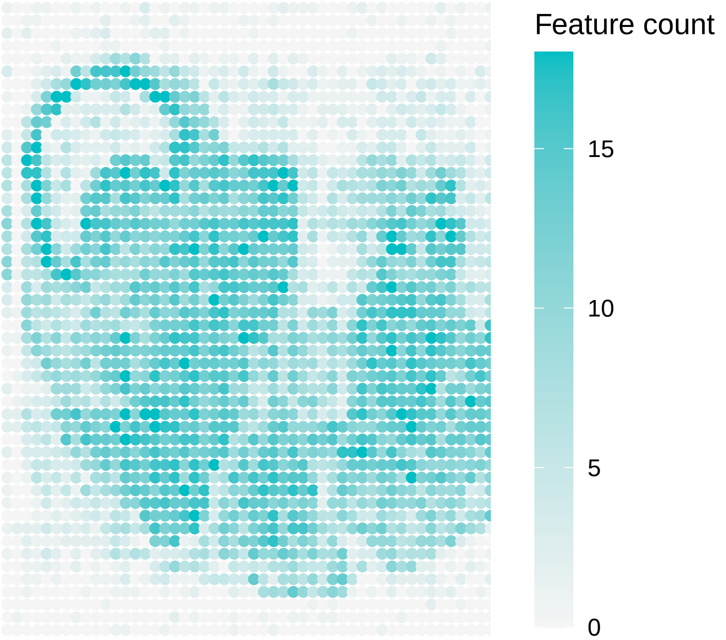

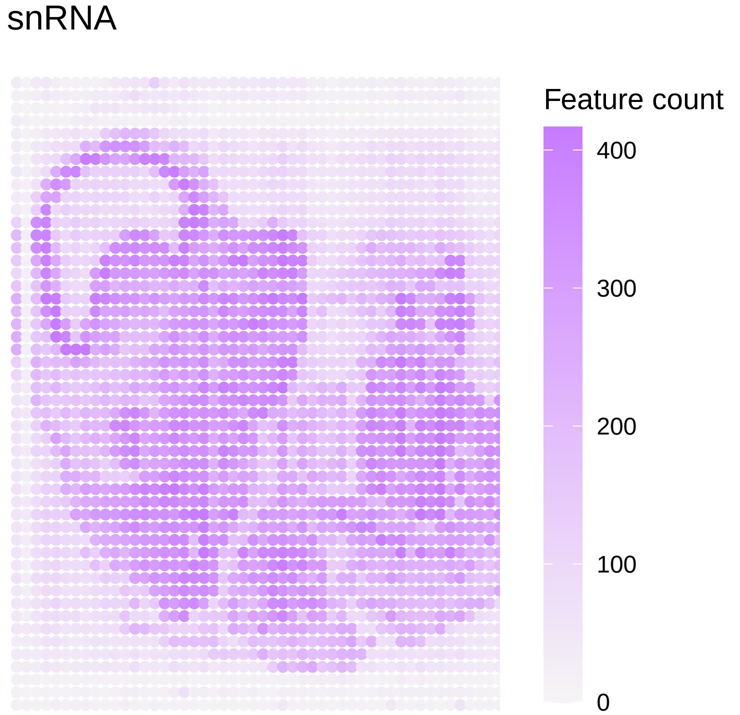
snRNA
Feature count
400
300
200
100
0

snoRNA

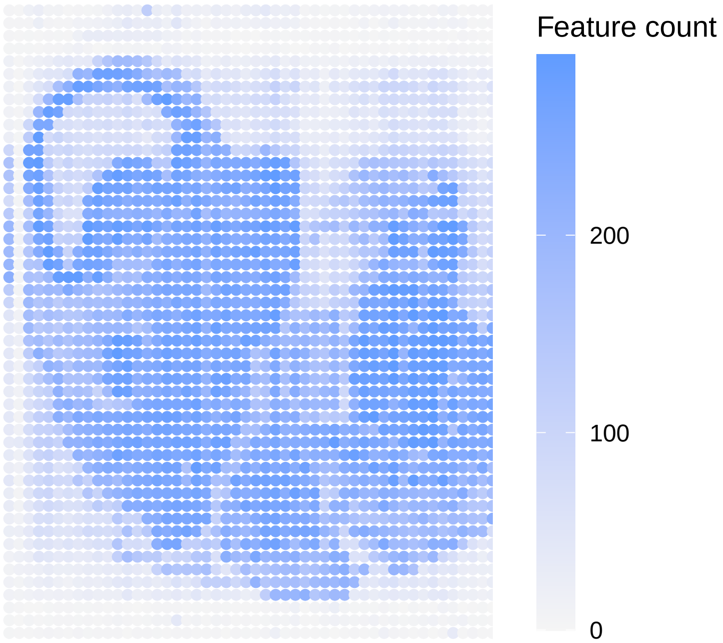

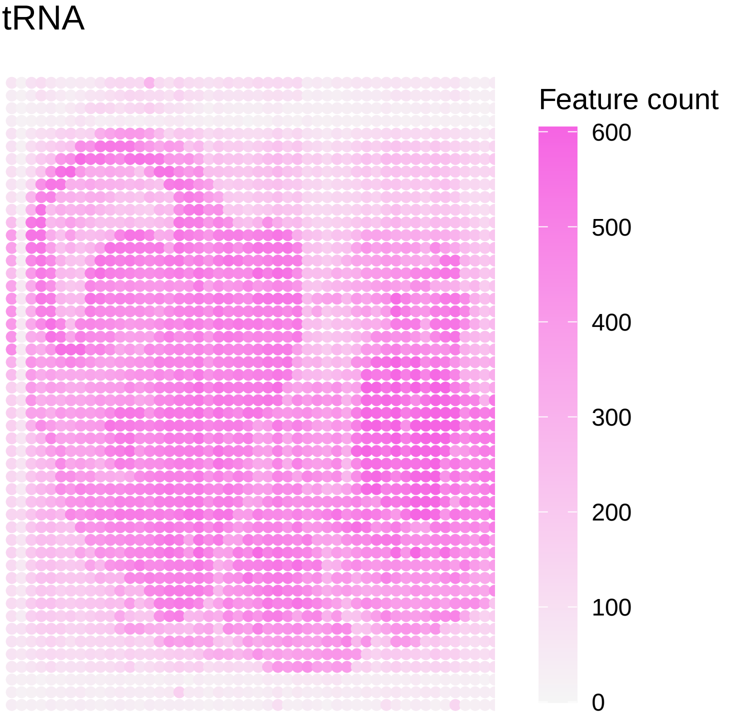
tRNA
Feature count
600
500
400
300
200
100
0
