# Supplementary material for: ASTRO: Automated Spatial-Transcriptome whole RNA Output
Source: Bioinformatics. 2026 Jan 6;42(2):btaf688. doi: 10.1093/bioinformatics/btaf688 (PMC12866646; doi:10.1093/bioinformatics/btaf688)

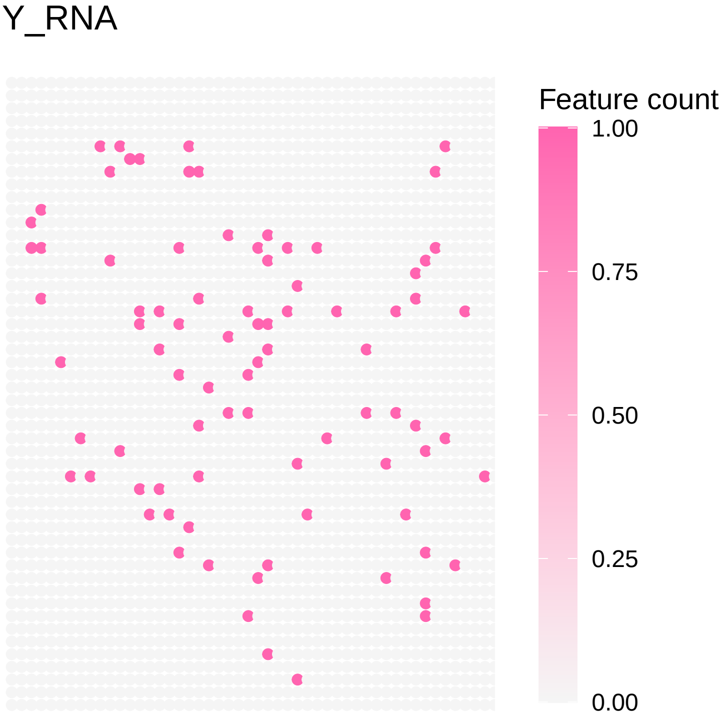
Y_RNA
Feature count
1.00
0.75
0.50
0.25
0.00

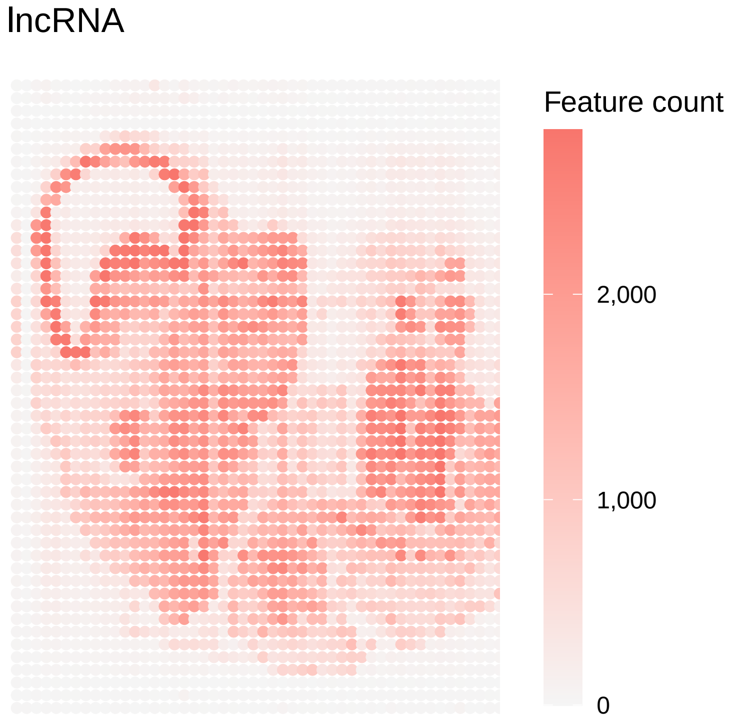
lncRNA
Feature count
2,000
1,000
0

miRNA

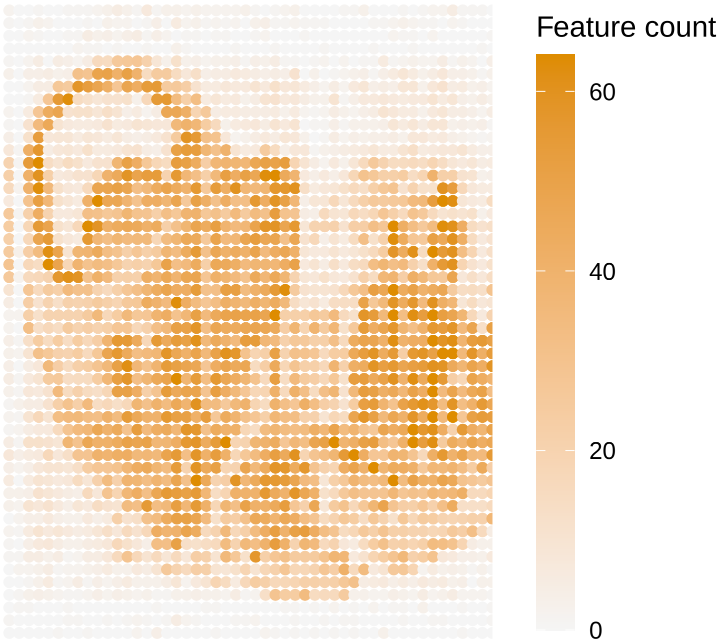

misc_RNA

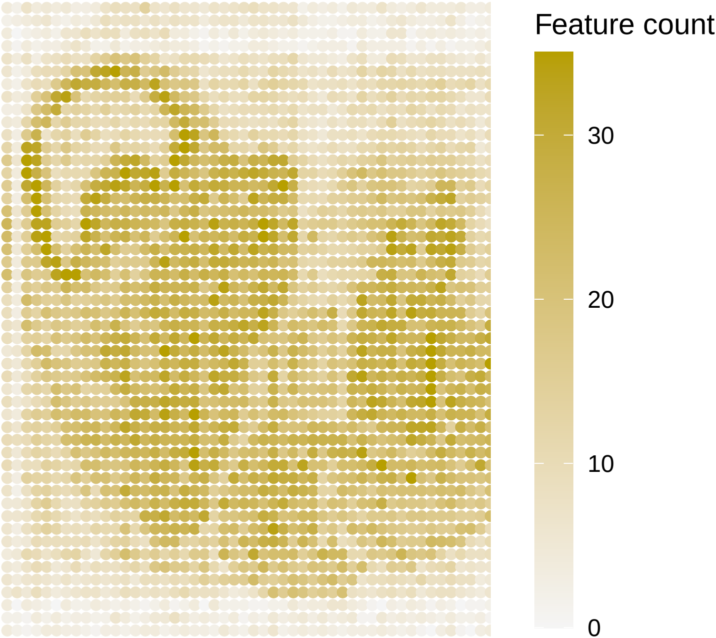

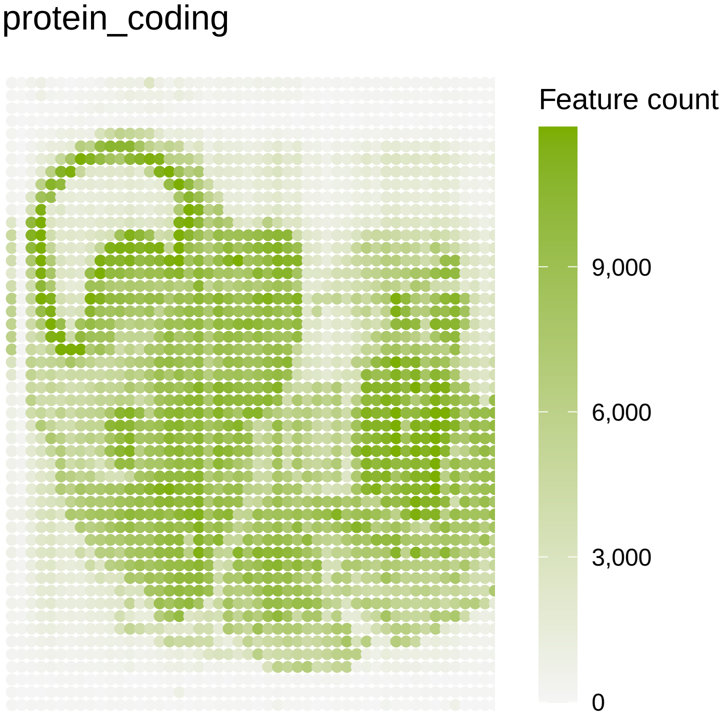
protein_coding
Feature count
9,000
6,000
3,000
0

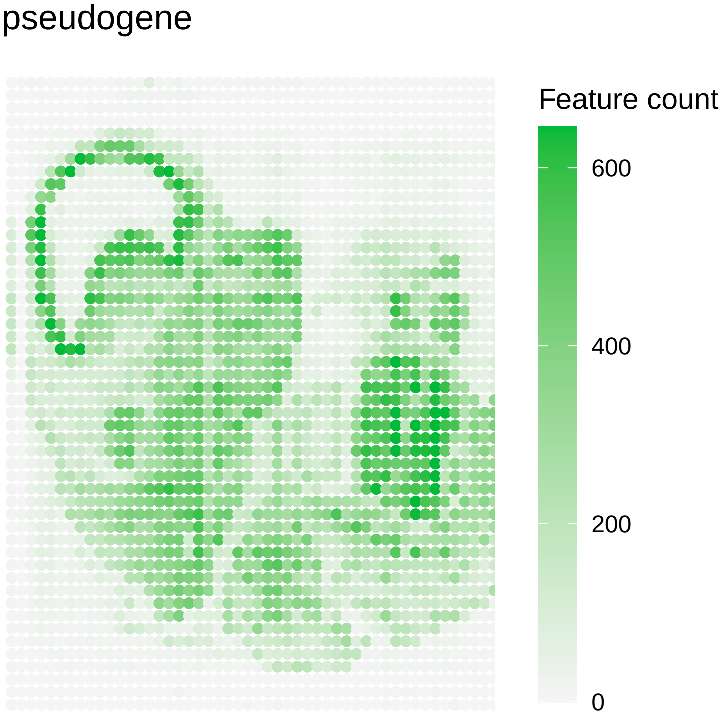
pseudogene
Feature count
600
400
200
0

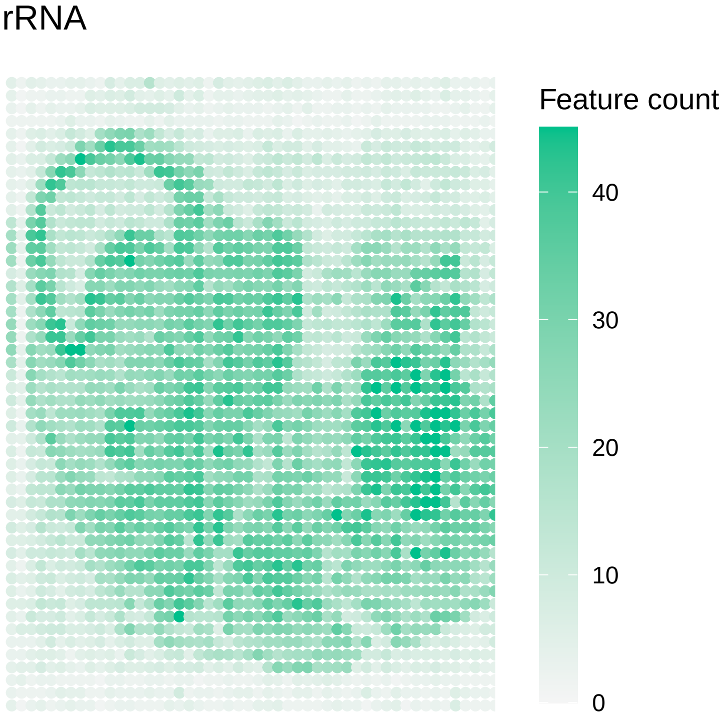
rRNA
Feature count
40
30
20
10
0

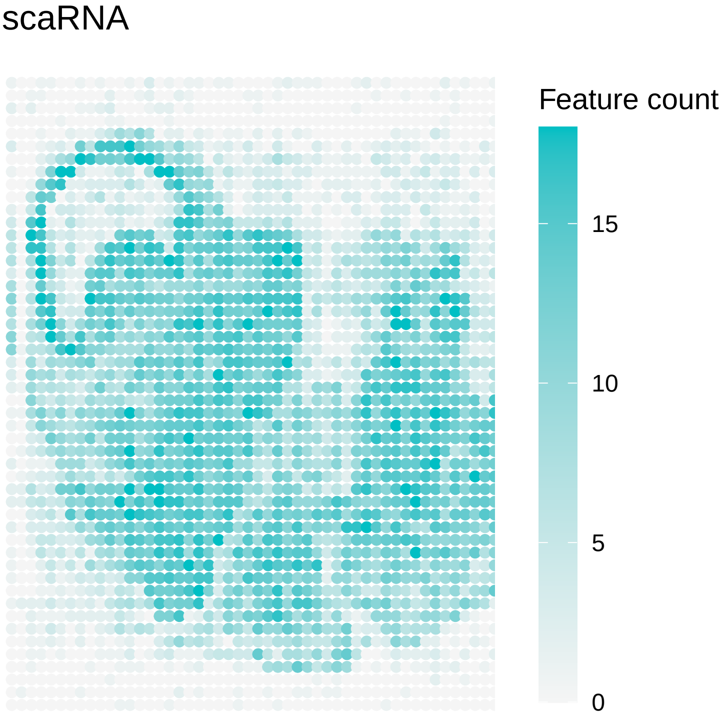
scaRNA
Feature count
15
10
5
0

snRNA

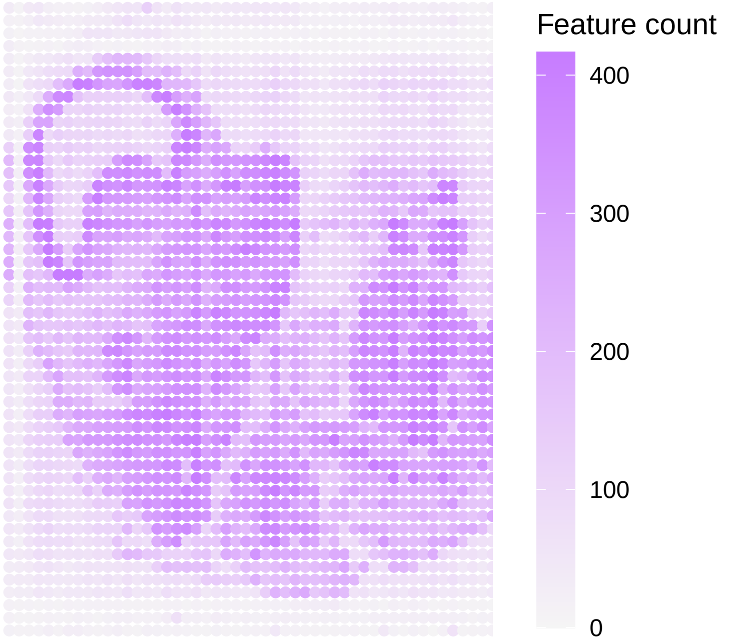

snoRNA

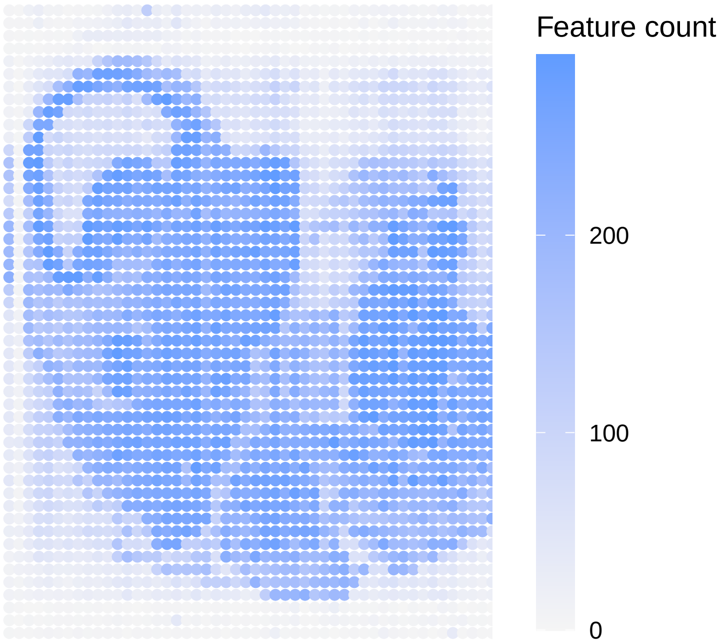

tRNA

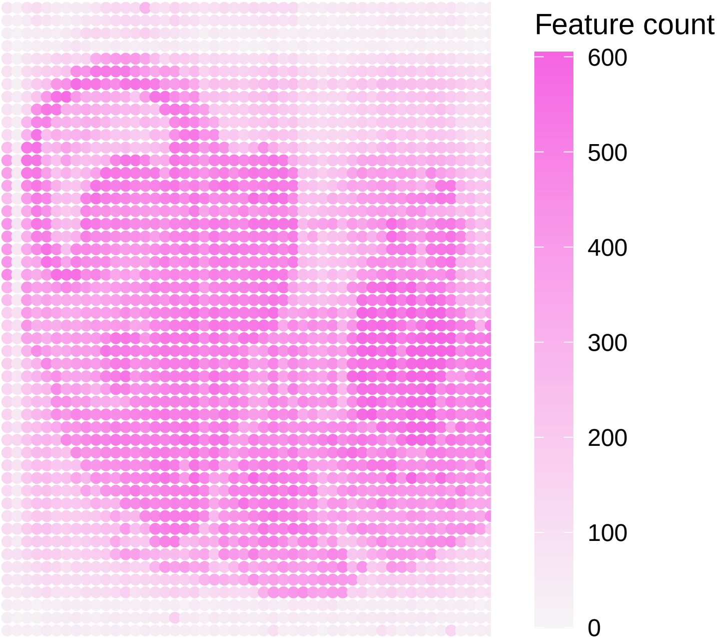

Supplement: btaf688_Supplementary_Data [file btaf688_supplementary_data.zip › Supplementary File 1.zip!/Supplementary File 1/Spatial_mapping/the_number_of_gene_feature/ASTRO/Mouse Embryo 2.pdf]
