# Supplementary material for: ASTRO: Automated Spatial-Transcriptome whole RNA Output
Source: Bioinformatics. 2026 Jan 6;42(2):btaf688. doi: 10.1093/bioinformatics/btaf688 (PMC12866646; doi:10.1093/bioinformatics/btaf688)
Supplement: btaf688_Supplementary_Data [file btaf688_supplementary_data.zip › Supplementary File 1.zip!/Supplementary File 1/Spatial_mapping/the_number_of_gene_feature/ASTRO/Healthy donor lymph node.pdf]

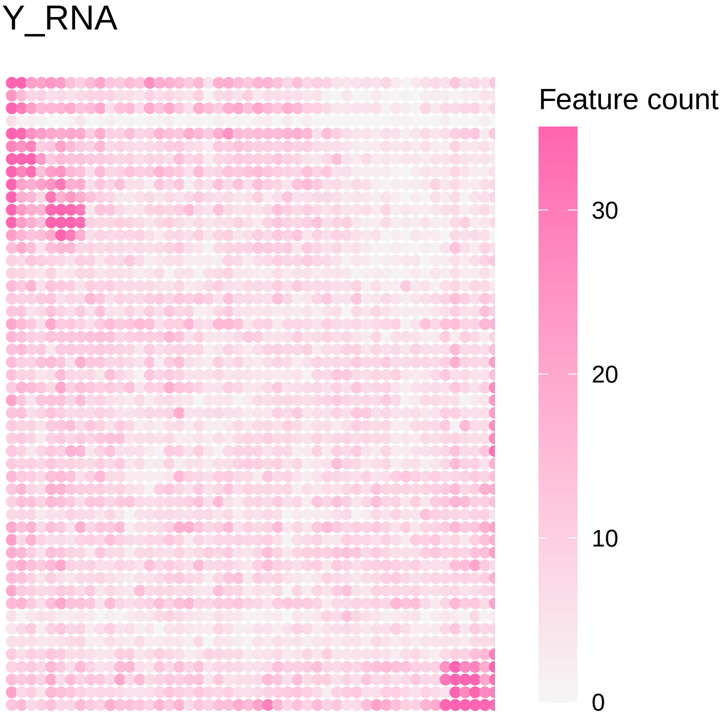
Y_RNA
Feature count
30
20
10
0

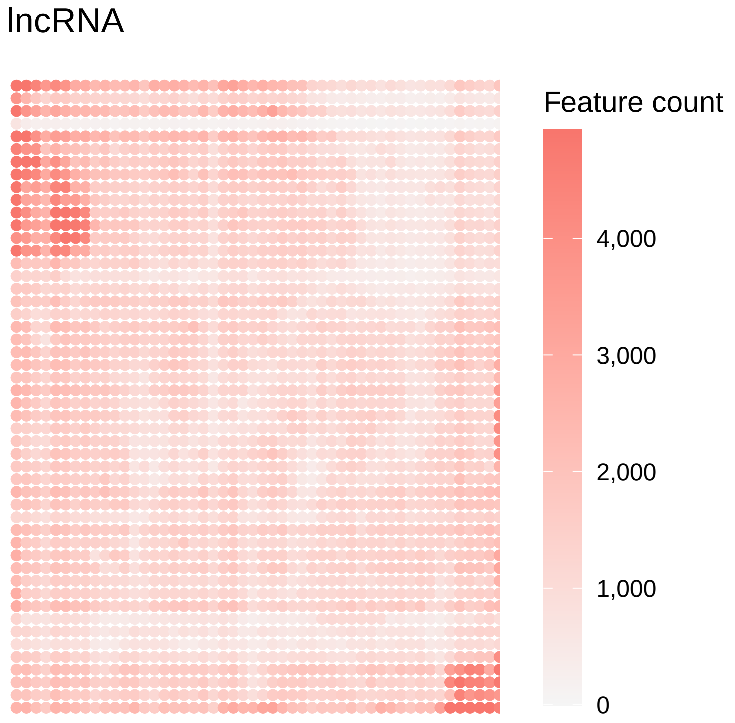
lncRNA
Feature count
4,000
3,000
2,000
1,000
0

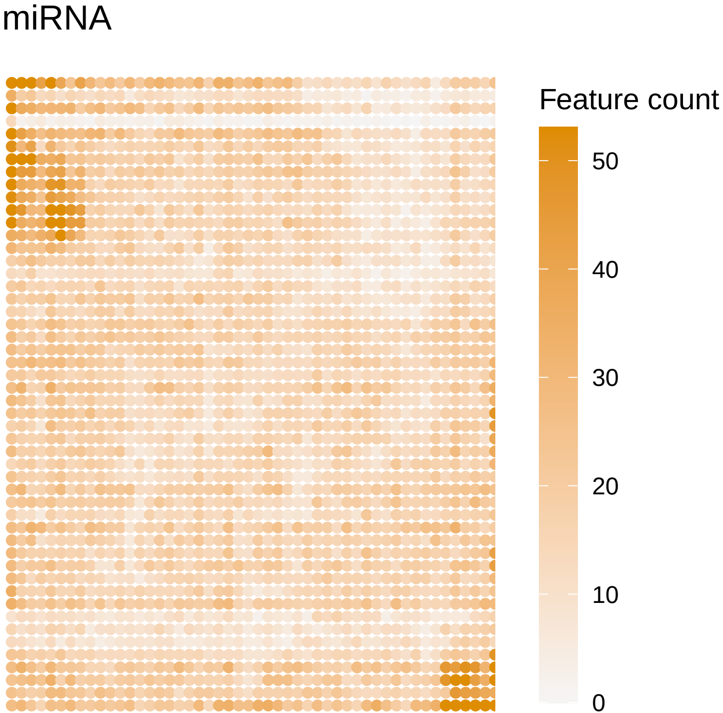
miRNA
Feature count
50
40
30
20
10
0

misc_RNA

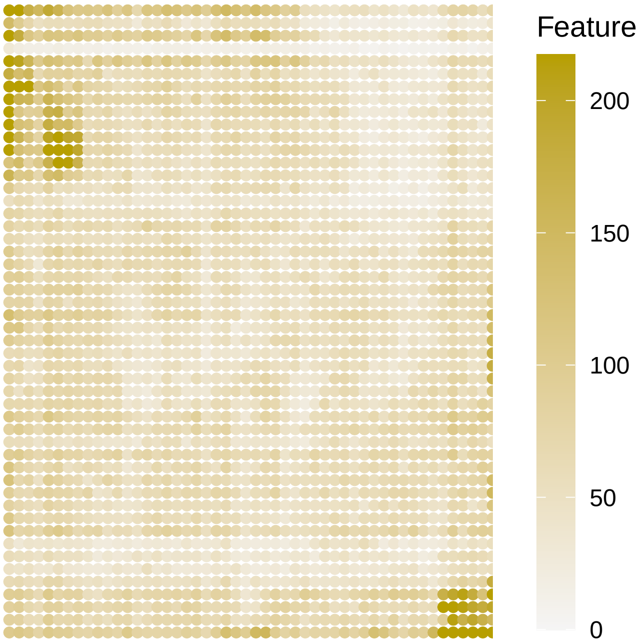

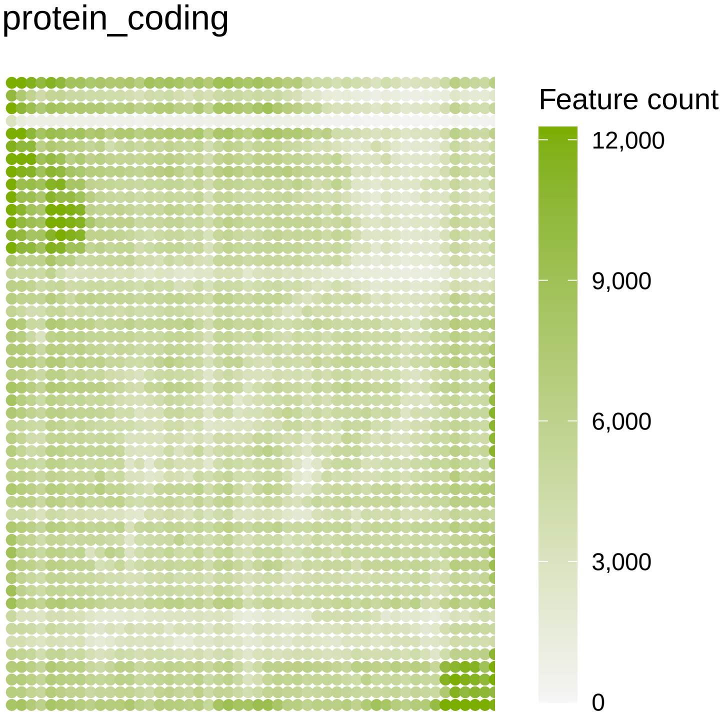
protein_coding
Feature count
12,000
9,000
6,000
3,000
0

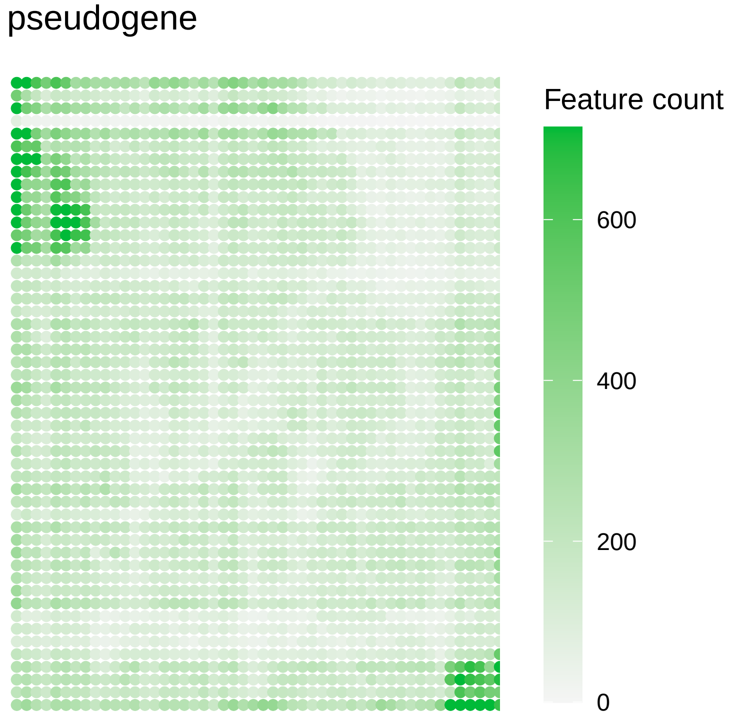
pseudogene
Feature count
600
400
200
0

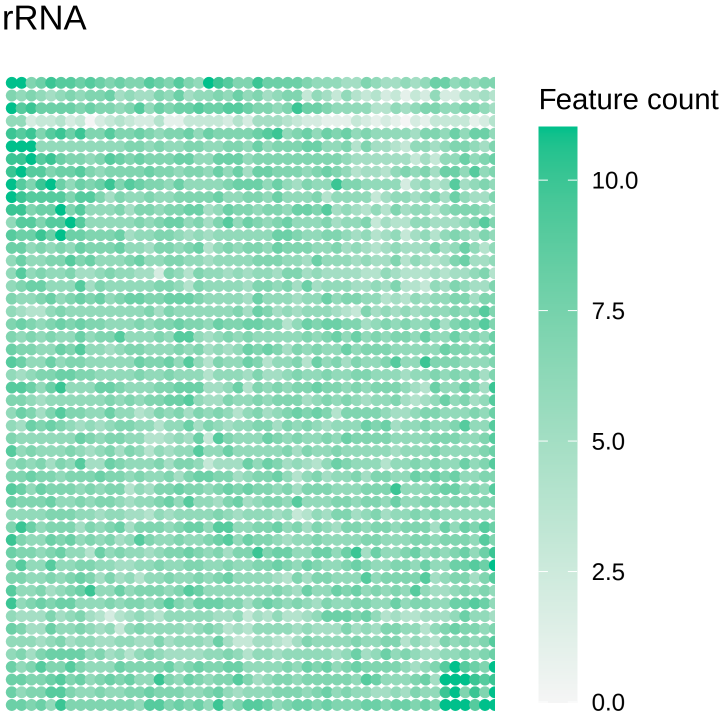
rRNA
Feature count
10.0
7.5
5.0
2.5
0.0

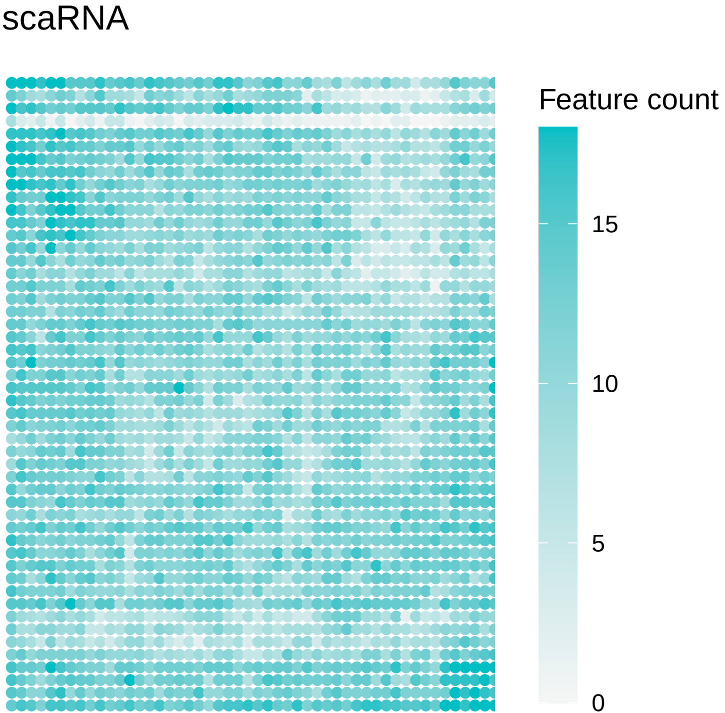
scaRNA
Feature count
15
10
5
0

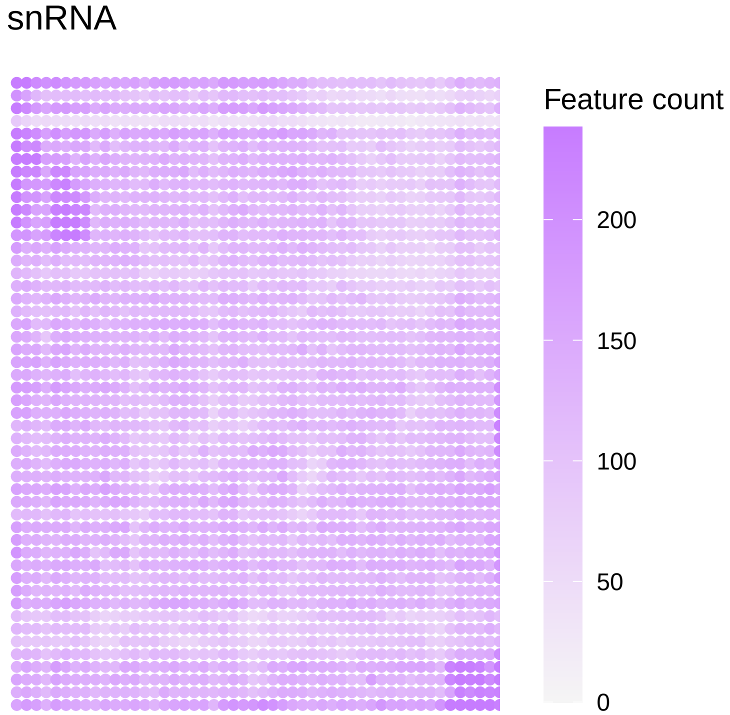
snRNA
Feature count
200
150
100
50
0

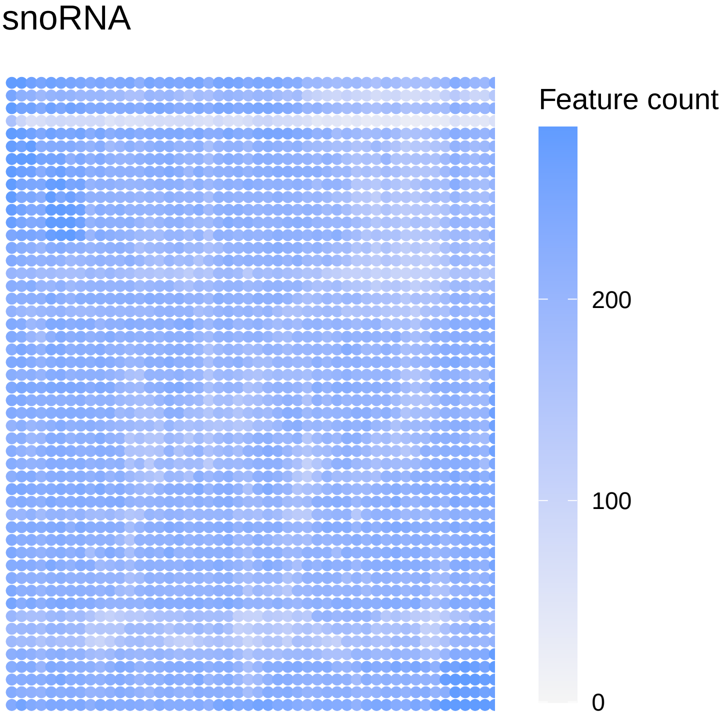
snoRNA
Feature count
200
100
0

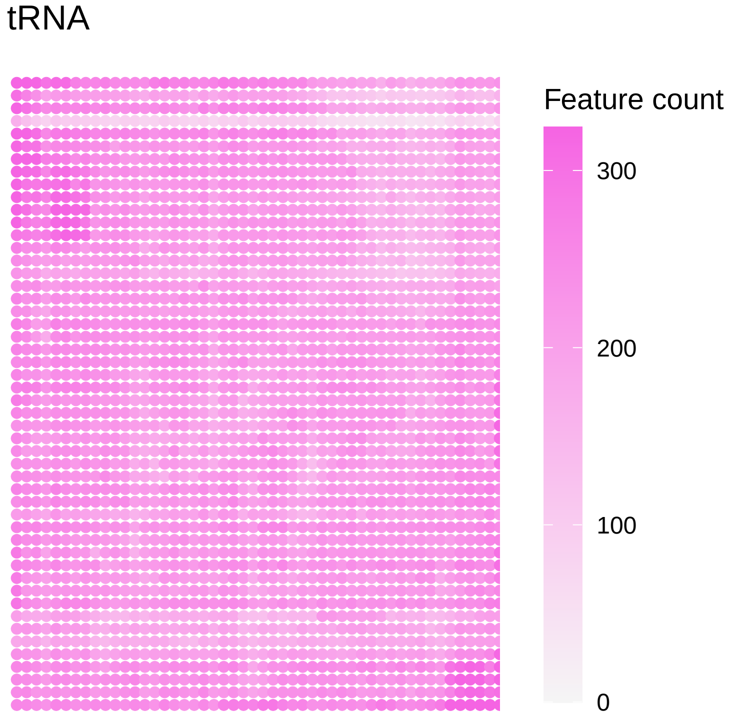
tRNA
Feature count
300
200
100
0
