# Supplementary material for: ASTRO: Automated Spatial-Transcriptome whole RNA Output
Source: Bioinformatics. 2026 Jan 6;42(2):btaf688. doi: 10.1093/bioinformatics/btaf688 (PMC12866646; doi:10.1093/bioinformatics/btaf688)
Supplement: btaf688_Supplementary_Data [file btaf688_supplementary_data.zip › Supplementary File 1.zip!/Supplementary File 1/Spatial_mapping/the_number_of_UMI/ST-pipeline/MALT.pdf]

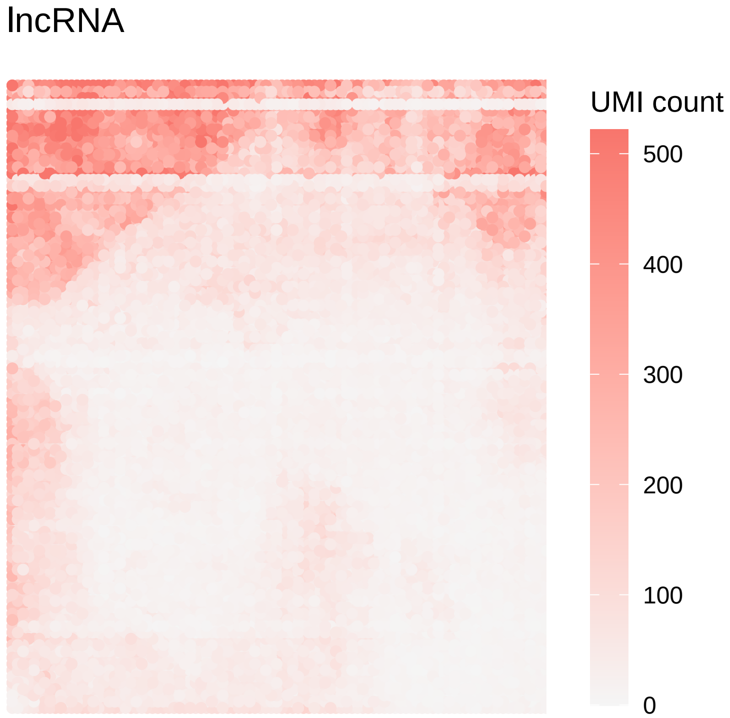

lncRNA
UMI count
500
400
300
200
100
0

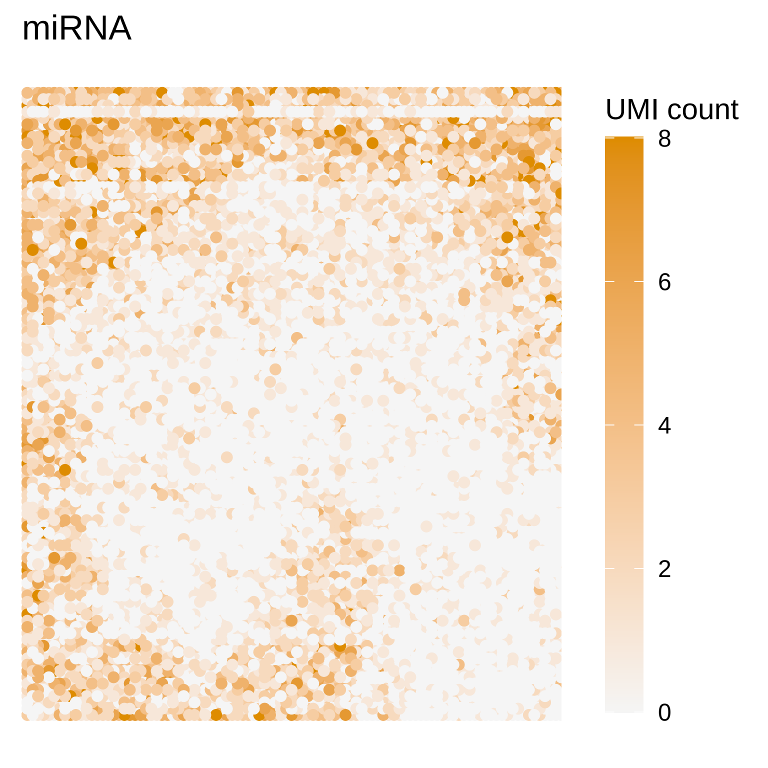
miRNA
UMI count
8
6
4
2
0

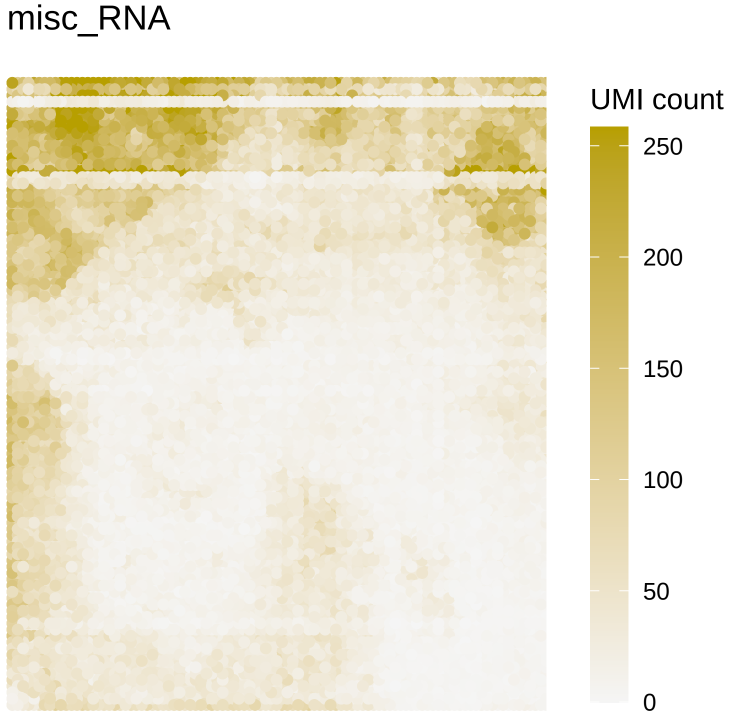
misc_RNA
UMI count
250
200
150
100
50
0

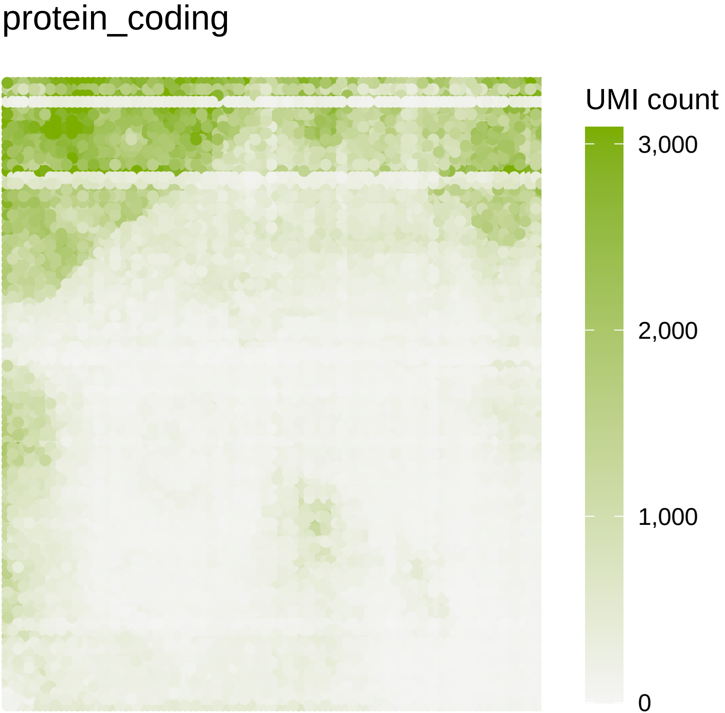
protein_coding
UMI count
3,000
2,000
1,000
0

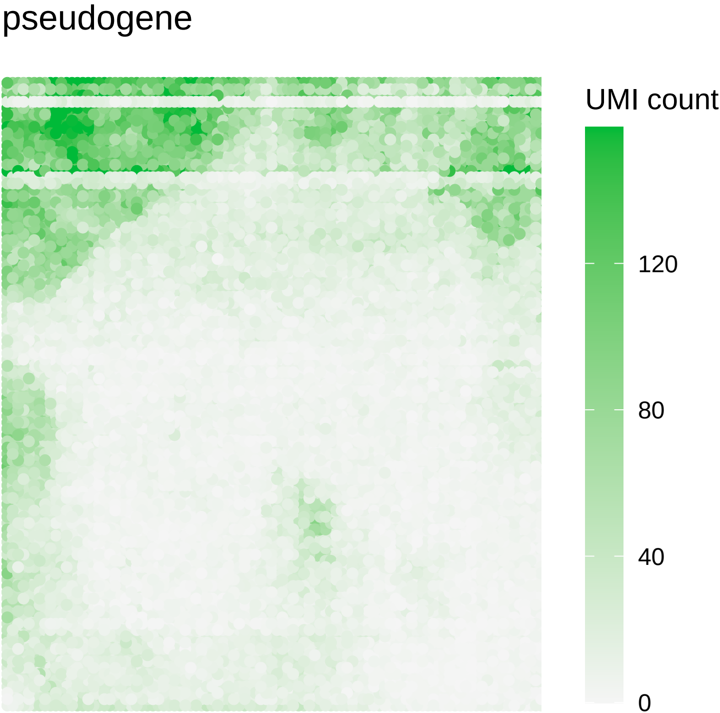

pseudogene
UMI count
120
80
40
0

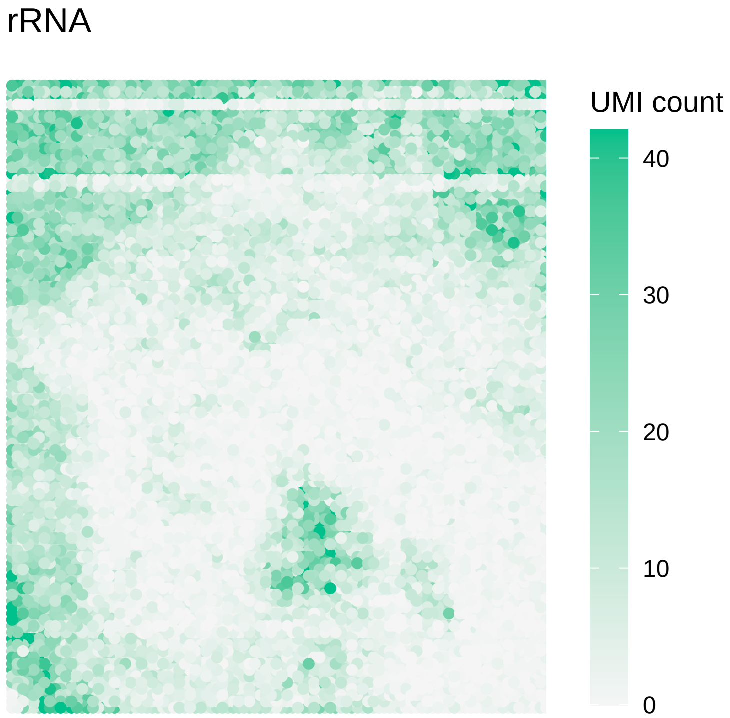
rRNA
UMI count
40
30
20
10
0

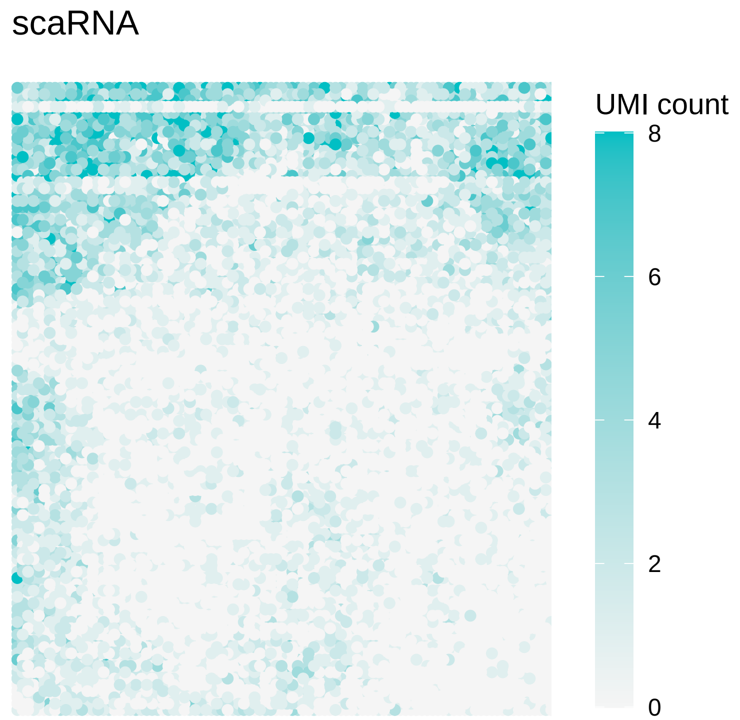
scaRNA
UMI count
8
6
4
2
0

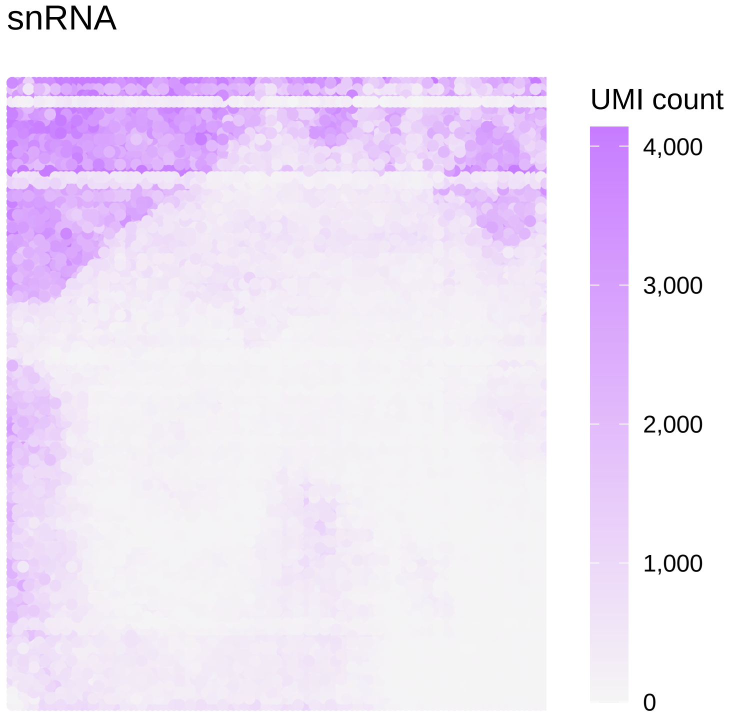

snRNA
UMI count
4,000
3,000
2,000
1,000
0

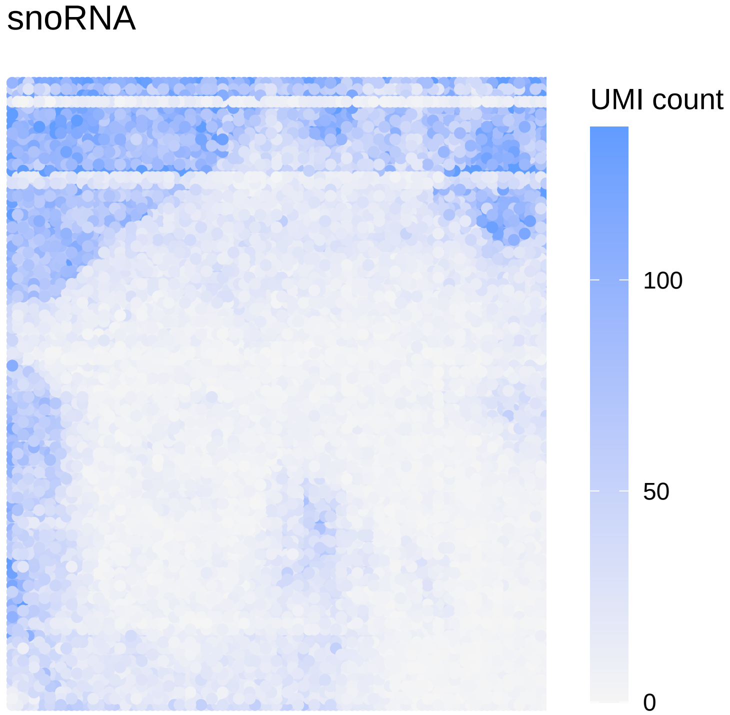
snoRNA
UMI count
100
50
0
