# Supplementary material for: ASTRO: Automated Spatial-Transcriptome whole RNA Output
Source: Bioinformatics. 2026 Jan 6;42(2):btaf688. doi: 10.1093/bioinformatics/btaf688 (PMC12866646; doi:10.1093/bioinformatics/btaf688)
Supplement: btaf688_Supplementary_Data [file btaf688_supplementary_data.zip › Supplementary File 1.zip!/Supplementary File 1/Spatial_mapping/the_number_of_UMI/ST-pipeline/Mouse Embryo 1.pdf]

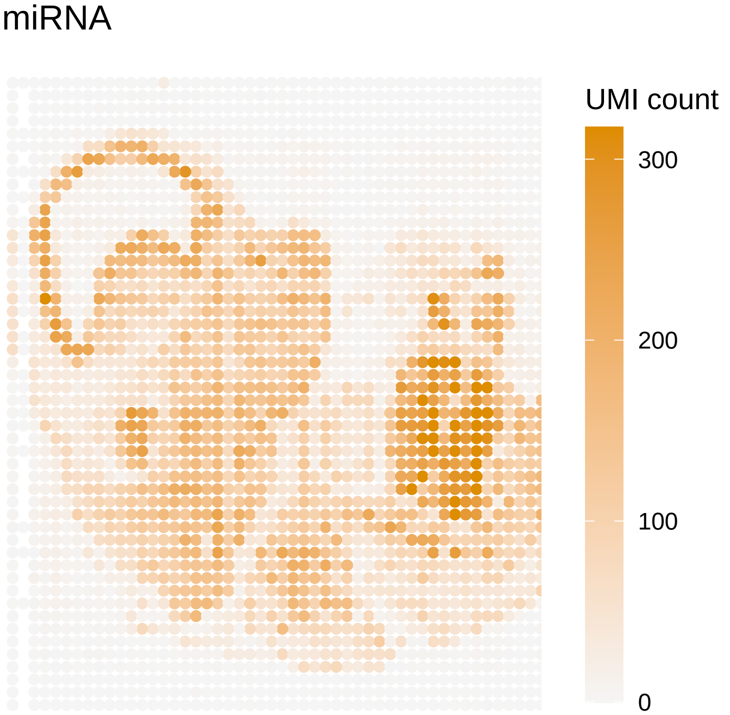

miRNA
UMI count
300
200
100
0

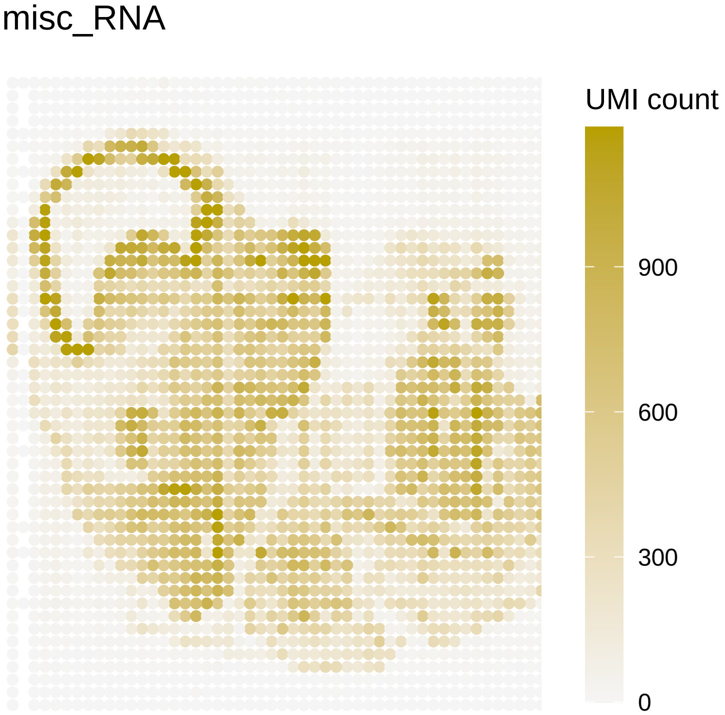
misc_RNA
UMI count
900
600
300
0

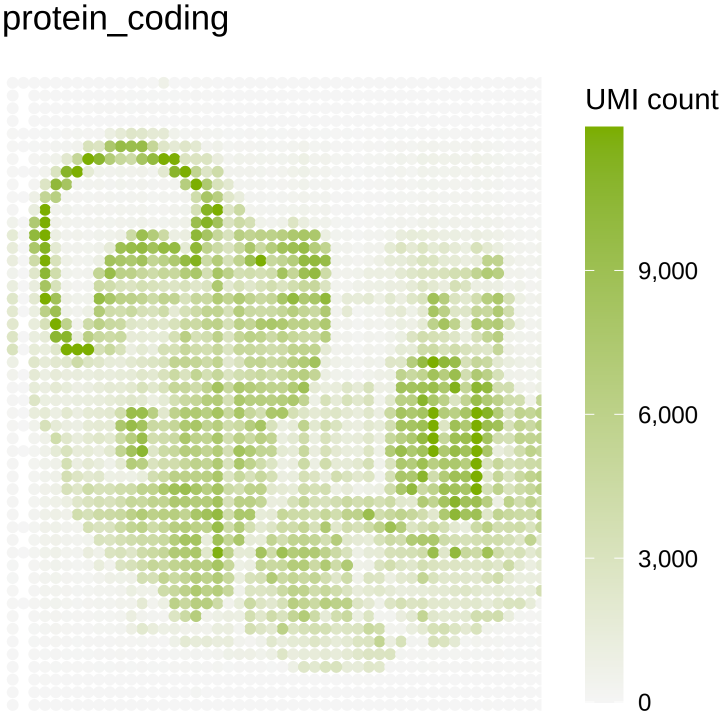
protein_coding
UMI count
9,000
6,000
3,000
0

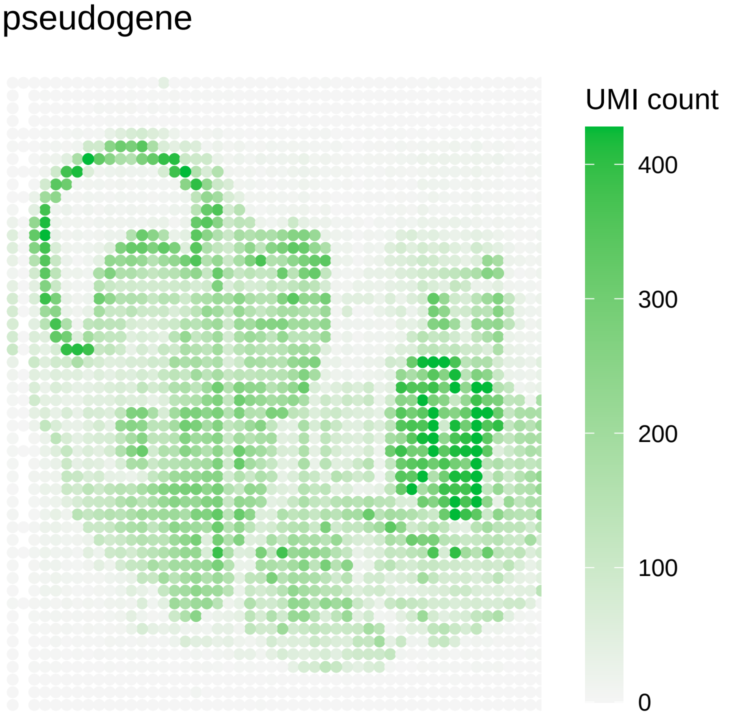
pseudogene
UMI count
400
300
200
100
0

rRNA

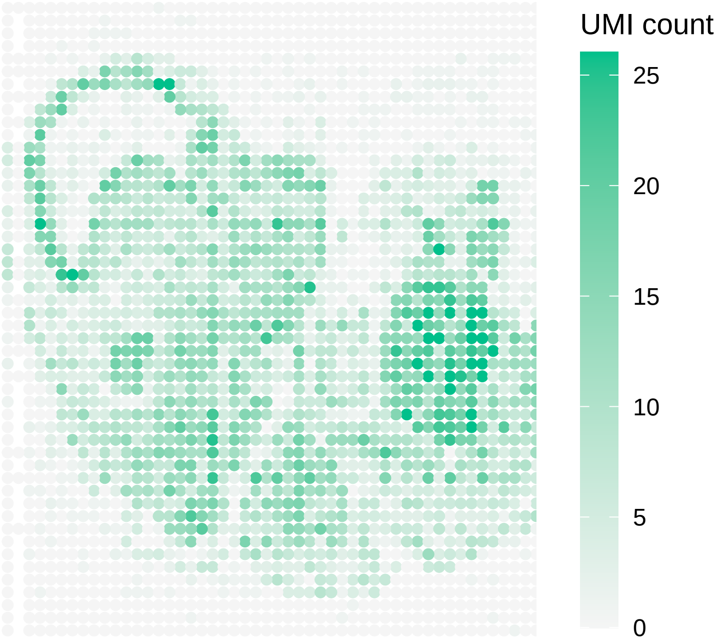

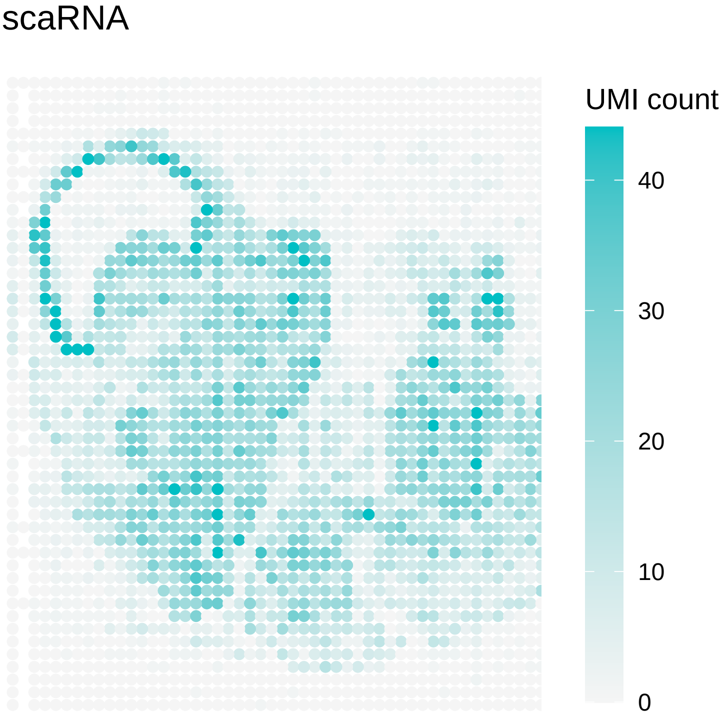
scaRNA
UMI count
40
30
20
10
0

snRNA

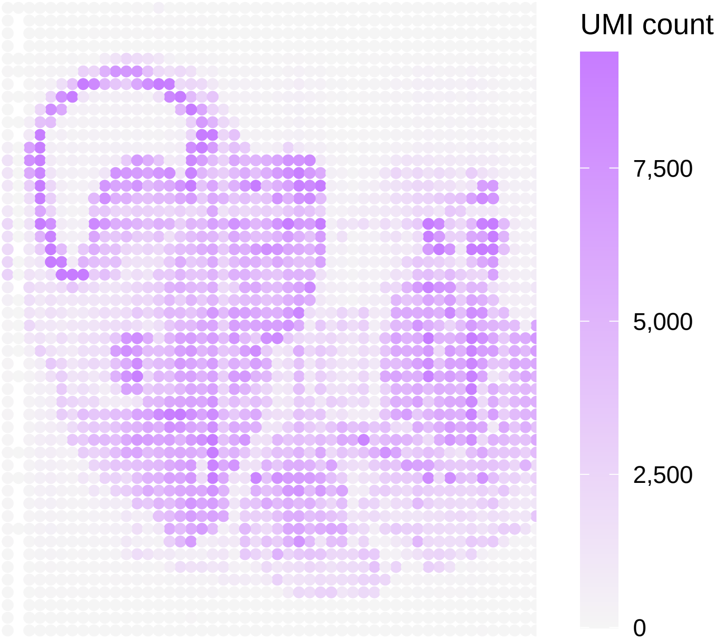

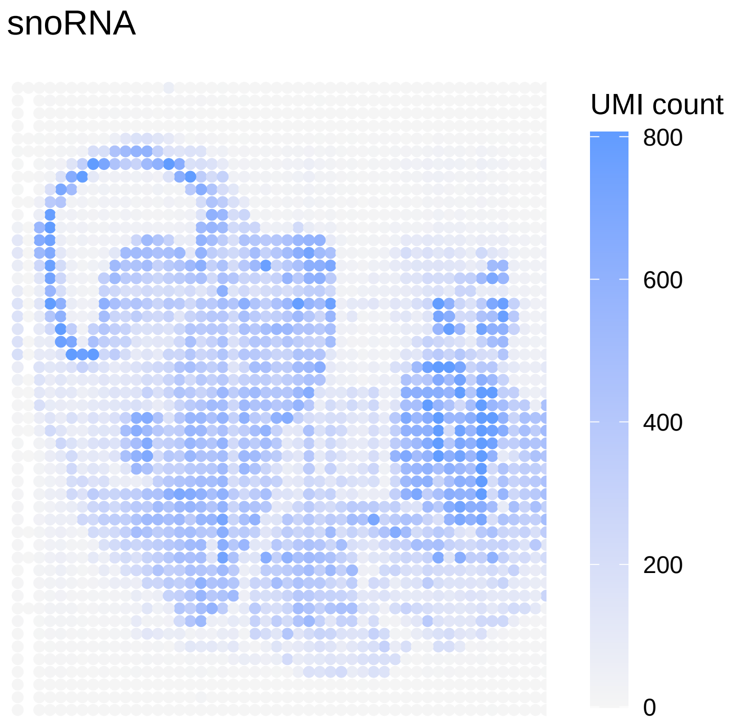
snoRNA
UMI count
800
600
400
200
0
