# Supplementary material for: ASTRO: Automated Spatial-Transcriptome whole RNA Output
Source: Bioinformatics. 2026 Jan 6;42(2):btaf688. doi: 10.1093/bioinformatics/btaf688 (PMC12866646; doi:10.1093/bioinformatics/btaf688)
Supplement: btaf688_Supplementary_Data [file btaf688_supplementary_data.zip › Supplementary File 1.zip!/Supplementary File 1/Spatial_mapping/the_number_of_UMI/ST-pipeline/Mouse Embryo 2.pdf]

miRNA

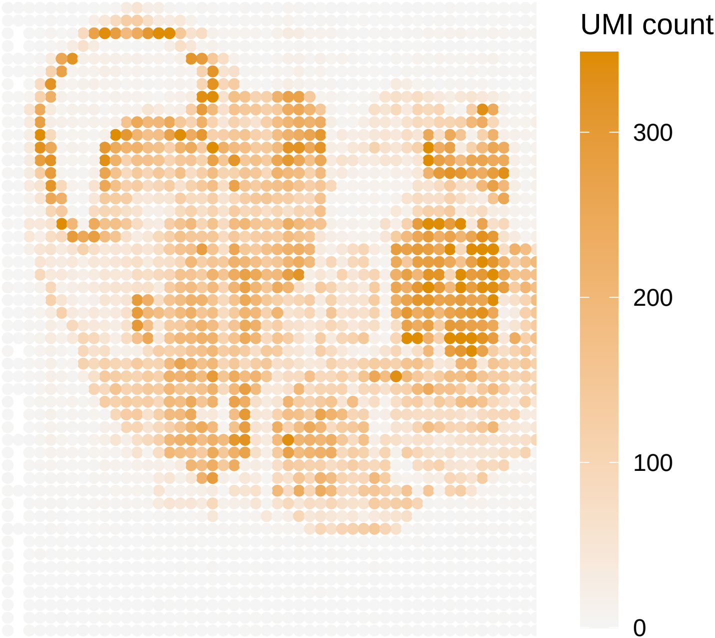

misc_RNA

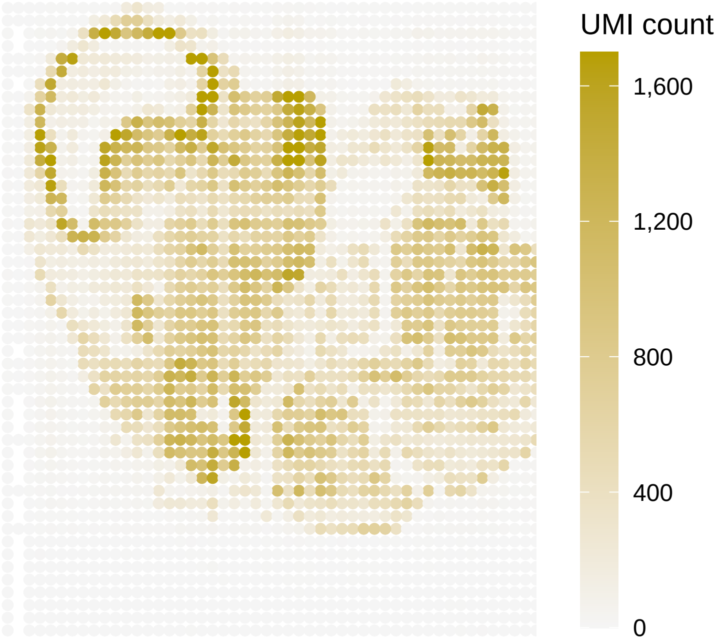

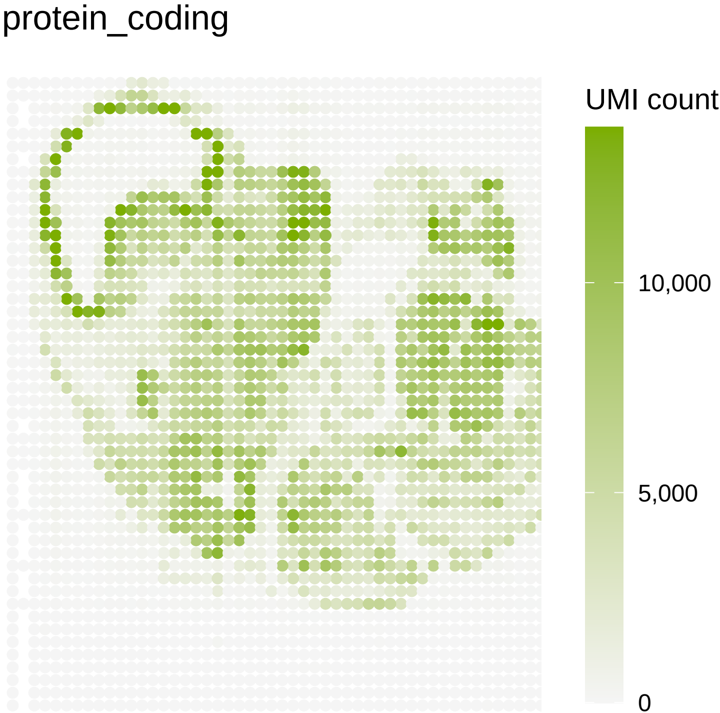
protein_coding
UMI count
10,000
5,000
0

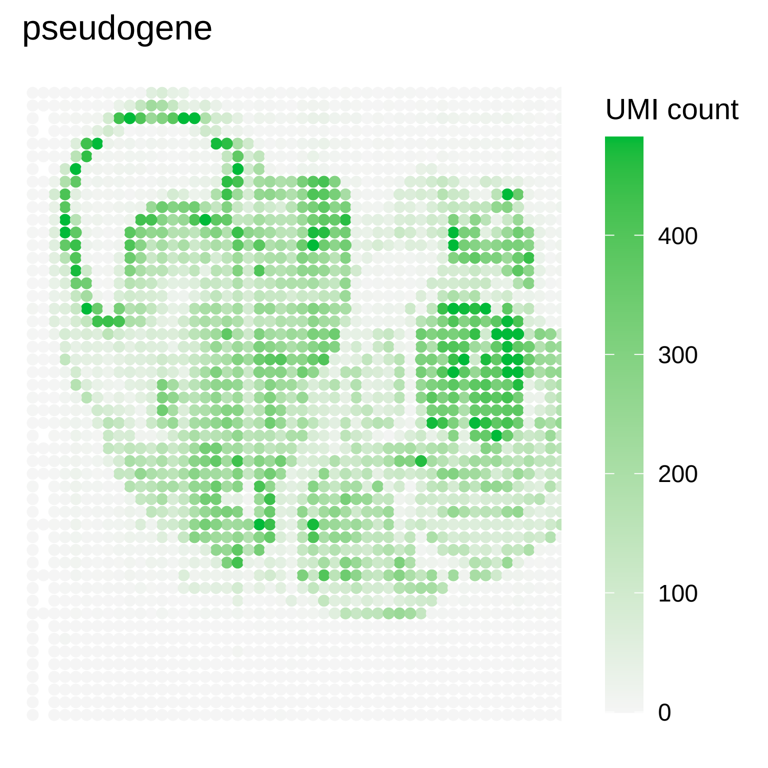
pseudogene
UMI count
400
300
200
100
0

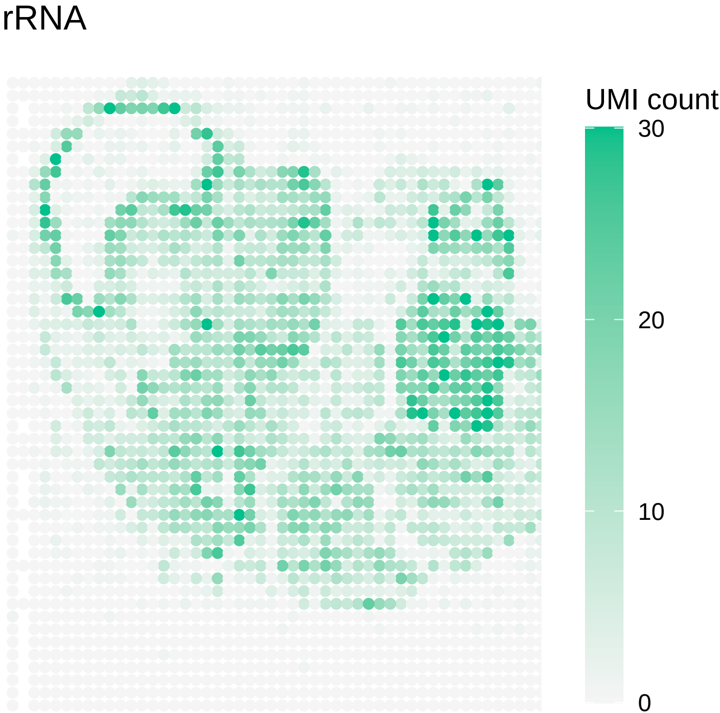
rRNA
UMI count
30
20
10
0

scaRNA

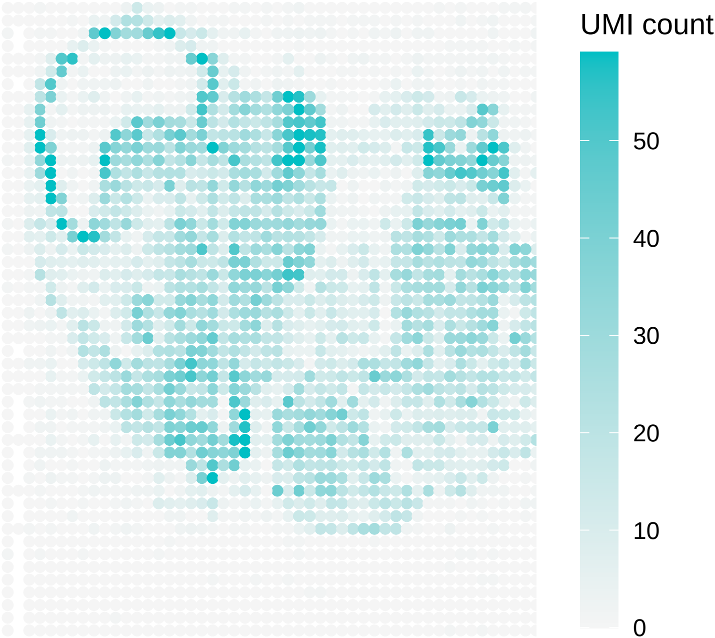

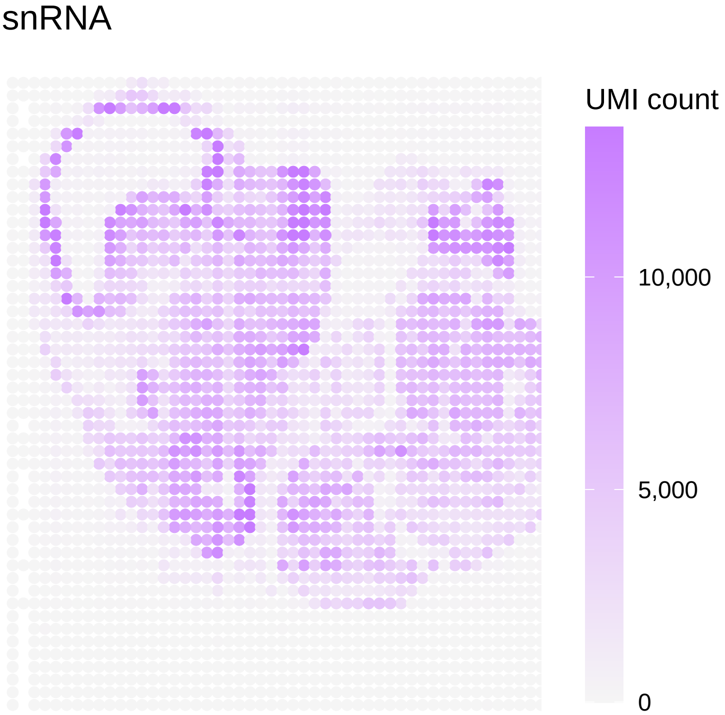
snRNA
UMI count
10,000
5,000
0

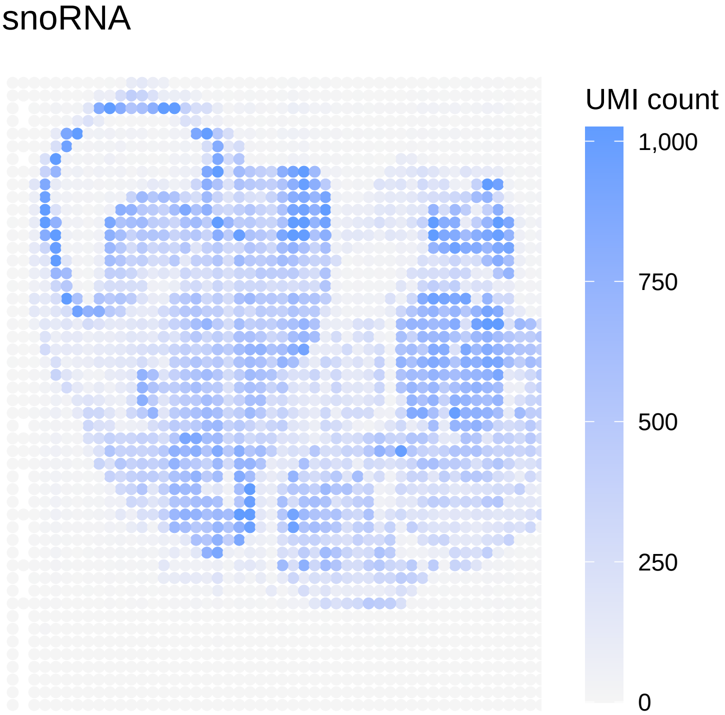
snoRNA
UMI count
1,000
750
500
250
0
