# Supplementary material for: ASTRO: Automated Spatial-Transcriptome whole RNA Output
Source: Bioinformatics. 2026 Jan 6;42(2):btaf688. doi: 10.1093/bioinformatics/btaf688 (PMC12866646; doi:10.1093/bioinformatics/btaf688)

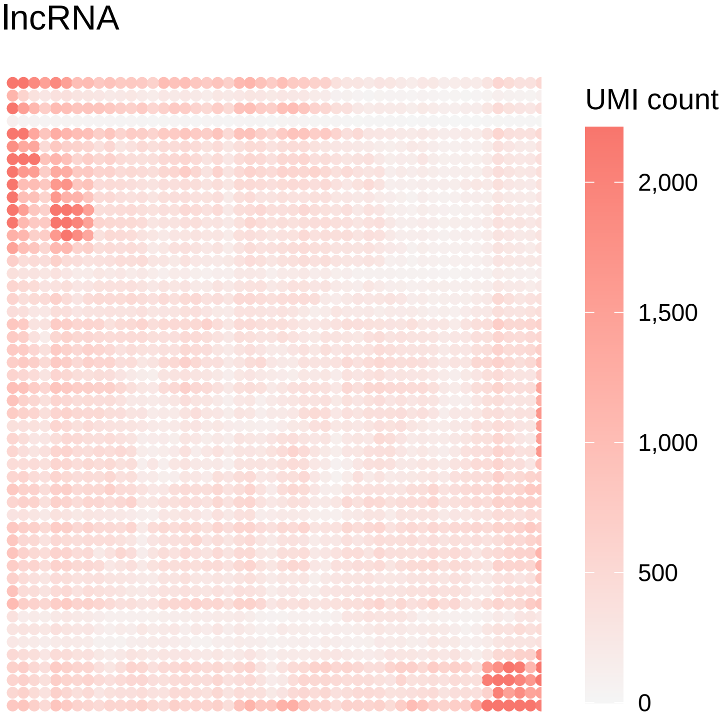
lncRNA
UMI count
2,000
1,500
1,000
500
0

miRNA

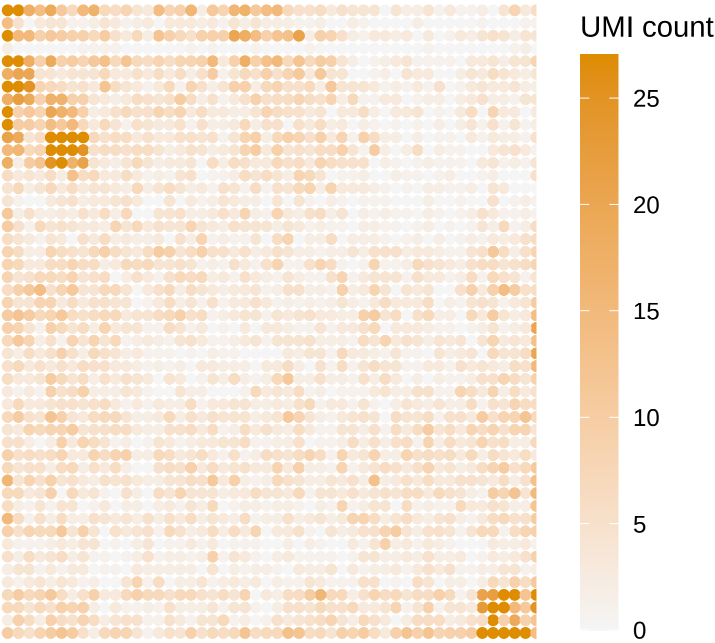

misc_RNA

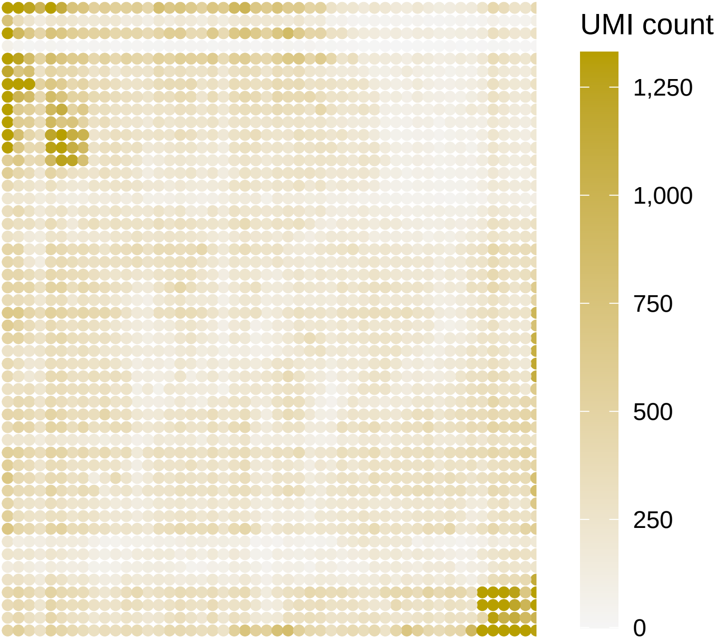

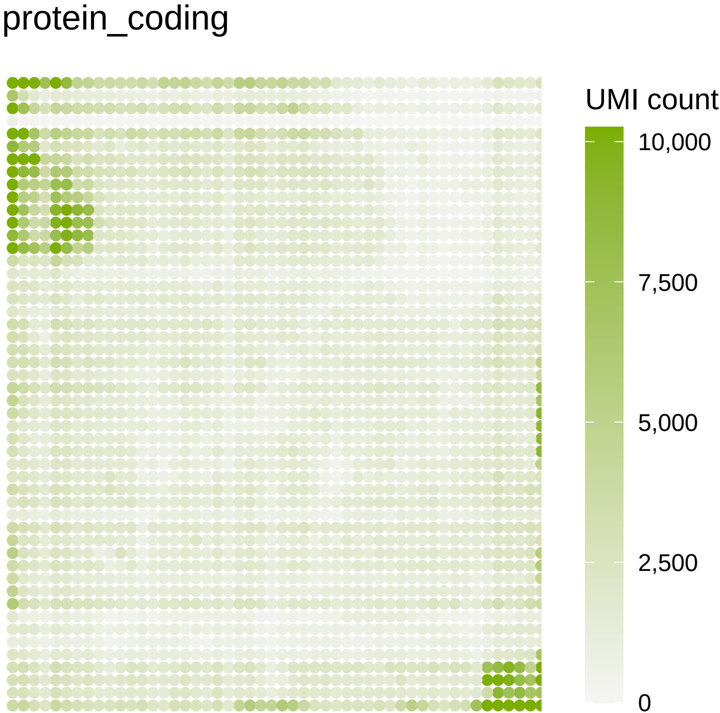
protein_coding
UMI count
10,000
7,500
5,000
2,500
0

pseudogene

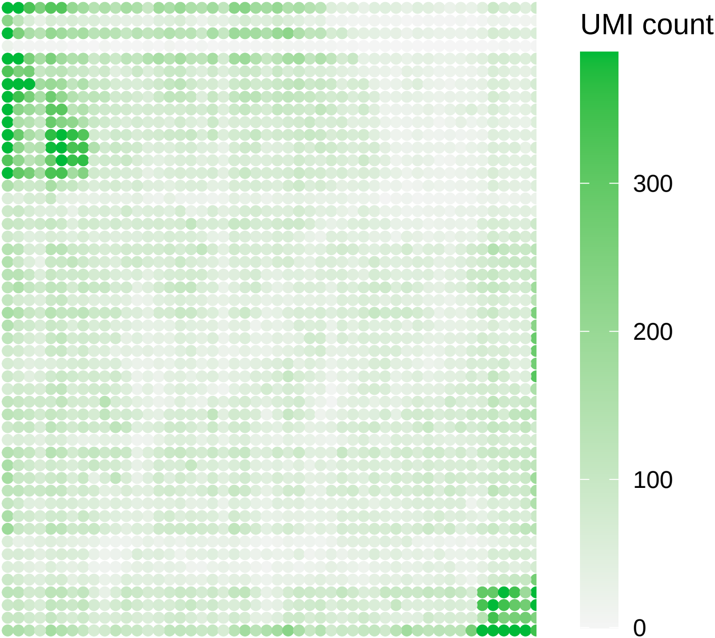

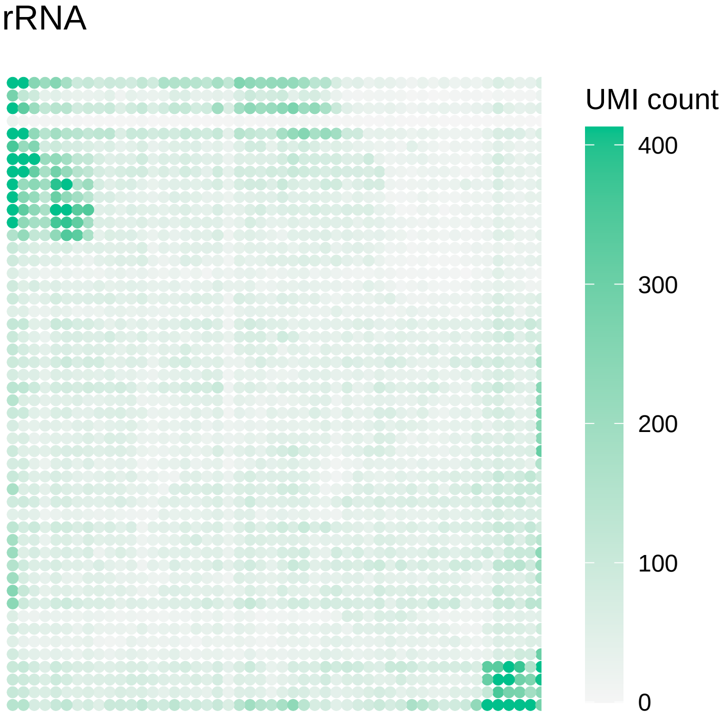
rRNA
UMI count
400
300
200
100
0

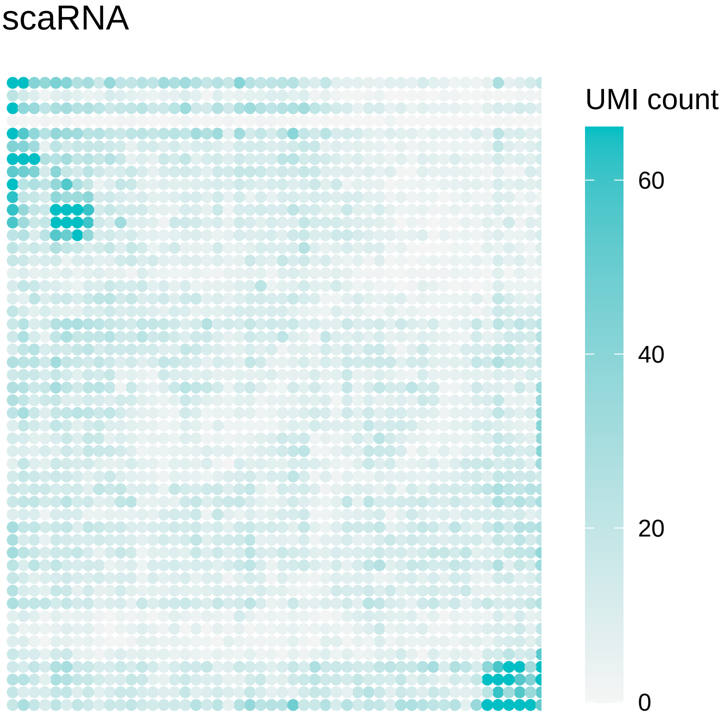
scaRNA
UMI count
60
40
20
0

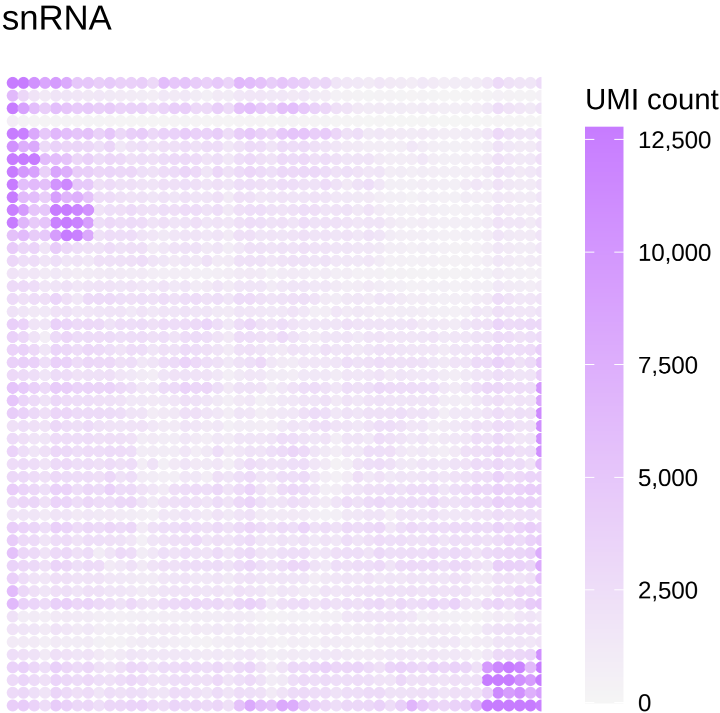
snRNA
UMI count
12,500
10,000
7,500
5,000
2,500
0

snoRNA

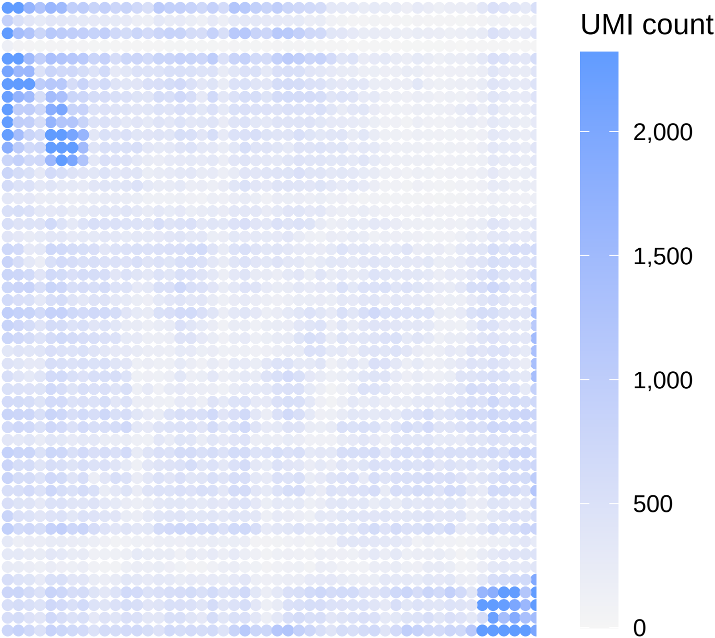

Supplement: btaf688_Supplementary_Data [file btaf688_supplementary_data.zip › Supplementary File 1.zip!/Supplementary File 1/Spatial_mapping/the_number_of_UMI/ST-pipeline/Healthy donor lymph node.pdf]
