# Supplementary material for: ASTRO: Automated Spatial-Transcriptome whole RNA Output
Source: Bioinformatics. 2026 Jan 6;42(2):btaf688. doi: 10.1093/bioinformatics/btaf688 (PMC12866646; doi:10.1093/bioinformatics/btaf688)
Supplement: btaf688_Supplementary_Data [file btaf688_supplementary_data.zip › Supplementary File 1.zip!/Supplementary File 1/Spatial_mapping/the_number_of_UMI/ASTRO/Mouse Embryo 1.pdf]

Y_RNA

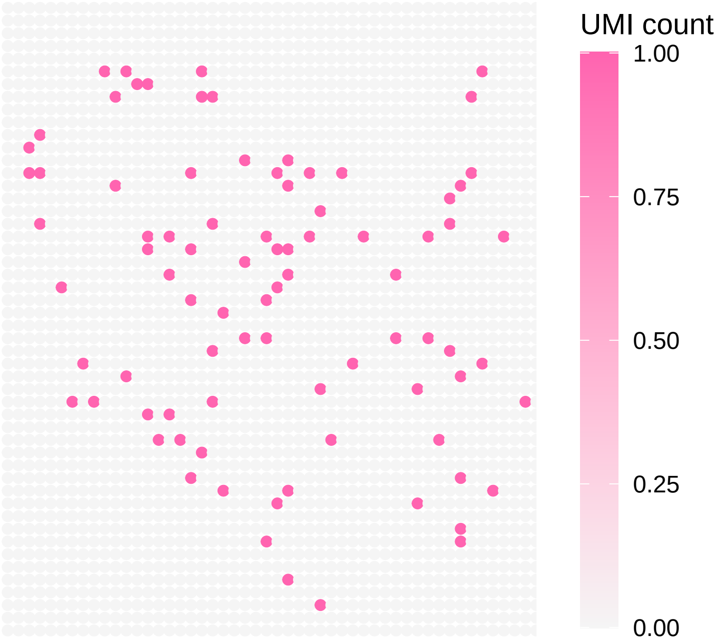

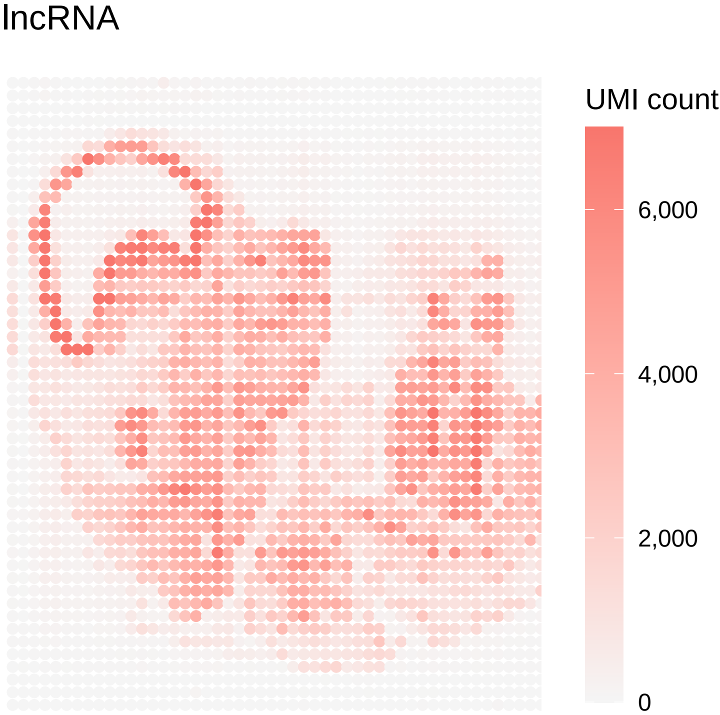
lncRNA
UMI count
6,000
4,000
2,000
0

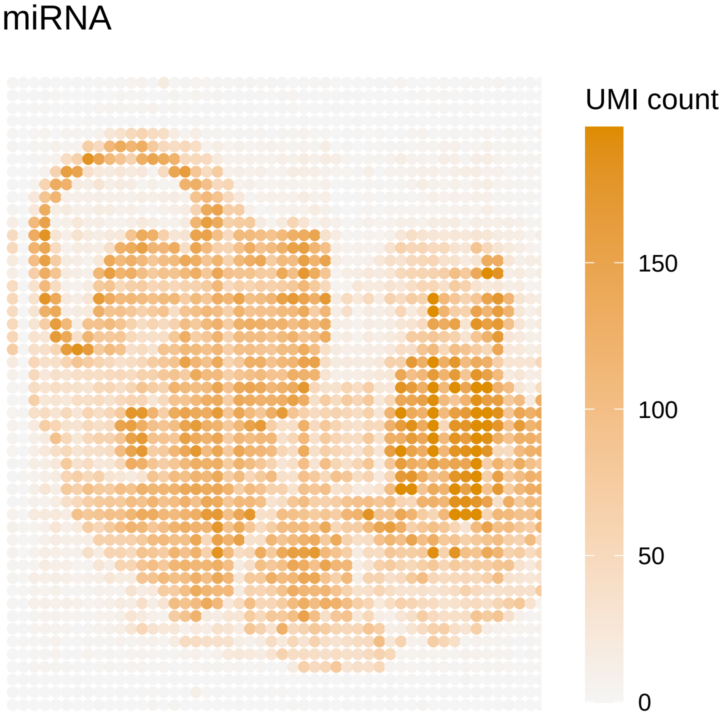

miRNA
UMI count
150
100
50
0

misc_RNA

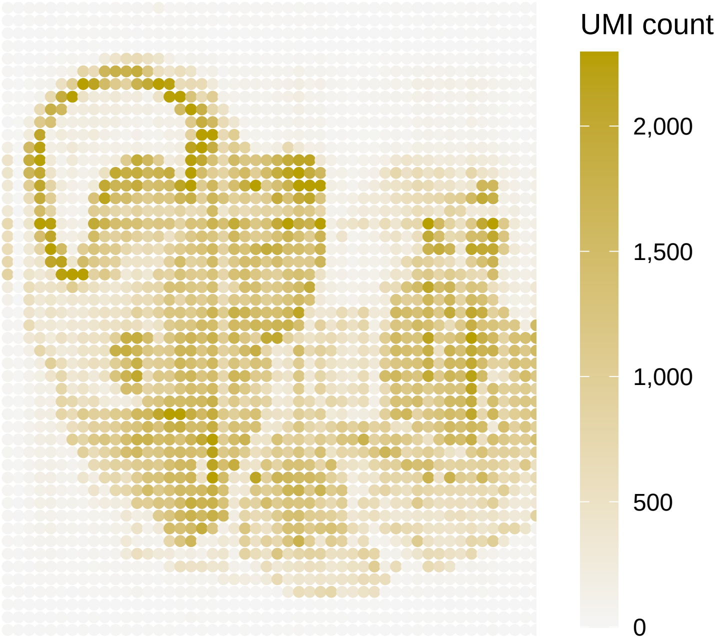

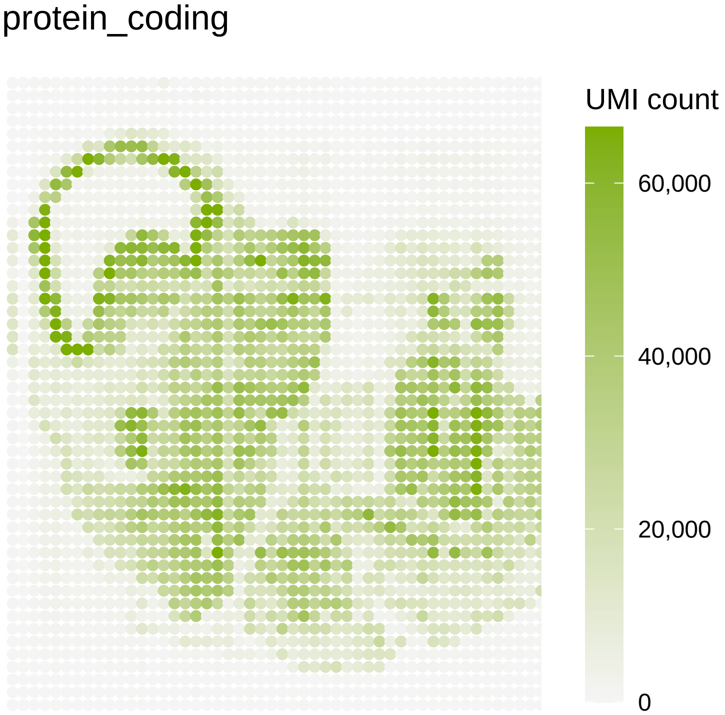

protein_coding
UMI count
60,000
40,000
20,000
0

pseudogene

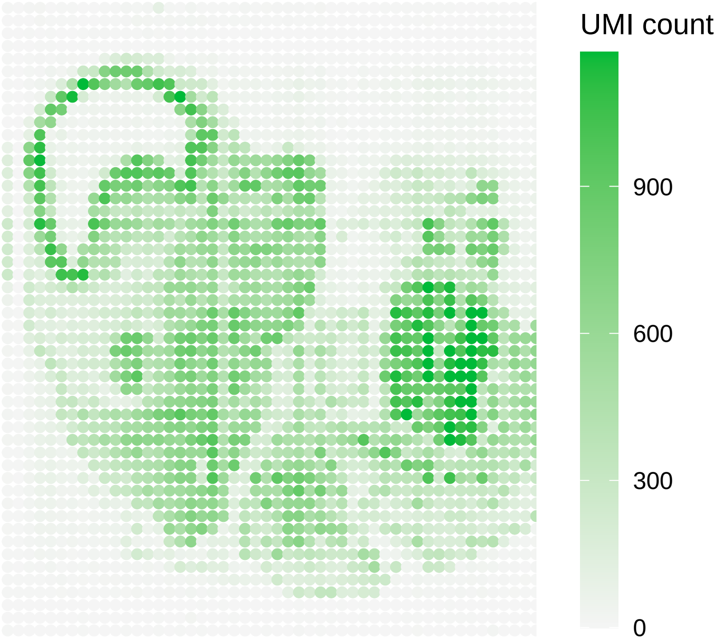

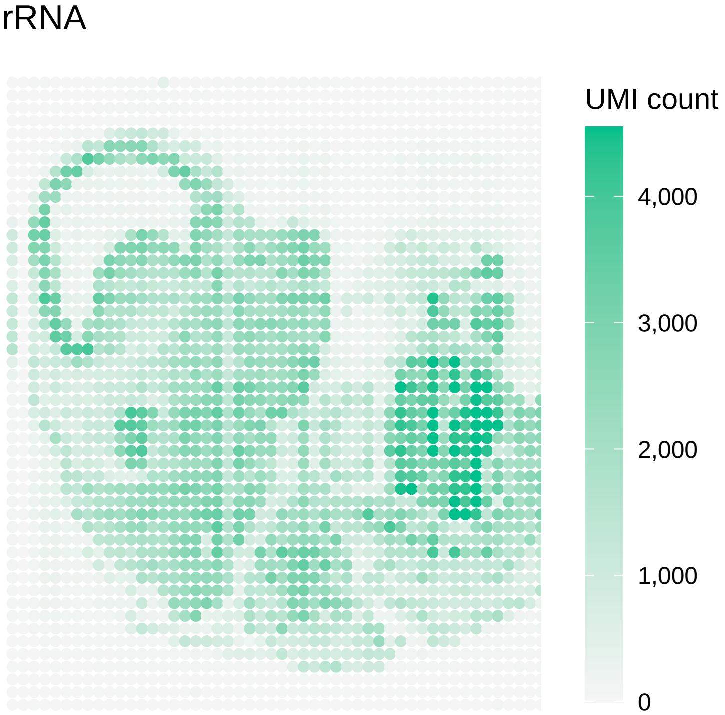
rRNA
UMI count
4,000
3,000
2,000
1,000
0

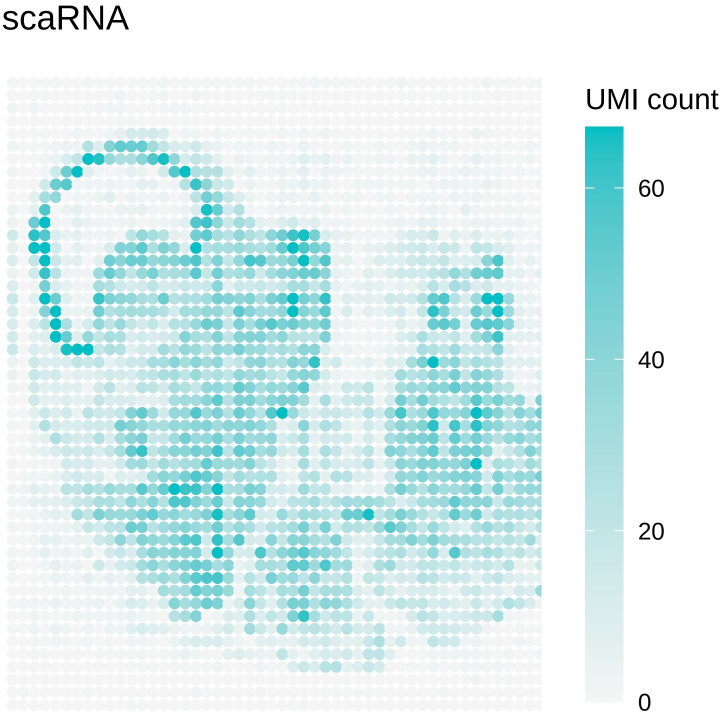
scaRNA
UMI count
60
40
20
0

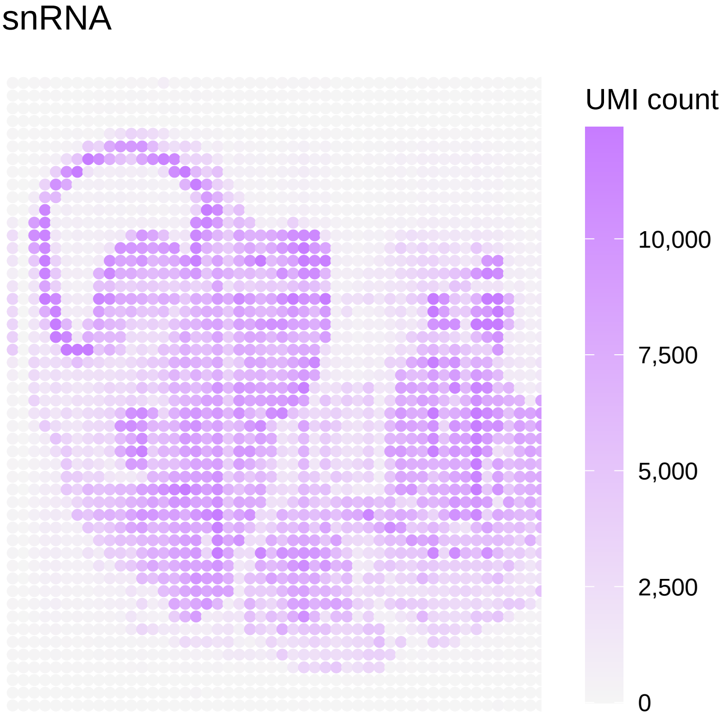

snRNA
UMI count
10,000
7,500
5,000
2,500
0

snoRNA

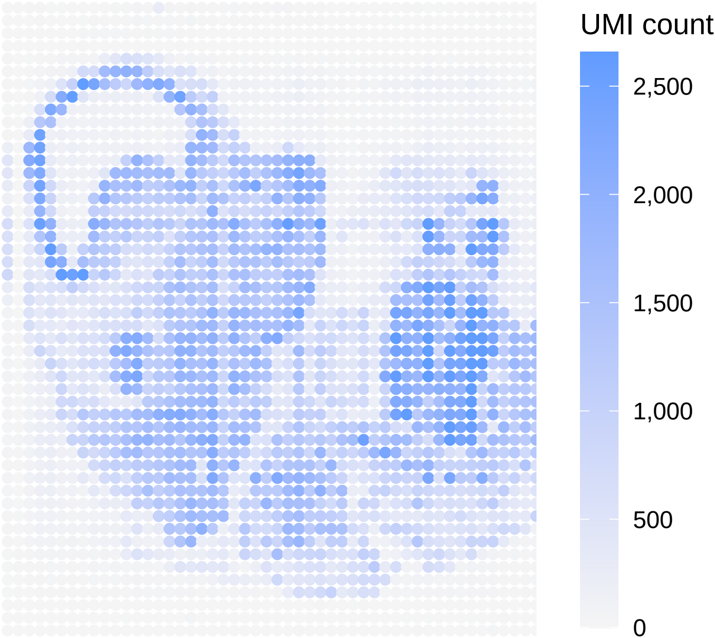

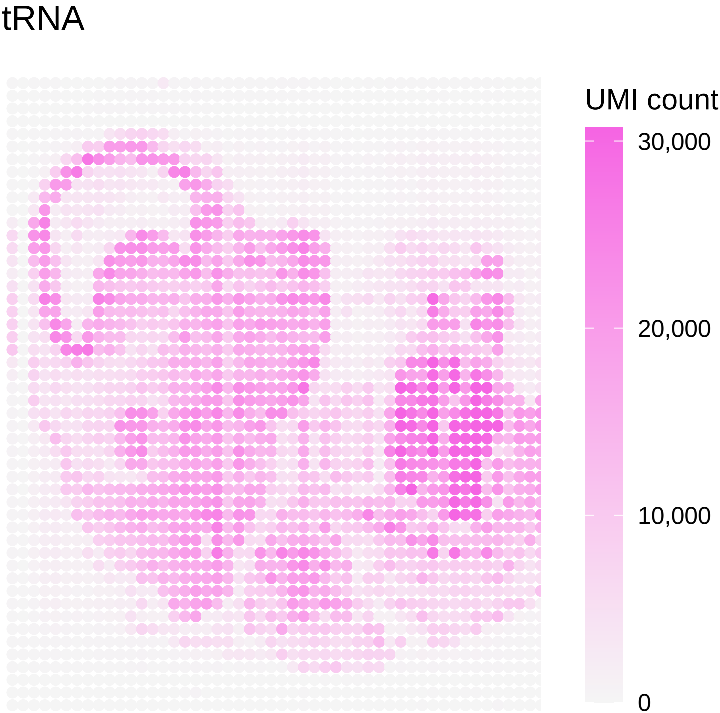
tRNA
UMI count
30,000
20,000
10,000
0
