# Supplementary material for: ASTRO: Automated Spatial-Transcriptome whole RNA Output
Source: Bioinformatics. 2026 Jan 6;42(2):btaf688. doi: 10.1093/bioinformatics/btaf688 (PMC12866646; doi:10.1093/bioinformatics/btaf688)
Supplement: btaf688_Supplementary_Data [file btaf688_supplementary_data.zip › Supplementary File 1.zip!/Supplementary File 1/Spatial_mapping/the_number_of_UMI/ASTRO/Mouse Embryo 2.pdf]

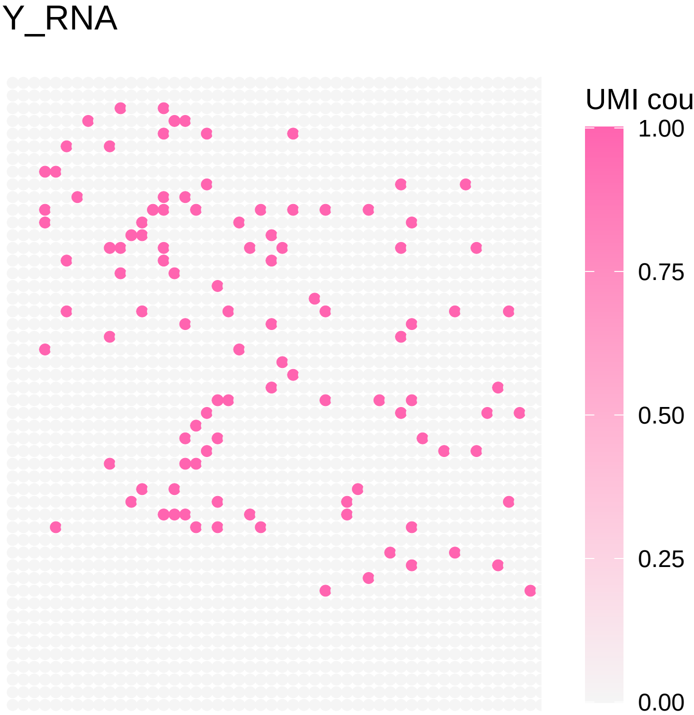
Y_RNA
UMI count
1.00
0.75
0.50
0.25
0.00

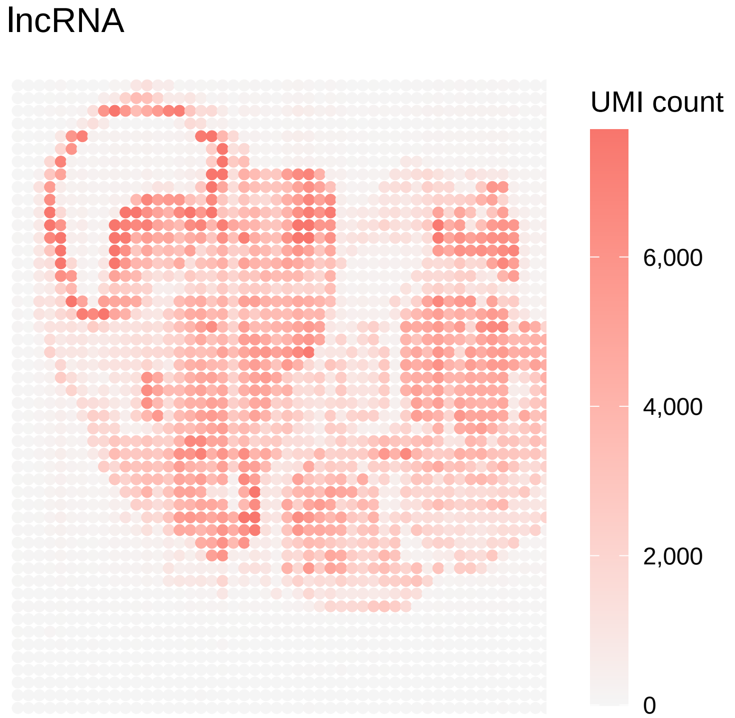
lncRNA
UMI count
6,000
4,000
2,000
0

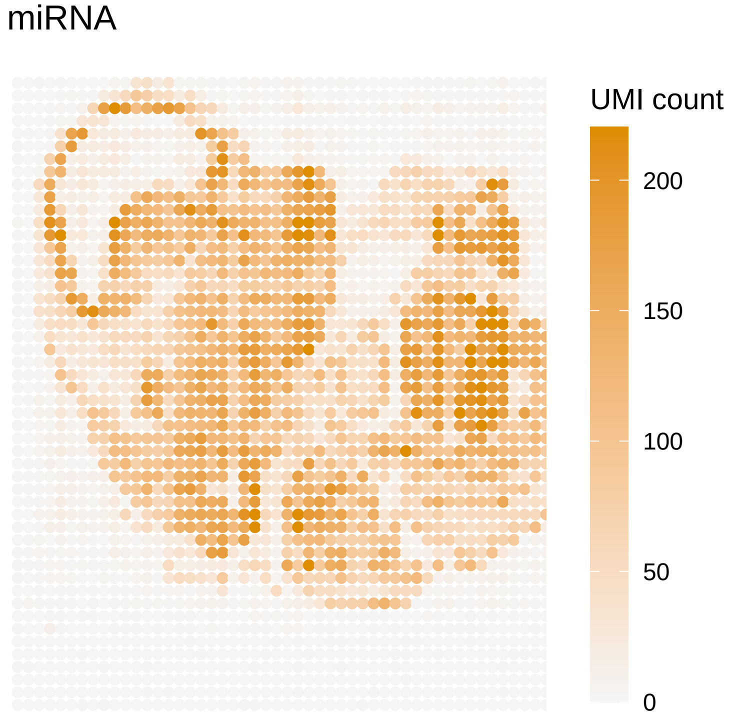

miRNA
UMI count
200
150
100
50
0

misc_RNA

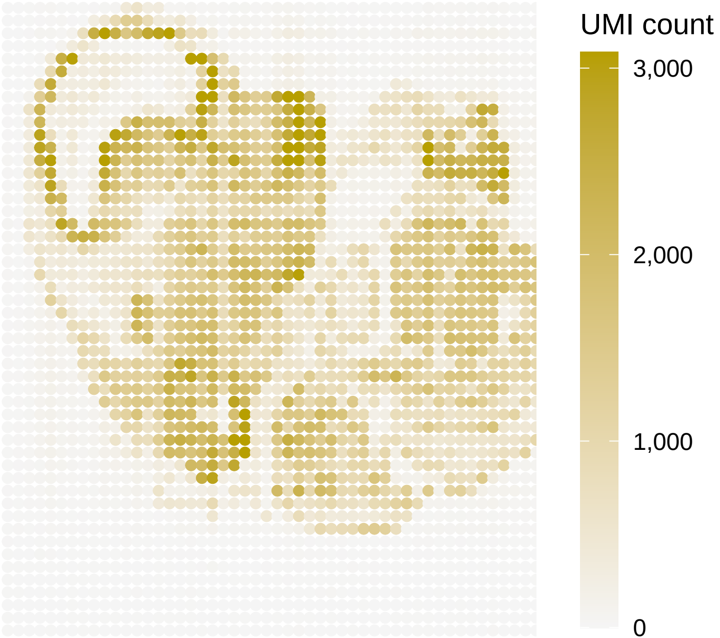

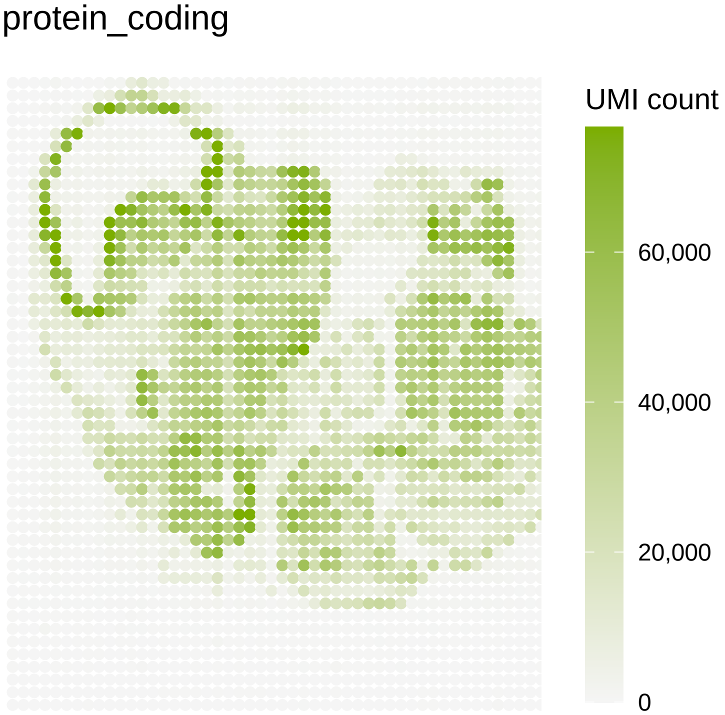
protein_coding
UMI count
60,000
40,000
20,000
0

pseudogene

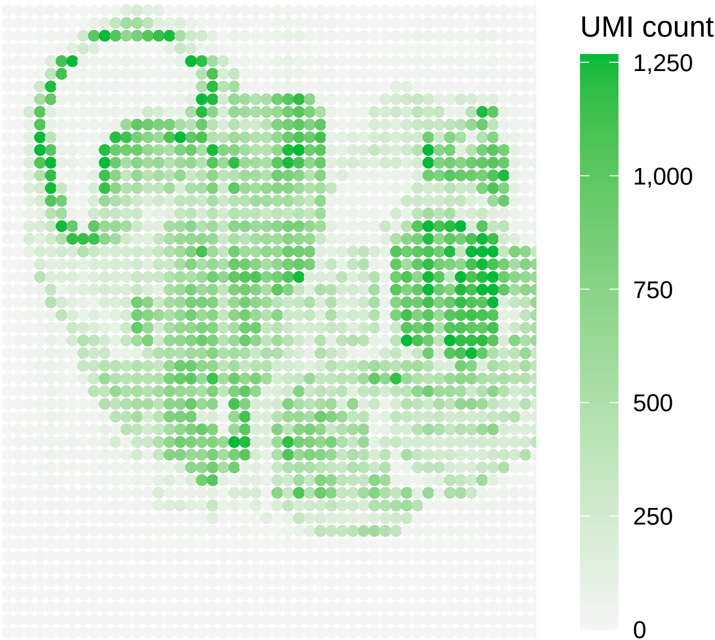

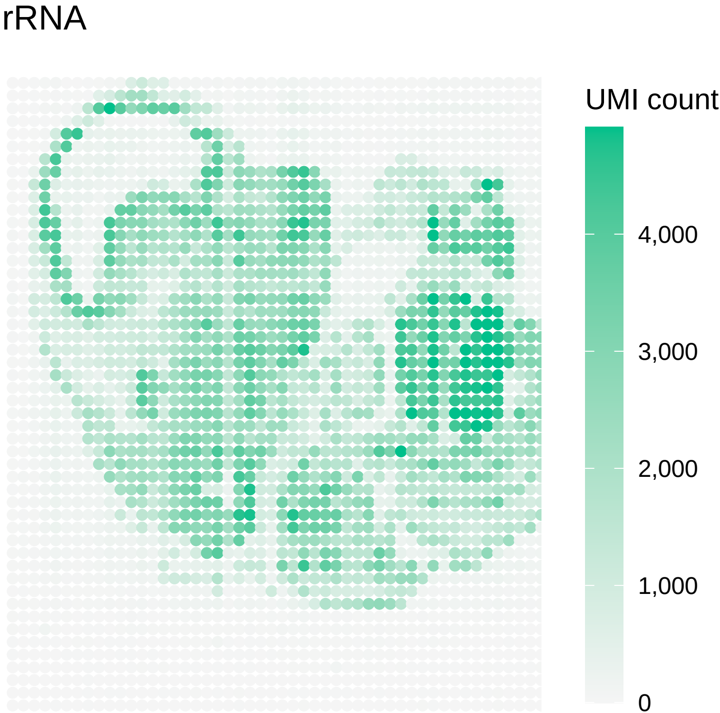

rRNA
UMI count
4,000
3,000
2,000
1,000
0

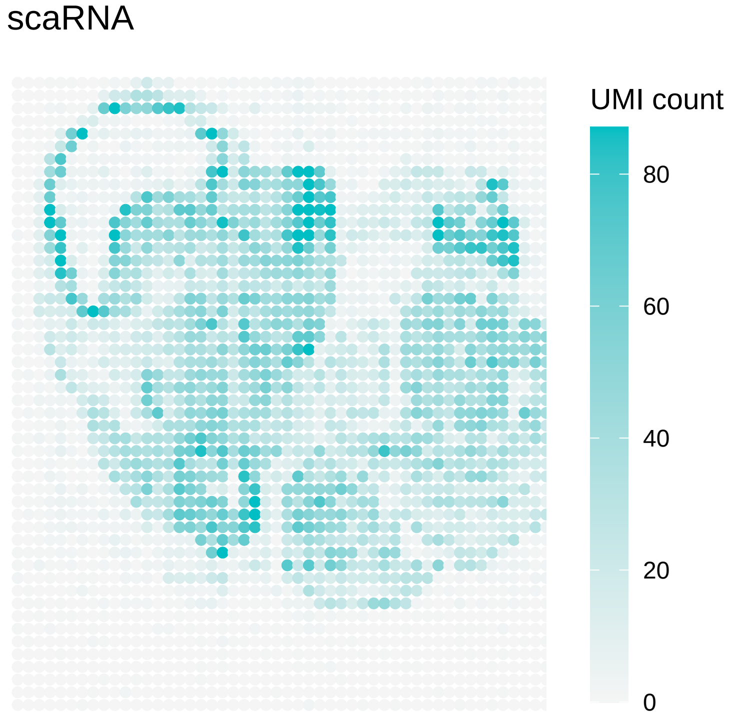
scaRNA
UMI count
80
60
40
20
0

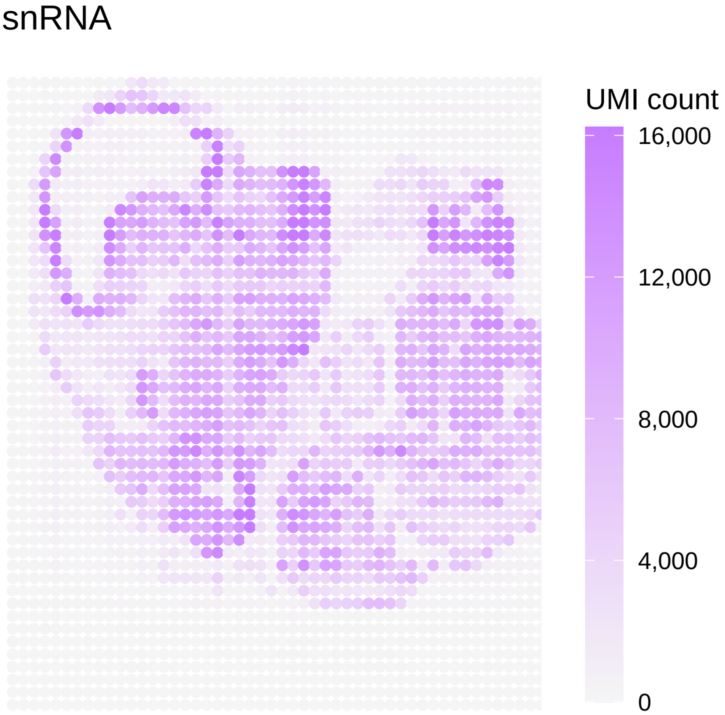
snRNA
UMI count
16,000
12,000
8,000
4,000
0

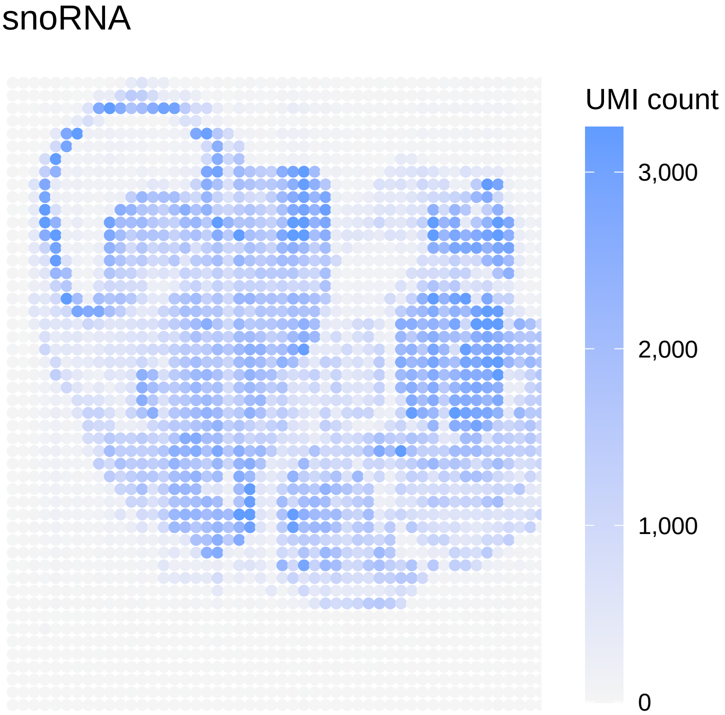
snoRNA
UMI count
3,000
2,000
1,000
0

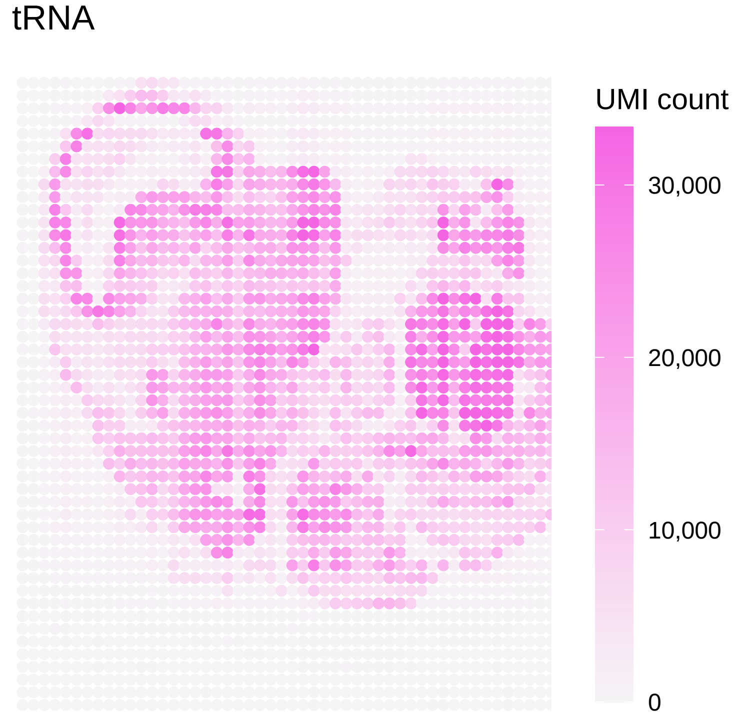
tRNA
UMI count
30,000
20,000
10,000
0
