# Supplementary material for: ASTRO: Automated Spatial-Transcriptome whole RNA Output
Source: Bioinformatics. 2026 Jan 6;42(2):btaf688. doi: 10.1093/bioinformatics/btaf688 (PMC12866646; doi:10.1093/bioinformatics/btaf688)
Supplement: btaf688_Supplementary_Data [file btaf688_supplementary_data.zip › Supplementary File 1.zip!/Supplementary File 1/Spatial_mapping/the_number_of_UMI/ASTRO/Healthy donor lymph node.pdf]

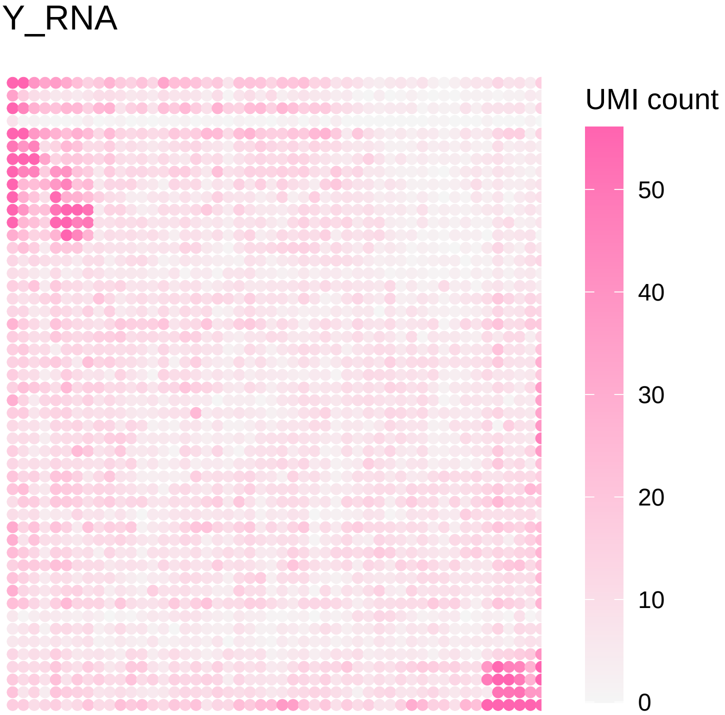

Y_RNA
UMI count
50
40
30
20
10
0

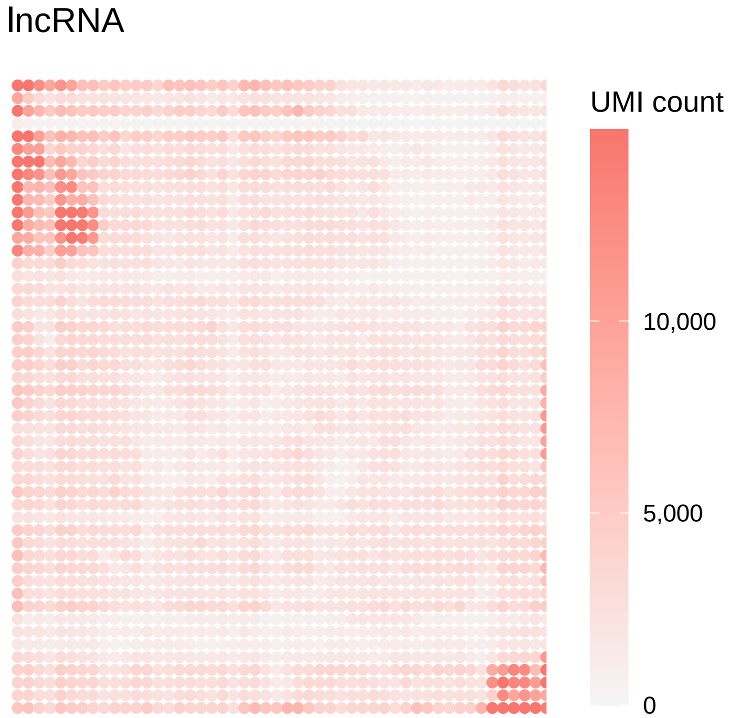
lncRNA
UMI count
10,000
5,000
0

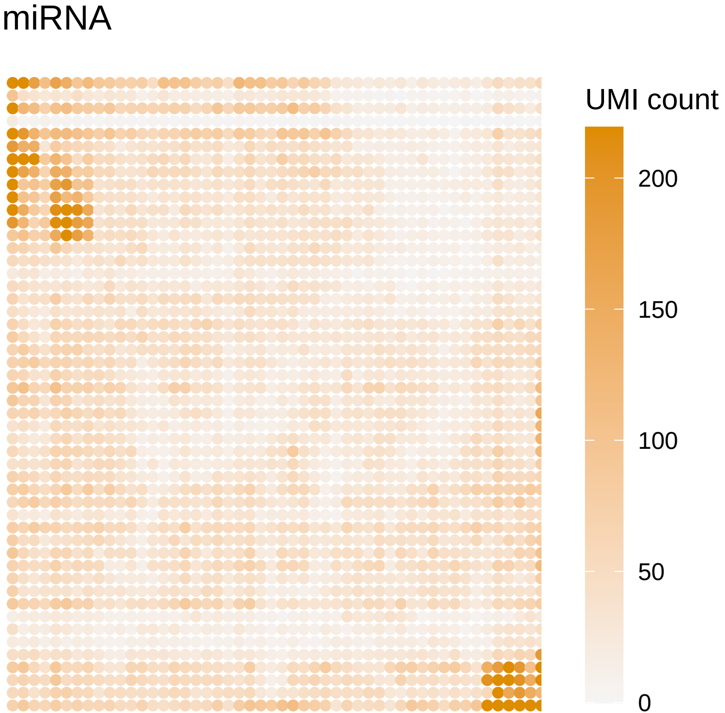
miRNA
UMI count
200
150
100
50
0

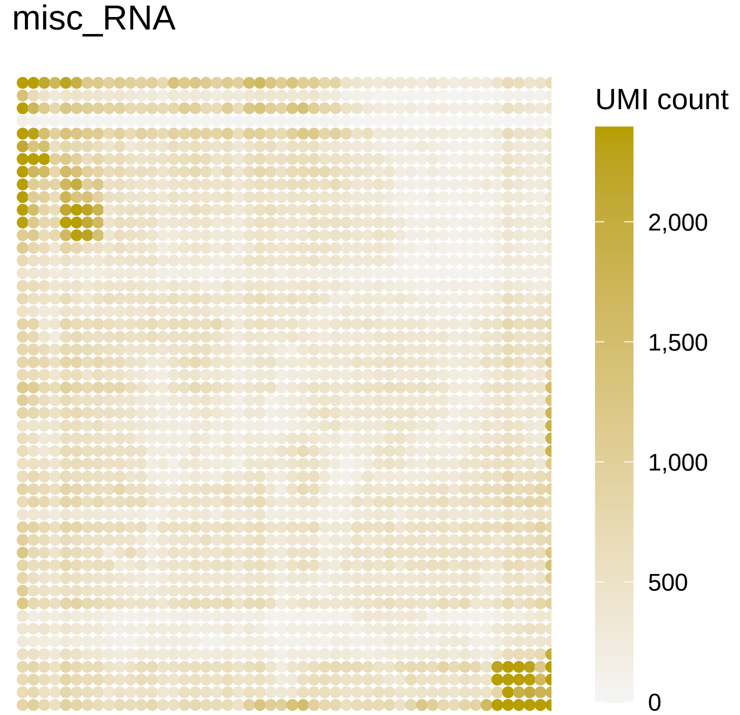
misc_RNA
UMI count
2,000
1,500
1,000
500
0

protein_coding

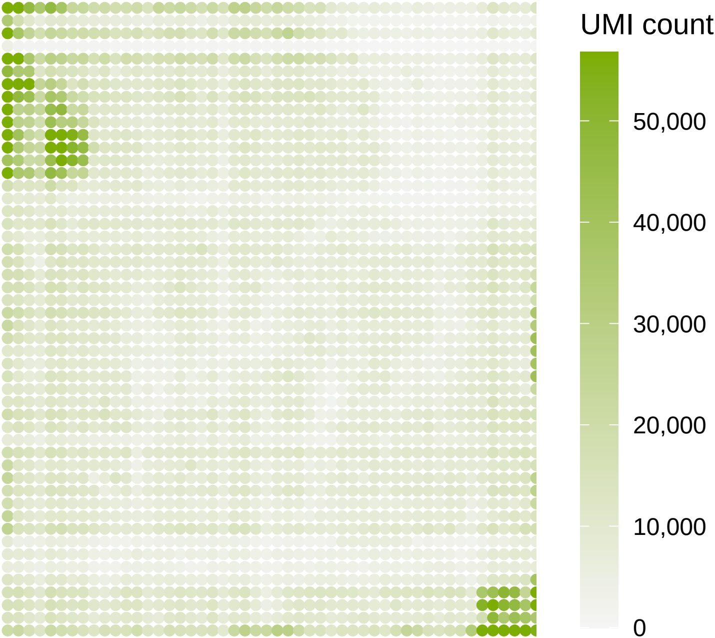

pseudogene

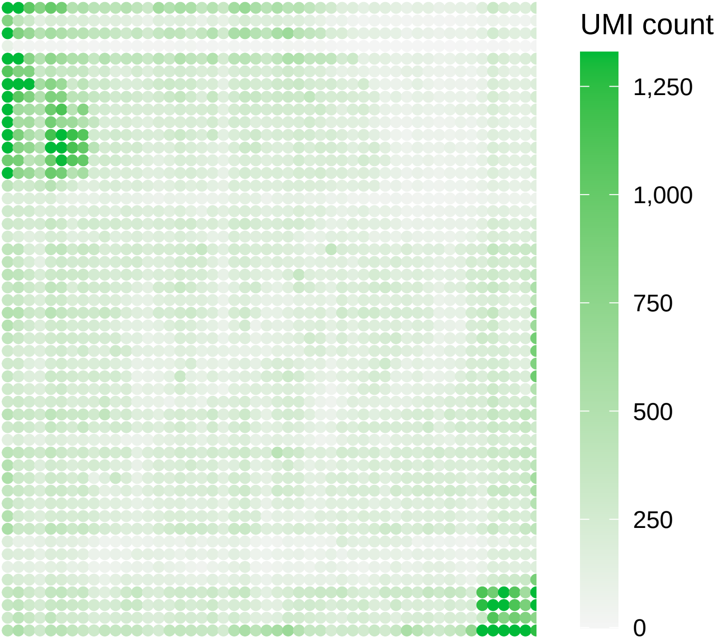

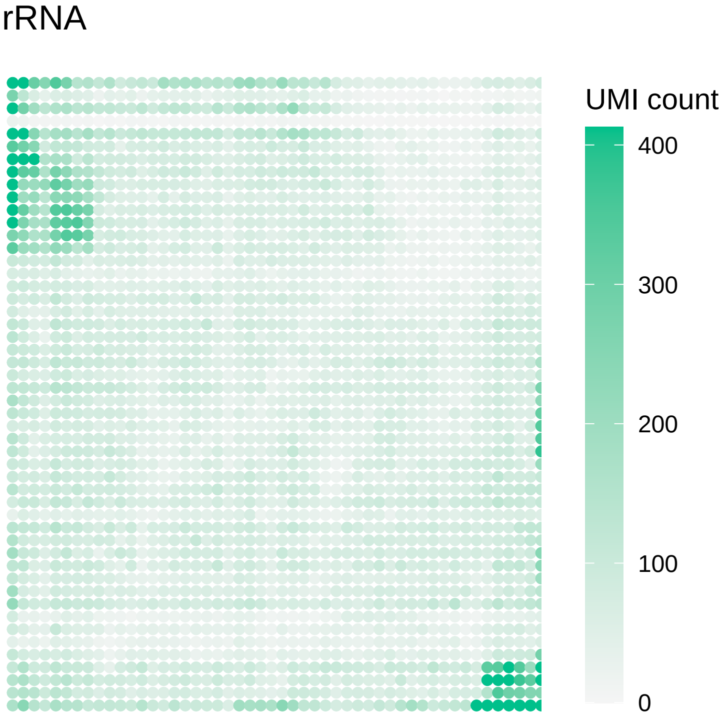
rRNA
UMI count
400
300
200
100
0

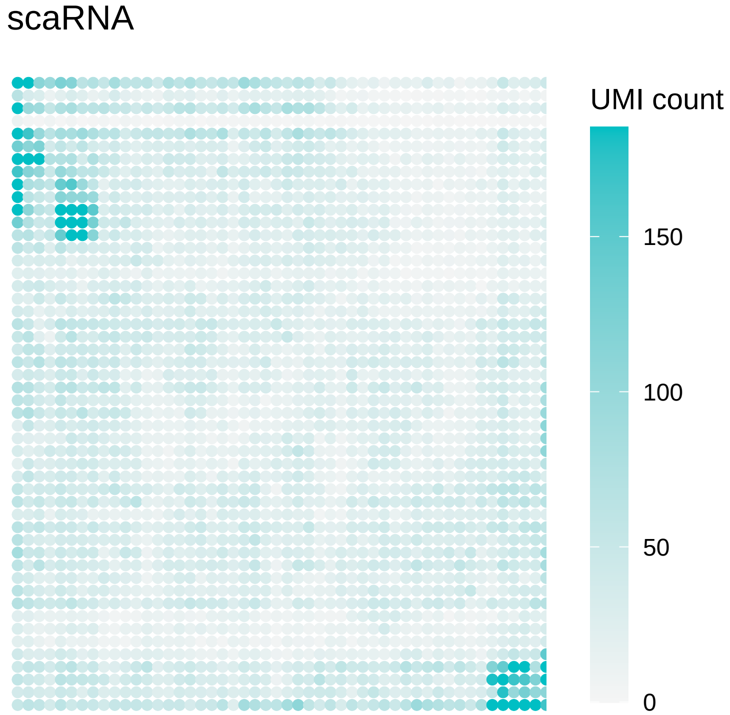
scaRNA
UMI count
150
100
50
0

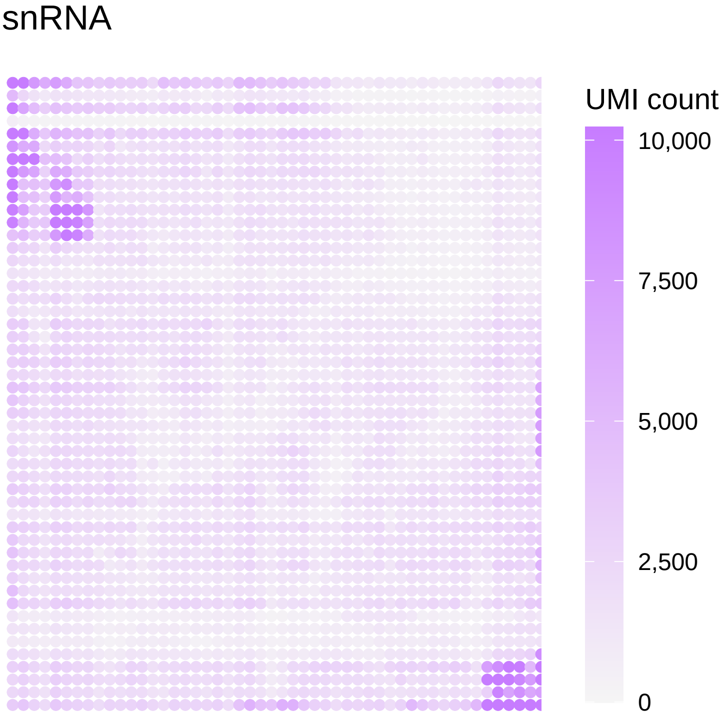
snRNA
UMI count
10,000
7,500
5,000
2,500
0

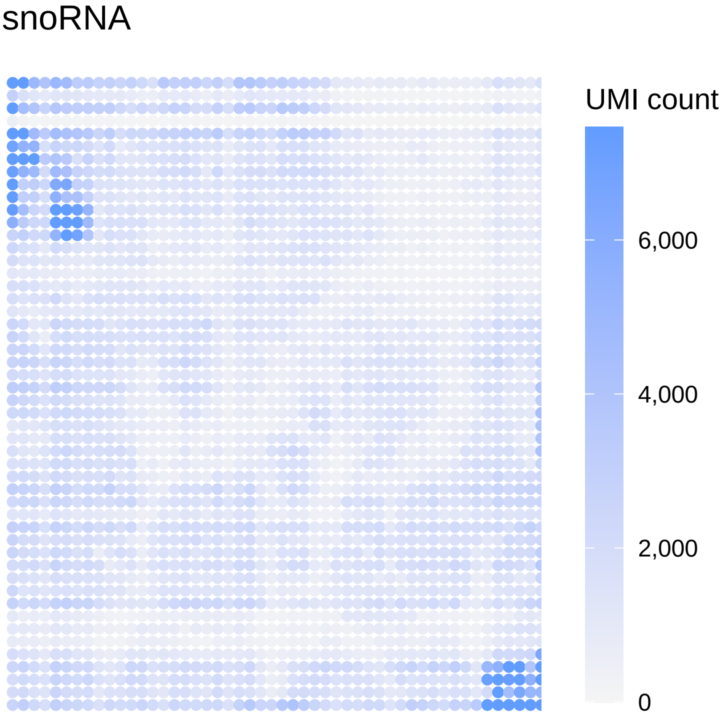
snoRNA
UMI count
6,000
4,000
2,000
0

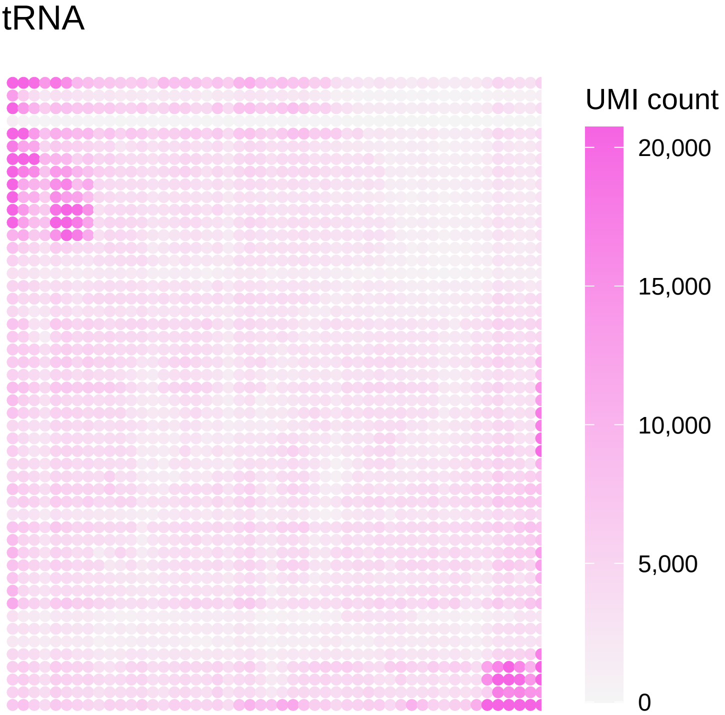
tRNA
UMI count
20,000
15,000
10,000
5,000
0
